# An Integrated Coral Reef Ecosystem Model to Support Resource Management under a Changing Climate

**DOI:** 10.1371/journal.pone.0144165

**Published:** 2015-12-16

**Authors:** Mariska Weijerman, Elizabeth A. Fulton, Isaac C. Kaplan, Rebecca Gorton, Rik Leemans, Wolf M. Mooij, Russell E. Brainard

**Affiliations:** 1 Joint Institute for Marine and Atmospheric Research, University of Hawaii at Manoa, Honolulu, Hawaii, United States of America; 2 Environmental Systems Analysis Group, Wageningen University, Wageningen, Netherlands; 3 Pacific Island Fisheries Science Centre, NOAA Fisheries, Honolulu, Hawaii, United States of America; 4 Oceans and Atmosphere Flagship, CSIRO, Hobart, Tasmania, Australia; 5 Northwest Fisheries Science Centre, NOAA Fisheries, Seattle, Washington, United States of America; 6 Department of Aquatic Ecology, Netherlands Institute of Ecology (NIOO-KNAW), Wageningen, Netherlands; 7 Aquatic Ecology and Water Quality Management Group, Wageningen University, Wageningen, Netherlands; Biodiversity Research Center, Academia Sinica, TAIWAN

## Abstract

Millions of people rely on the ecosystem services provided by coral reefs, but sustaining these benefits requires an understanding of how reefs and their biotic communities are affected by local human-induced disturbances and global climate change. Ecosystem-based management that explicitly considers the indirect and cumulative effects of multiple disturbances has been recommended and adopted in policies in many places around the globe. Ecosystem models give insight into complex reef dynamics and their responses to multiple disturbances and are useful tools to support planning and implementation of ecosystem-based management. We adapted the Atlantis Ecosystem Model to incorporate key dynamics for a coral reef ecosystem around Guam in the tropical western Pacific. We used this model to quantify the effects of predicted climate and ocean changes and current levels of current land-based sources of pollution (LBSP) and fishing. We used the following six ecosystem metrics as indicators of ecosystem state, resilience and harvest potential: 1) ratio of calcifying to non-calcifying benthic groups, 2) trophic level of the community, 3) biomass of apex predators, 4) biomass of herbivorous fishes, 5) total biomass of living groups and 6) the end-to-start ratio of exploited fish groups. Simulation tests of the effects of each of the three drivers separately suggest that by mid-century climate change will have the largest overall effect on this suite of ecosystem metrics due to substantial negative effects on coral cover. The effects of fishing were also important, negatively influencing five out of the six metrics. Moreover, LBSP exacerbates this effect for all metrics but not quite as badly as would be expected under additive assumptions, although the magnitude of the effects of LBSP are sensitive to uncertainty associated with primary productivity. Over longer time spans (i.e., 65 year simulations), climate change impacts have a slight positive interaction with other drivers, generally meaning that declines in ecosystem metrics are not as steep as the sum of individual effects of the drivers. These analyses offer one way to quantify impacts and interactions of particular stressors in an ecosystem context and so provide guidance to managers. For example, the model showed that improving water quality, rather than prohibiting fishing, extended the timescales over which corals can maintain high abundance by at least 5–8 years. This result, in turn, provides more scope for corals to adapt or for resilient species to become established and for local and global management efforts to reduce or reverse stressors.

## Introduction

The future of coral reefs and the economic and societal benefits they provide are uncertain. Reefs are increasingly affected by local human-induced disturbances and climate change. Managing and understanding the consequences of these stressors and maintaining the reefs’ high biodiversity, productivity and multitude of dynamic interactions necessitate an integrated ecosystem approach. This complexity also challenges assessing management outcomes [1, 2]. Comprehensive, integrated ecosystem modeling is a useful tool to gain insight into reef dynamics while considering the multiple interacting stressors to these ecosystems [3, 4]. The utility of model projections depends on the model’s ability to simulate key processes and components of the reef ecosystem and how these are influenced by and respond to different disturbances and management scenarios. To better address societal objectives, ecosystem models should also consider the socioeconomic consequences of changes in ecosystem state. Whole-of-system or end-to-end models differ from previous models by comprising the entire ecosystem, including the human component, and the associated abiotic processes extending through to climate change effects. These models also dynamically couple or integrate physical and biological processes at different time scales making them more realistic [5, 6].

During an international coral reef stakeholder workshop [7, 8], four economically important ecosystem services were identified: (1) shoreline protection, which is influenced by the structural complexity of a reef system; (2) tourism and recreational opportunities, which are influenced by turbidity (land-based sources of pollution) and algal and faunal communities; (3) production of fish; and (4) production of other natural products. Furthermore, stakeholders identified maximizing reef ecosystem integrity as a key objective [8, 9]. Most existing coral reef models focus on biological feedback mechanisms (e.g., [10, 11]), though a smaller subset of ecosystem models include physical and biological disturbances [12–14] and human uses (fisheries) [11, 15, 16]. Only a few models dynamically integrate socioeconomic and biophysical processes [17–20], which is necessary for exploring potential changes in the coral reef ecosystem services identified by stakeholders. A comparison study of coral reef ecosystem models suitable as a decision-support tool for management, suggested the Atlantis framework would be most appropriate [21]. The suitability of the Atlantis framework for assessing the impacts of interactions between species and fisheries and their implications for marine fisheries management [21–24] was also suggested by an ecosystem modeling review study [25].

Effective ecosystem-based management relies upon understanding the relative ecosystem effects of multiple disturbances acting concurrently [26]. The relative impact of each of these disturbances on reef status is uncertain, and this uncertainty hinders cost-effective reef management and conservation [27]. Ecosystem models, such as Atlantis, can simulate these disturbances simultaneously and allow for the exploration both of their impacts individually and their interactive and cumulative effects. By improving our understanding of these interacting influences on the ecosystem, we can gain insights into how better to manage human activities associated with coral reef ecosystems.

In this paper, we use the developed Guam Atlantis Coral Reef Ecosystem Model (Guam Atlantis; on-line S1 Text and [28]) to explore the interactive effects of three main drivers: climate change, land-based pollution sources of pollution (LBSP, i.e., additional nutrients and sediments to the ecosystem) and fishing. We simulate the effects of two and three drivers simultaneously and analyze the interactive effects on the reef ecosystem surrounding Guam. Additionally, to make the model output more relevant to resource managers, we assess whether local management strategies can mitigate the effects of climate change as has been suggested in other studies [2, 3, 29, 30]. In a companion paper (currently in review at PloSONE) we compliment this approach by using the Guam Atlantis model to consider a series of scenarios with different levels of fishing and LBSP, similar to other ecosystem modeling efforts [31, 32] that evaluate the socio-ecological tradeoffs of alternative management scenarios.

## Methods

### Modeling framework

The Atlantis framework consists of spatially explicit, three-dimensional irregular polygons or boxes and, for each box and water layer, incorporates information on the biological, geochemical, and physical forcings [22, 33]. Atlantis integrates these dynamics through two-way coupling and combines them with the effects of different human user groups (fisheries, oil extraction and mining or coastal development). Atlantis dynamically tracks the interaction of all these factors over time and its simulations use a simple forward difference integration scheme to solve a system of differential equations typically on a 12-h time step (finer adaptive sub-steps are executed for high turn-over rate groups such as plankton). The dynamic processes are user specific and many alternative model formulations can be selected to set complexity at a desired level. Fulton et al. [24] gives an overview of the modular structure of Atlantis and more information can be found on the Atlantis-wiki (best found by searching for ‘Atlantis CSIRO’) and in other publications of the application of the Atlantis ecosystem model, such as, Griffith et al. [34]. Here we briefly discuss the spatial and oceanographic modules and the adaptations made to the ecological module.

### Guam Atlantis model components

#### Spatial module

**Fig 1:**
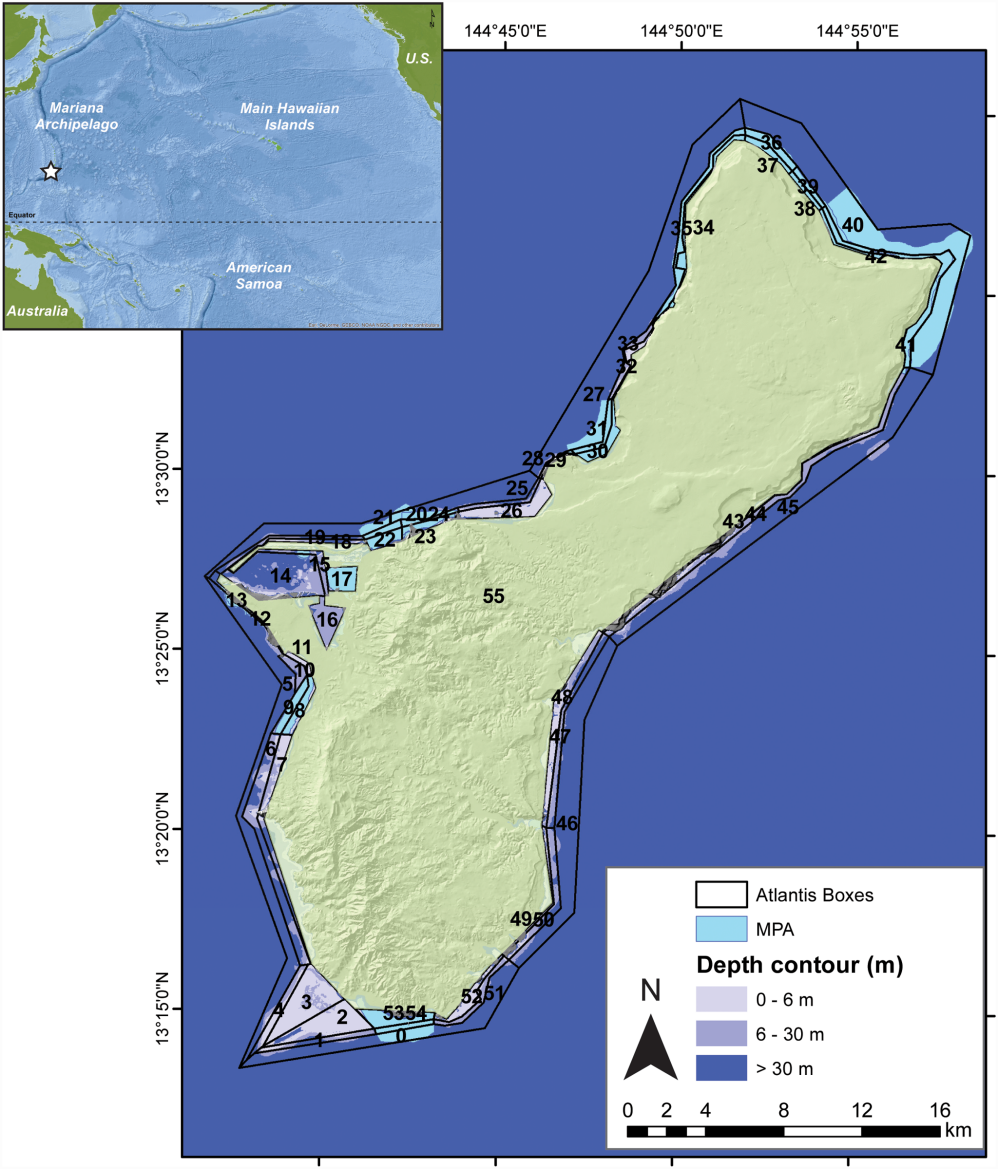
Spatial polygons of Guam Atlantis.

We apply the Guam Atlantis model for the historical period of 1985–2014 to calibrate the model and then simulate the period 2015–2050. Briefly, the Guam Atlantis model incorporates spatially-differentiated habitats (polygons) and vertical stratification (i.e., water layers) allowing for the representation of hydrodynamic and biological processes (e.g., migration of fish to different habitat types in their lifecycles, larval connectivity between reef areas, vertebrate movement between polygons and water layers (S2 Text)). Preliminary discussions with ecologists and coral reef managers in Guam led to the use of two depth layers: 0–6 m and 6–30 m. We limited the model domain to this shallow (< 30 m) depth range of the reef system due to the availability of biomass and diversity data for species in these depth layers, and the relative paucity of data from deeper habitats. Biological data for the initial conditions are primarily from NOAA’s Pacific Islands Fisheries Science Center, Coral Reef Ecosystem Division (CRED) supplemented with data from Guam Coastal Zone Management Program, University of Guam Marine Laboratory, Guam Division of Aquatic and Wildlife Resources (DAWR) and Guam Environmental Protection Agency. Outer polygons (30–100 m) are included for oceanic forcing (nutrient import/export, larval connectivity, and water, heat and salinity fluxes). Based on the benthic habitat, fish assemblages, prevailing oceanographic conditions, fisheries reporting zones and the existing managed areas, we delineated 55 marine spatial units, 25 shallow, 23 deep and 7 outer boundary boxes (Fig 1).

We delineated 25 shallow (< 6 m), 23 deep (6–30 m) dynamic boxes, 7 non-dynamic boundary boxes (outer-most 7 polygons), for the advection of nutrients and plankton, and the island of Guam as a non- active box. The star in the inset map shows the location of Guam in the western Pacific Ocean. Polygons with nutrient and/or sediment inputs are numbered 3, 7, 8, 10, 16, 17, 22, 23, 24, 26, 30, 32, 48, 49, 52, and 53. Data source inset map came from ArcGIS-online (ArcGIS and ArcMap are the intellectual property of Esri and are used herein under license) and the topography of Guam island from USGS [35].

#### Oceanographic module

The oceanographic module consists of two main data inputs based on a Regional Ocean Modeling System (ROMS) developed for the Coral Triangle (CT) in the western Pacific Ocean [36] (downloaded from http://www.ctroms.ucar.edu/, October 24, 2014). The main focus of this CT-ROMS model was the larger Coral Triangle region, but the model domain includes Guam at its northern boundary. The data used from the CT-ROMS model include: (1) horizontal fluxes (to estimate the magnitude and direction of the currents) that cross each face (or side) of the Atlantis polygons per daily time step, and (2) average vertical velocity, temperature and salinity per Atlantis polygon per daily time step. The available data were from 1957–2007 with a spatial resolution of 5 km. We extracted the grid points around Guam from 1985–2007 to calculate horizontal velocity, solar irradiance, temperature and salinity. The last year of data was repeated for years 2008–2050. Vertical velocity was not included in the CT-ROMS model output, hence, simulated values were created by taking random values with a mean of zero and standard deviation taken from field data [37–39].

#### Ecological module

The reef’s ecological module differs from most coral reef models developed to date, as it is process based and uses empirical parameterizations of basic metabolism (e.g., production, consumption, waste) and ecological dynamics instead of derived parameters, such as productivity over biomass and consumption over biomass [40]. Furthermore, trophodynamic flows are fully coupled and the detrital pathways (both in the water column and in the sediment layer) are explicitly modeled. To represent those pathways, we included 42 functional groups consisting of 3 detrital, 2 bacteria, 5 plankton, 3 algal, 3 sessile invertebrate, 6 mobile invertebrate and 20 vertebrate groups (S1 Table). To accommodate improved understanding of reef resilience we grouped fishes by their life-history characteristics, habitat preferences and diet in the following functional groups: piscivores, corallivores, invertivores, planktivores, detritivores and herbivores. We have further classified the herbivores by their ecological roles as excavators/bio-eroders, scrapers, grazers and browsers [41–45]. Based on the biomass of each species in 2011 [46], we took the weighted mean of species-specific data on diet and life history parameters (e.g., growth rate, natural mortality, maximum age, age at maturity, size of recruits, length of pelagic larval development, von Bertalanffy growth coefficients, swim speed) for the overall estimation of those parameters for each functional group (S1 Table). Biomass estimates, spatial distribution, and fisheries data are detailed further in Weijerman et al. [28].

We avoided aggregating fished and unfished species into the same functional groups, and identified fishery target species based on shore-based creel surveys conducted since 1985 by Guam’s Division of Aquatic and Wildlife Resources (DAWR) [47]. We chose to limit the fishery data to shore-based creel surveys and not include the boat-based creel surveys as we assumed that the shore-based fishery took place entirely in our model domain while the boat-based fishery is mostly focused on trolling and demersal fishing in deeper waters [46]. Although this may have been an erroneous assumption (see S1 Text for exploration of this topic). In addition to these living and detritus groups, ammonia, nitrate and silica are represented dynamically. The model’s initial conditions represent 1985 (after a 10-year‘burn-in' phase) and we projected this forward for 30–65 years under the set of scenarios described below.

The coral framework is the foundation for coral reef ecosystems; hence, corals are integrally linked to most reef dynamics. Corals are consumers by night and photosynthesize by day, but are also a source of food to corallivorous invertebrates and fishes. Due to their three-dimensional structure, corals also provide habitat and shelter for many reef species [48, 49]. Even dead corals continue to harbor diverse communities until erosion processes unbalanced by growth lead to the loss of three-dimensional structure [50].

Coral species have different life history dynamics and sensitivities to environmental factors (e.g., sediments, elevated temperature, disease) that influence mortality and growth. We grouped corals into massive and encrusting corals (‘massive’)—with lower growth rates and a lower sensitivity to stressors—and branching/tabular/columnar corals (‘branching’)—with higher growth rates and higher sensitivity to stressors [51, 52].

After a literature review we identified key coral reef dynamics and the form of the relationships for those dynamics and added corresponding code (Table A1 in S1 Text). These relationships were derived from empirical data or from other modeling studies. Coral specific parameters are included in S2 Table. We detailed the dynamics of coral growth (and growth-related complexity) and competition with benthic algae that are influenced by three main drivers (Fig A1 in S1 Text): (1) climate change (a global stressor); (2) LBSP (a local stressor); and fishing activities (also a local stressor). We acknowledge that we have only captured the main processes in any degree of detail and have omitted or simplified other processes (e.g., symbionts’ dynamics [13], microbe-induced coral mortality [53], coral and algal diseases [54, 55], linear relationship between herbivore size and bio-erosion [10] and others).

### Model validation

In the on-line supporting information (S1 Text) and Weijerman et al. [28], the methods and assumptions made for the development of this integrated coral reef ecosystem model are described in detail. A critical aspect of this includes description of data sources, calibration and validation. In previous applications of Atlantis models, corals were simply modeled as benthic filter feeders [32, 56–59]. We added code that addresses how corals are affected by (1) climate change (including ocean warming and acidification), (2) changes in land use (eutrophication and sedimentation) and (3) fishing activities. By including extensive empirical data collected from field studies in Guam, local-scale dynamics are projected over decades, and trends that will likely manifest themselves locally are identified. We carefully validated the model in two ways, first by examining the model behavior over 30–75 years without any disturbances, i.e., a ‘control’ system, and secondly by comparing model projections for historical periods to available abundance time series, following guidelines for Atlantis model development [58–60]. The only available time series data were those of the reconstructed fish biomass from 1985 to 2011 which was based on catch-per-unit-effort (CPUE) fishery data [47]. Weijerman et al [47]used the CPUE data and fishery-independent visual estimates of fish biomass from 2009–2011 to reconstruct the relative biomass of eight functional species groups exploited in the fishery. Since we had no time series data of the impacts of other drivers (LBSP, climate change) on the coral or fish biomass at the scale of our entire model domain, we evaluated the model skill with a widely accepted method that involves pattern matching [61]. With pattern matching, coral biomass trajectories from simulations of each of the disturbances (climate change, sediments, and nutrients) were compared with results of empirical studies from particular sites in Guam or from regional sites if local information was not available (S1 Text).

We performed sensitivity analyses by investigating key dynamics in coral reef ecosystems as identified by empirical and theoretical studies (S1 Text). These included (1) coral-algal space competition that structures the reef benthos; (2) primary productivity that influences the energy flow; and (3) structural complexity that determines the shelter capacity of the reef framework for juvenile and small fishes and hence influences fish biomass and diversity (S1 Text). Results from these analyses showed that uncertainty in primary productivity had the greatest influence on the model outcomes (Figs A17-A19 in S1 Text). Therefore, we ran additional simulations with the bounded parameter values for growth rates of small and large phytoplankton [62, 63]. This uncertainty was incorporated into our analysis of the impacts of the individual stressors on the coral reef ecosystem.

Model validation, verification and sensitivity analyses are further discussed in the online S1 Text. One important caveat stemming from this is that the effects of acidification are likely underestimated by the model. A second caveat, as stated above, is that coral biomass is sensitive to the growth rate of primary producers. Improving the relationship between reef organisms and acidification, obtaining more accurate downscaled time series of projected change in pCO_2_ and obtaining better growth rates and biomass estimates of phytoplankton communities will likely enhance the model’s capabilities to make projections. Additionally, the model skill in estimating fish biomass had a clear bias and overestimated a number of groups (Fig A20 in S1 Text). More research is necessary to explain the bias and then correct for it (e.g., better fishery data, diet data of apex predators, recruitment data for the overestimated fish groups). However, with the current information available it is still possible to make relative comparisons.

### Simulations and model output

To explore the effects of individual stressors relative to a ‘no-disturbances’ scenario we first simulated a ‘control’ run (S1 Text) that did not include any of the identified stressors. The outcomes of this control run were subsequently compared with outcomes from runs with the added stressors: climate change (i.e., ocean warming and acidification), LBSP and fishing. Models were run for 30 years (1985–2015) to explore the effect of each of the stressors on the present conditions and for 65 years (1985–2050) to explore the effect of these stressors on future conditions. We supplemented these ‘main’ simulations with simulations parameterized with high and low phytoplankton growth rates, which illustrate uncertainty in the model outcome due to uncertainty in primary productivity.

Predicted changes in atmospheric CO_2_ concentrations came from the IPCC Fifth Assessment Report using the highest emission scenario, Representative Concentration Pathway (RCP) 8.5 projection [64]. These increased CO_2_ concentrations for emission scenario RCP8.5 led to a decrease in the oceanic pH which in turn led to a reduction in the aragonite saturation state. This resulted in reduced calcification rates modeled as a reduced growth rate of corals (S1 Text which also explains relationships between pH and several other organisms).

Predicted sea surface temperature data also came from the RCP8.5 projection using the HadGEM-AO model output (data downloaded from the Coupled Model Intercomparison Project Phase5 (CMIP5): http://apdrc.soest.hawaii.edu/las8/UI.vm), as the historical 1985–1990 modeled data corresponded well with satellite-derived SST data for Guam during the same time period. We overlaid this trend on the existing time series of temperature from the CT-ROMS model output [36] for each Atlantis grid (or polygon) and created a projected temperature time series for each grid cell out to 2050 while maintaining spatial differences around Guam.

Land-based sources of pollution (LBSP) were modeled as additional input of nitrogen and sediments into coastal polygons that had riverine runoff or sewage outflow pipes (S1 Text, [28]). The sediment and nutrient loads were based on data collected from 2005–2011 (Guam Environmental Protection Agency, War-of-the-Pacific National Park and CRED) and used as initial condition input data for the model. River flow and additional nutrient and sediment input data were based on outflow time series from 1991 and 2011 and the last year was repeated for future projections. These outflow time series did not show any temporal trend (Fig A13 in S1 Text) and, for simplicity, we assumed no future changes in land-use or the amount of rain fall (and hence river out flow).

Fishing mortality was calculated for each functional group based on the historical catches from shore-based creel surveys conducted by DAWR. Effort stayed constant between the early 1985–1990 period and the recent 2007–2011 period [46] and for simplicity we assumed fishing mortality stayed constant at 2010–2012 levels (S1 Text). In a companion paper (currently in review at PlosOne) we varied levels of LBSP and fishing to evaluate the socio-ecological tradeoffs of alternative management scenarios with the Guam Atlantis model.

Ecosystem metrics used to score the effect size were based on performance indicators for reef resilience [62, 65, 66], plus one additional indicator: the ratio of biomass of targeted species in the recreational reef fishery at the end relative to the start of a simulation (Table 1). We used that metric as a proxy for one source of socioeconomic benefits from the reef ecosystem—availability of preferred target fishery species.

**Table 1:** Ecosystem metrics used to determine effect size of simulation of scenario runs.

Metric	Description	Rationale
Ratio of benthic calcifiers to non-calcifiers	Ratio of total biomass of corals and crustose coralline algae (CCA) to total biomass of turf and fleshy macroalgae in the model domain	Corals form the framework of coral reef ecosystems and CCA the ‘glue’ that cements the reef together; a high ratio of calcifiers to non-calcifiers implies a more structurally complex system that provides more desirable ecosystem goods and services than a macroalgal-dominated (flat) system [62]
Mean trophic level of the community	Biomass-weighted average of the trophic level of all functional groups in the ecosystem.	Indication of maturity for ecosystems; higher value represents more ecosystems [62]
Biomass of apex predators	Sum of biomass of apex predator groups (sharks, roving piscivores, benthic piscivores and mid-water piscivores).	Indication of ‘health’ for ecosystems; higher value represents ‘healthier’ ecosystem. In general, more apex predators decrease community susceptibility to perturbations [65].
Biomass of herbivorous fishes	Sum of biomass of all herbivorous fish groups.	Indication of resilience with more herbivores leading to less chance of ecosystem shifts to undesirable algal-dominated state [66].
Total biomass (excluding detritus)	Sum of biomass of all species.	Indication of maturity/stability of ecosystems; higher value represents more mature or stable ecosystem [62]
Ratio of biomass of target fishery species at the end to the start of a simulation	Total biomass of all fish species that are targeted in the reef fishery at the end vs start of simulation run.	Indicator of socioeconomic condition; higher value means higher availability of target species to recreational fisherman

Ecosystem metrics were calculated as the average over the last three years of a simulation, to smooth over intra-and inter-annual variation, and results for each Atlantis polygon were summed to get results for the entire model domain. For each of the six ecosystem metrics, the response ratio was calculated as the ratio of the metric under a scenario (e.g., climate change) relative to the value of that metric in the control run, following the methods in Kaplan et al. [67]. The interactive effects among the drivers were explored to see if their combined effect led to higher values (negative interaction) or lower values (positive interaction) than would be expected based on the sum of the impacts of the individual drivers [67]. To determine the interactive effect size another two simulations were conducted: one with two drivers acting simultaneously (adding the two stressors with the largest effects individually) and the other with all three drivers. For instance, if two drivers individually caused a 2% and 3% decline in a metric, the additive expectation of combining both drivers is a 5% decline. If simultaneously applying the drivers actually led to only a 4% decline, the interaction is slightly positive; if simultaneously applying the drivers led to a 6% decline, the interaction is slightly negative. Based on the assumption that the sum of the individual effects is simply additive, the difference between the results of the simulation with concurrent drivers and the simulation results of the two or three individual drivers should be zero. To determine this interactive effect size, *d*, we added the relative control value (always 1 because results are standardized to the control) to the result of the scenario run with two or three drivers acting concurrently, and subtracted the individual effect sizes of the drivers according to:

$$d_{1,2,}={\text{Y}}_{\text{AB}}+{\text{Y}}_{\text{control}}–{\text{Y}}_{\text{A}}–{\text{Y}}_{\text{B}}$$

$$d_{1,2,3}={\text{Y}}_{\text{ABC}}+2{\text{Y}}_{\text{control}}–{\text{Y}}_{\text{A}}–{\text{Y}}_{\text{B}}–{\text{Y}}_{\text{C}}$$

where Y_AB_ is the value of the ecosystem metric resulting from having two interactive drivers acting concurrently and Y_ABC_ is the ecosystem metric resulting from three interacting drivers. A, B, and C are the three drivers (climate change, LBSP, and present day fishery). Y_control_ is the relative control value (with the coefficient 2 to ensure that the expected interaction *d* is 0 if effects are simply additive), resulting from a simulation of the control run, and Y_A_, Y_B_, and Y_C_ indicate the value of the simulation of just the one driver.

## Results

### Model validation

**Fig 2:**
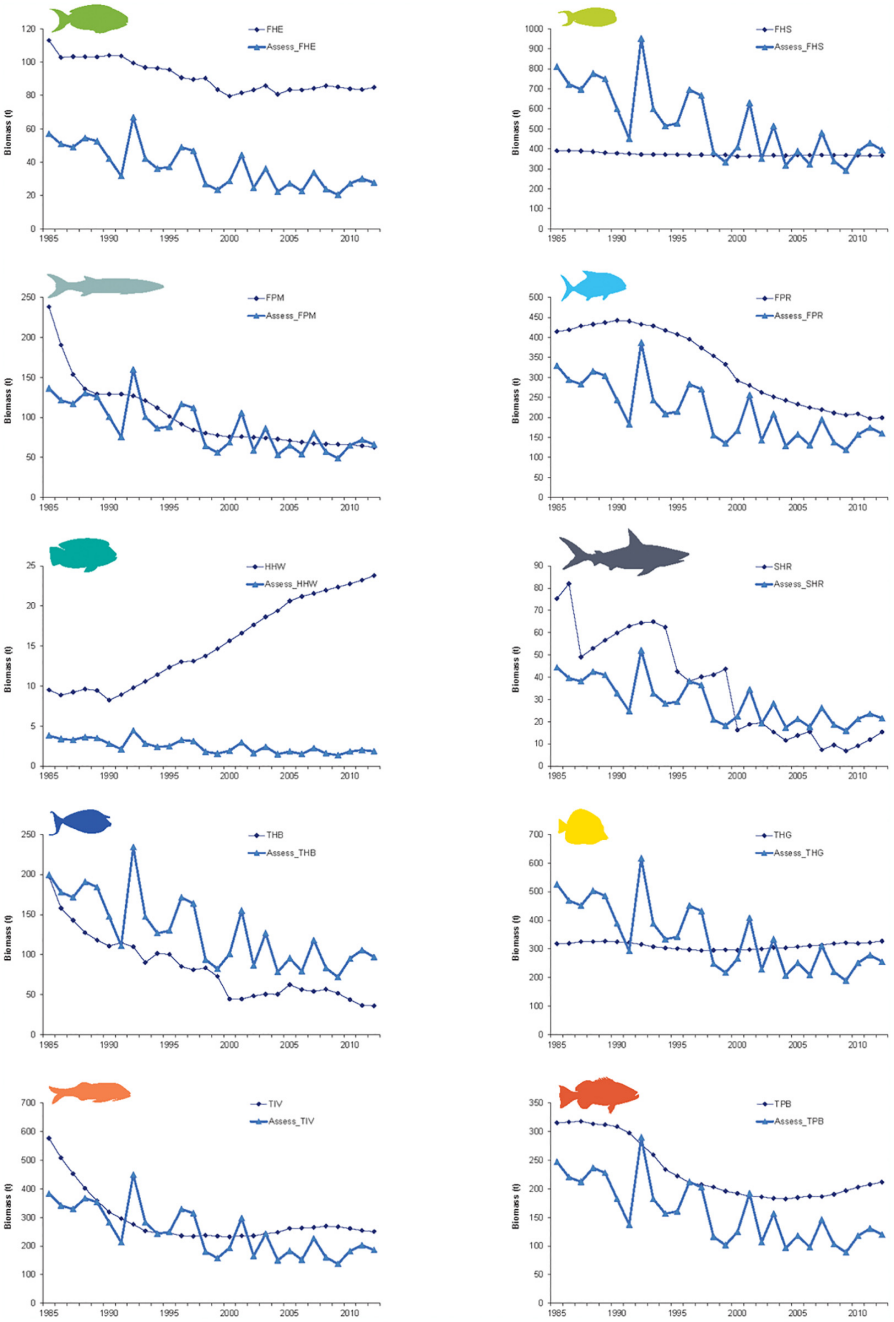
Atlantis biomass trajectories (in diamonds) compared to reconstructed time series (in triangles) of functional species groups exploited by fishers in Guam. Reconstructed biomass time series from Weijerman et al [46].

In the control simulation (scenario with no disturbances) fish biomass reached a level between that seen in the marine reserves in Guam and in unfished areas around the Northern Mariana Islands and invertebrates reached a stable biomass (Fig A2 in S1 Text). Atlantis also tracks individual vertebrate functional groups over time and weight-at-age remained stable and age-class size showed an expected distribution during a 50-year simulation (Figs A3 and A4 in S1 Text). As explained in detail in S1 Text, after adjusting the fishery catch data for assumed underreporting in the fishery data, the modeled biomass trend of most functional species groups corresponded well with the trend in the reconstructed biomass (Fig 2).

In general, the pattern of modeled coral biomass trajectories agreed with the expectation from empirical studies on the effects of climate change on corals (Fig A12 in S1 Text) and the effect of LBSP on the coral, algae and suspended solids trajectories (Fig A14 in S1 Text). These results led us to conclude that the model can adequately reproduce biomass trajectories after disturbances (e.g., effects of climate change and sediment and nutrient inputs) giving us confidence in the model validity (S1 Text).

### Individual drivers

**Fig 3:**
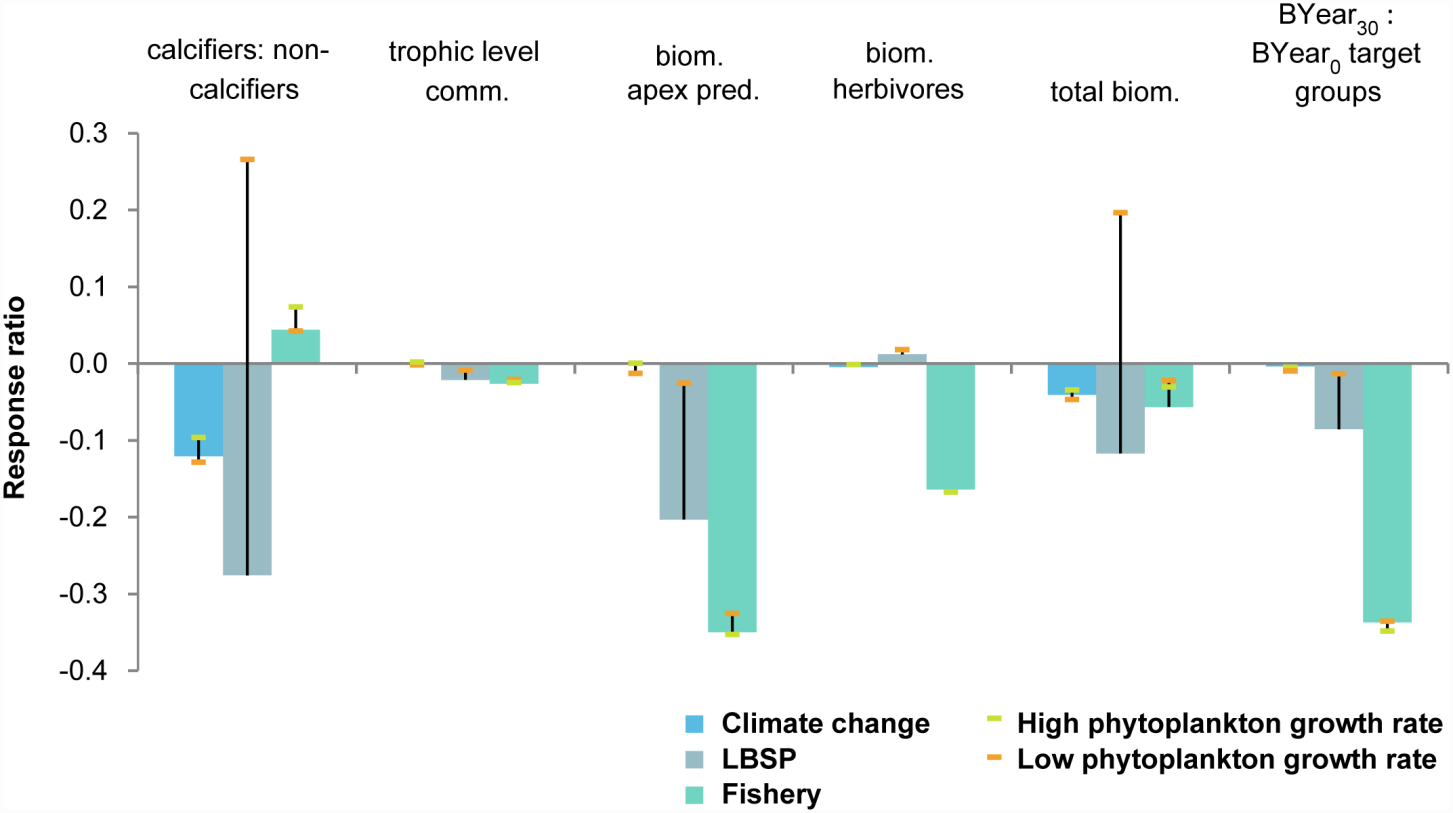
Standardized response ratio of individual drivers’ effect to control effect of the six ecosystem metrics after 30-year simulations with uncertainty stemming from uncertainty in phytoplankton growth rates indicated by the bars. LBSP is land-based sources of pollution.

Among the three 30-year (1985–2015) single stressor scenarios, fishing clearly had the largest overall ecosystem impacts based on the performance metrics used, mostly due to the large negative effect on the biomass of apex predators and the start-to-end ratio of biomass of fish groups targeted by recreational fishers (Fig 3). Global climate change almost exclusively affected the ratio of calcifiers to non-calcifiers resulting in a shift from coral and crustose coralline algae to turf and macroalgae (Fig 3). LBSP had the largest effect on the ratio of calcifiers to non-calcifiers and clear effects on the biomass of apex predators and total biomass.

Out of the three tested stressors, the LBSP scenario was most sensitive to parameterization of phytoplankton productivity (Fig 3). For four of the ecosystem metrics, altering the productivity changed the magnitude, but not the direction, of the effect. For the other two ecosystem metrics, the ratio of calcifiers to non-calcifiers and total biomass, results in our ‘main’ simulation led to a negative effect while the simulation with low phytoplankton growth rates led to a positive effect. Under the lower growth rate of phytoplankton, corals and demersal zooplankton were able to grow faster (increase in biomass ranging from 1.8 for corals to 3.3 for demersal zooplankton) which resulted in the increased ratio of calcifiers to non-calcifiers, and led to more energy flow to higher trophic levels especially invertebrate feeders and piscivores, which in turn resulted in the higher overall biomass and therefore a positive effect.

The high productivity simulation in the LBSP scenario led to unstable model outcomes. When we coupled nutrient inputs with high productivity, the model became numerically unstable and stopped running after approximately 8000 days which suggests that the static system structure and parameterization used was insufficient to reflect potential system changes in strongly perturbed systems under such high phytoplankton growth rates. Therefore, in the further analysis no data were available for the high phytoplankton growth rate simulation for the LBSP scenario.

Trophic level was least affected of the ecosystem metrics, possibly because of the taxonomic resolution of the model. Target and non-target groups had similar trophic levels and the biomass of apex predators was low in all simulations (e.g., 14% of total fish biomass in control scenario) compared to the biomass of the other fish groups. Consequently, even the 35% reduction in apex predator biomass due to fishing, and 18% reduction due to land-based sources of pollution, did not have a great influence on the overall trophic level of the community.

**Fig 4:**
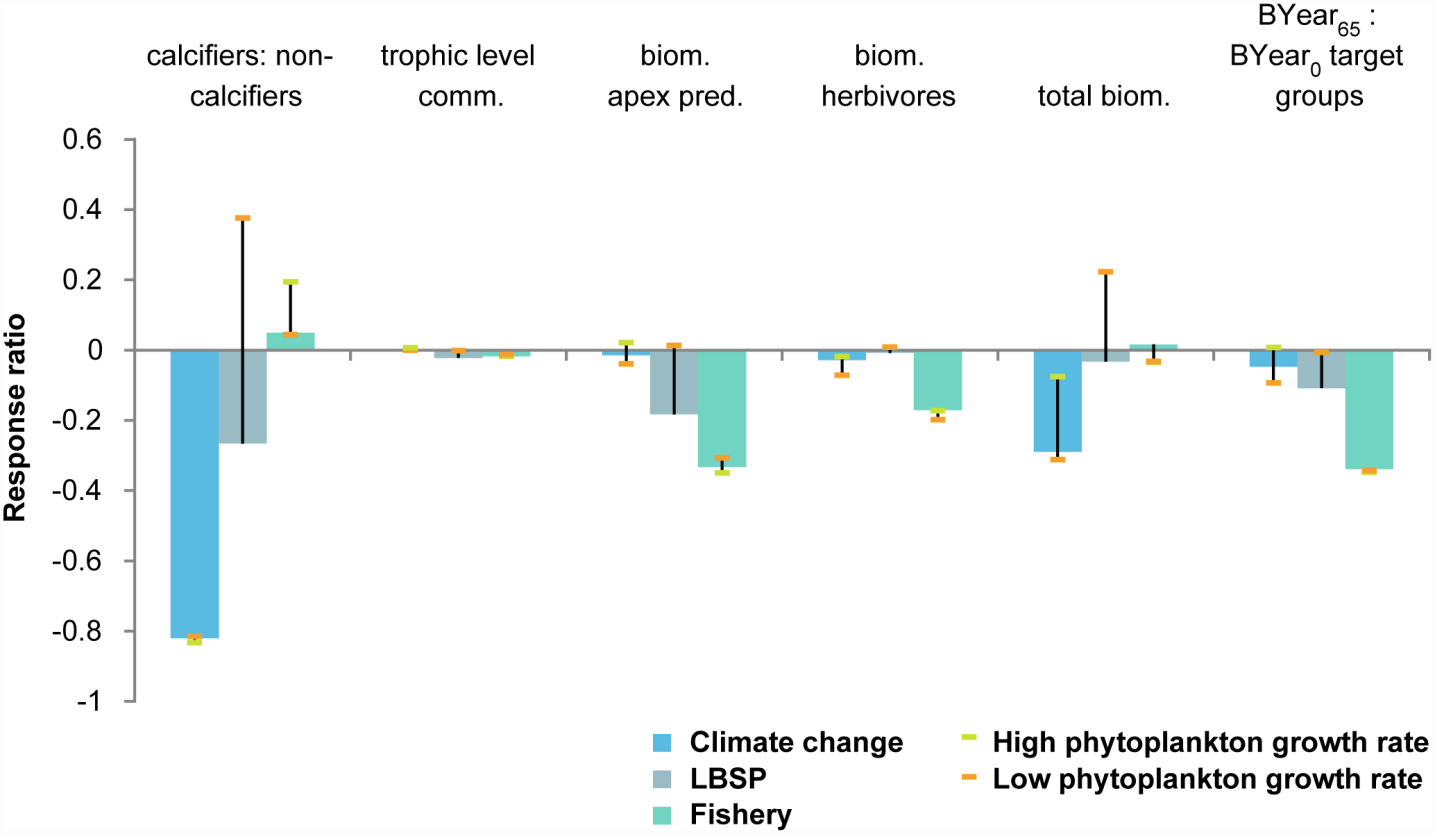
Standardized response ratio of individual drivers’ effect to control effect of the six ecosystem metrics after 65-year simulations with uncertainty stemming from uncertainty in phytoplankton growth rates indicated by the bars. LBSP is land-based sources of pollution.

**Fig 5:**
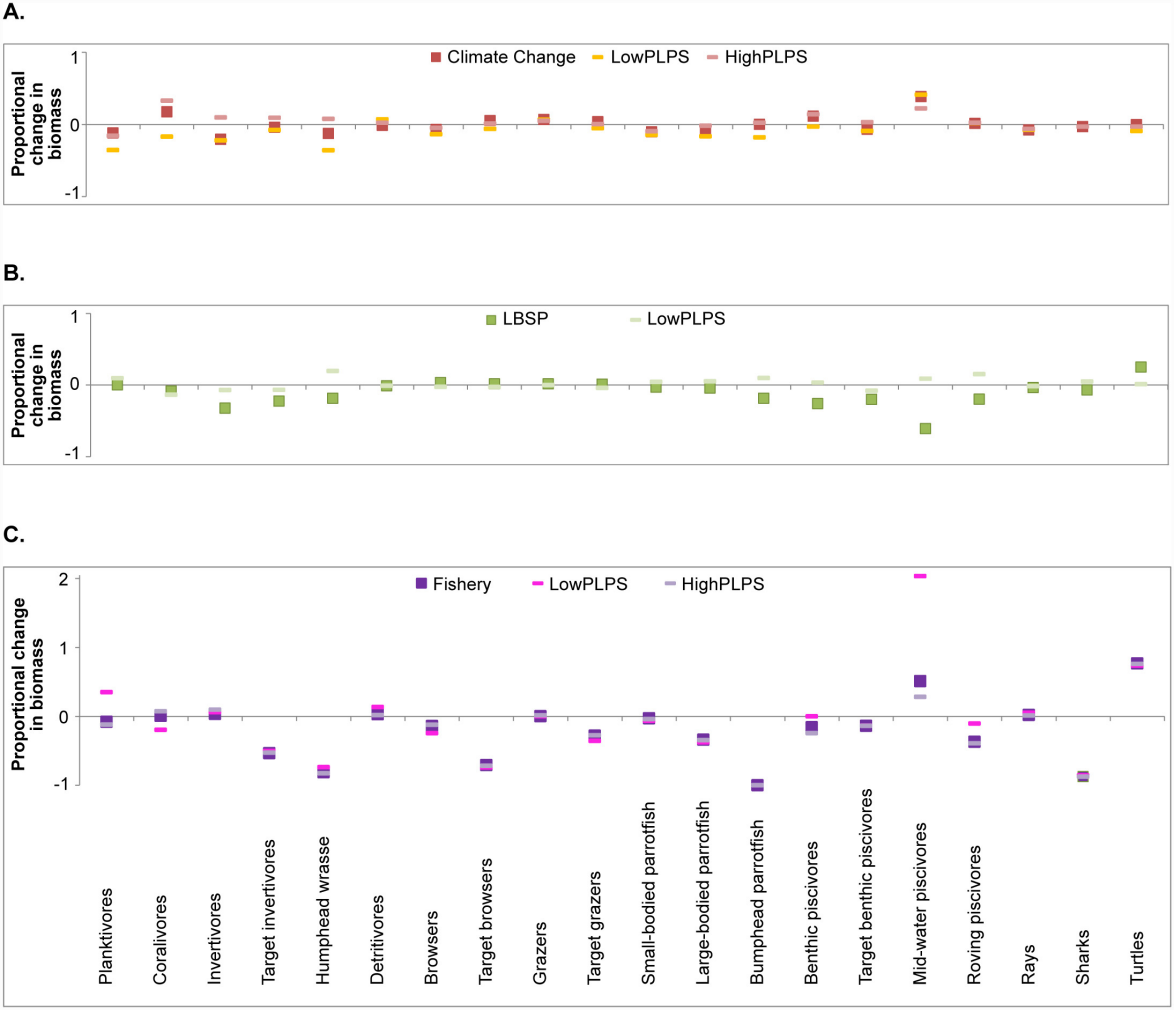
Vertebrate biomass response to each of the three simulated drivers, relative to the control biomass with uncertainty stemming from uncertainty in phytoplankton growth rates indicated by the bars. Each panel represents the biomass response to a driver: (A) Climate change, (B) Land-based sources of pollution (LBSP), (C) Fishing. Biomass responses are end values for year 2050 (65 year run). LowPLPS and HighPLPS represent results from low and high growth rates of phytoplankton parameterizations respectively.

Climate change effects dominate model dynamics at longer time scales (65 year simulations, 1985–2050). Using projected sea surface temperature rises, the bleaching threshold would be exceeded every year from 2023 onwards (S1 Text, year 48 in Fig A12). Therefore, it is not surprising that the effects of climate change are extremely high for the calcifiers to non-calcifiers ratio (ratio = 0.24 in climate change scenario versus 1.33 in control). In this longer simulation, climate change also had a negative effect on total biomass (Fig 4), including a decrease in most prey species other than some of the herbivorous fishes (Fig 5). Similar to the 30-year simulation, fishing affected almost all ecosystem metrics negatively, but particularly the biomass of apex predators and target fishes (Fig 4). The results for the LBSP scenario were again highly sensitive to assumptions about primary productivity. For instance, coral biomass was 80% higher for both 30 and 65 year simulations of low phytoplankton growth compared to the ‘main’ simulation of the LBSP scenario leading to the high uncertainty in the ratio of calcifiers (including corals) to non-calcifiers.

Sensitivity to parameterization of phytoplankton growth rate was highly dependent on the driver being tested (Fig 5). For the climate change scenario biomass of 12 vertebrate groups showed no sensitivity to the assumptions about primary productivity. For the remaining eight vertebrate groups, results of the main simulation was in general higher compared to the low phytoplankton growth rate simulation and lower compared to the high phytoplankton growth rate simulation (Fig 5A). For the LBSP scenarios, under a low-phytoplankton growth rate simulation, the biomass of nine vertebrate functional groups was higher compared to the main simulation (Fig 5B). For the fishing scenario assumptions about primary productivity had little effect on 16 of the 20 vertebrate groups, but the biomass of mid-water piscivores (in particular) and, to a lesser extent, the biomass of planktivores and roving piscivores were higher under the low phytoplankton growth rate simulation (Fig 5C). Overall, among the modeled vertebrate groups, turtles and mid-water piscivores came out as ‘winners’, as their predators, sharks, declined, and, in the case of turtles, competition for food (algae) also declined due to the decline in herbivore biomass under the fishing scenario (Fig 5).

**Fig 6:**
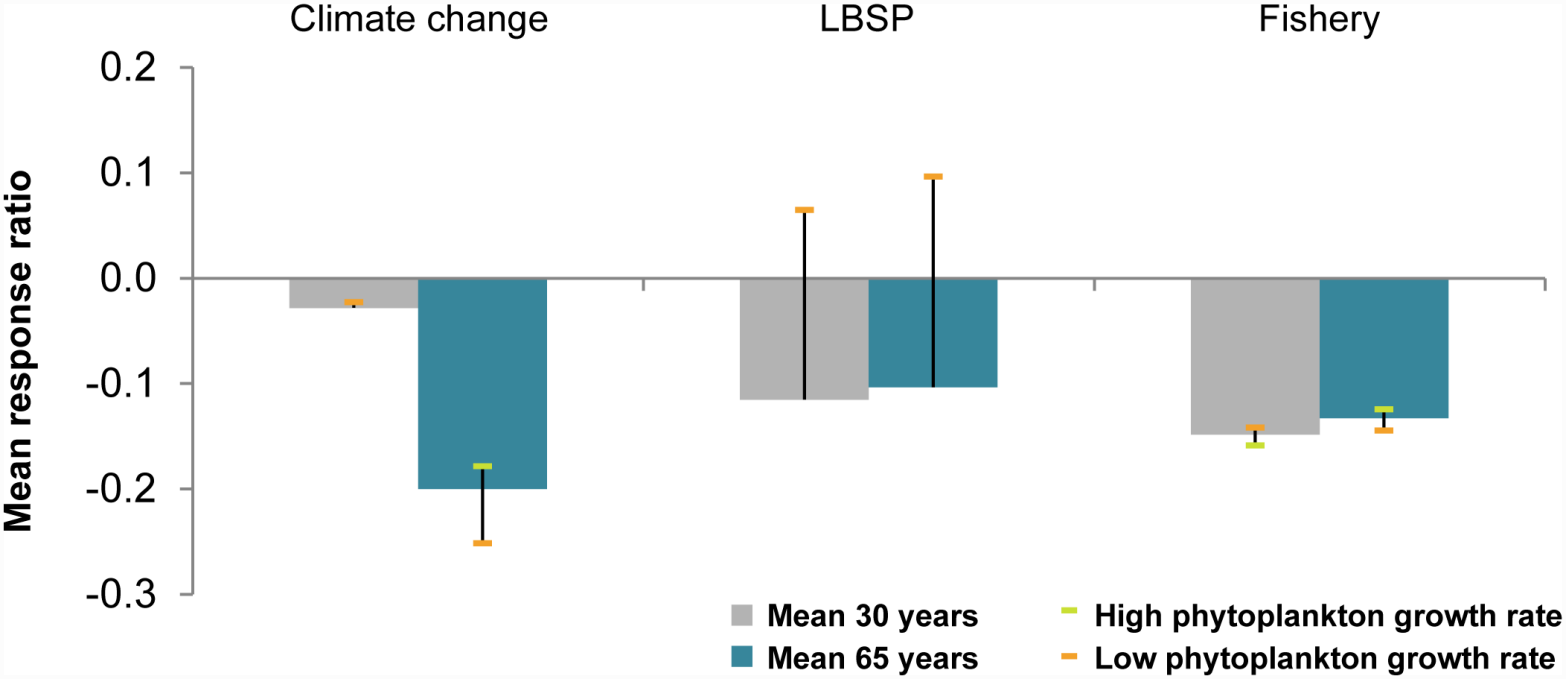
Average response ratio across all six ecosystem metrics of climate change, land-based sources of pollution (LBSP), and fishing after 30 (grey columns) and 65 (blue columns) years with uncertainty stemming from uncertainty in phytoplankton growth rates indicated by the bars.

Averaging all metrics (Fig 6) emphasizes that climate change is the dominant driver over the long term (65-year projections through year 2050), while fishing and LBSP appear to have influenced the performance of the system (as measured by our ecosystem metrics) most in the last 30 years (to 2015). Highest uncertainty in model outcome among the three drivers was for the LBSP simulations. In the main simulation mean effects on ecosystem metrics were negative, but under low plankton growth rates simulations ecosystem metrics responded positively to additional nutrients, especially the ratio of calcifiers to non-calcifiers and total biomass.

### Concurrent drivers

**Fig 7:**
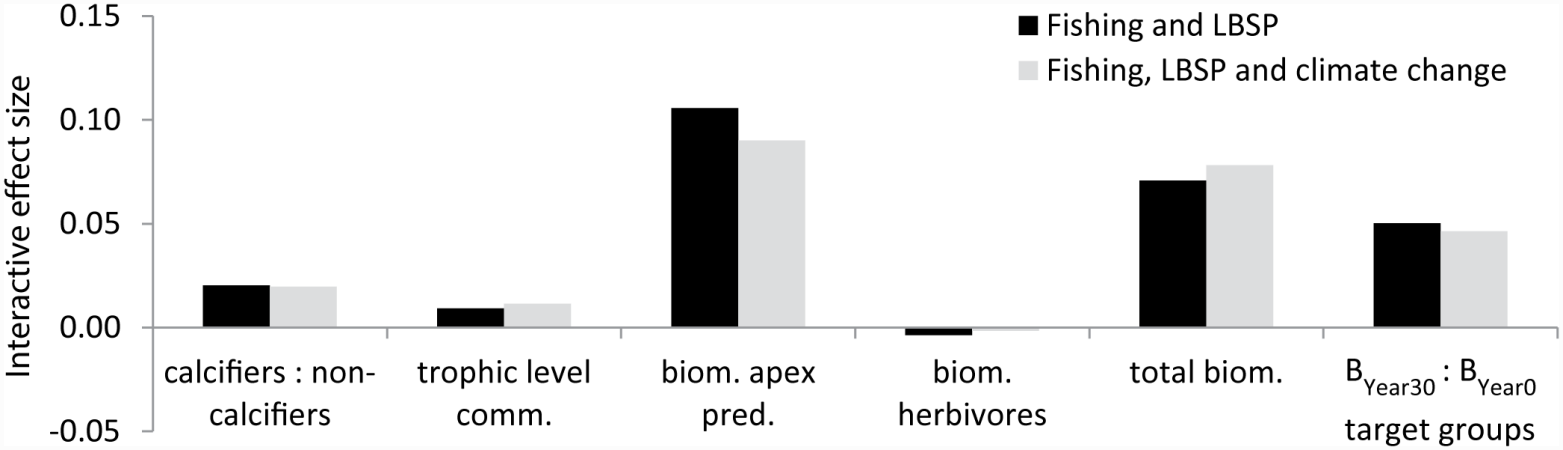
Interactive effect size on six ecosystem metrics (x-axis) in 2015. Results are based on simulations of fishing and LBSP concurrently and all three drivers (also including climate change) at the end of a 30-year run. The difference between the expected effect size, if these were simply additive, and the actual effect size of simulations with the two or three drivers concurrently is indicated by the black and grey bars respectively.

For the present-day conditions (30-year simulation), fishing appeared to have had the greatest impact closely followed by LBSP (Fig 6). Simulating those two drivers concurrently showed slight positive interactions for five out of six ecosystem metrics, i.e., the combined effects of concurrently simulating the two drivers were more positive than the additive effects of the two individual drivers. That was also true in simulations of all three drivers concurrently (Fig 7), but in both cases these antagonistic effects were ≤ 0.1. Note that strong declines in the ecosystem metrics were still observed in scenarios with concurrent drivers, for instance the biomass of apex predators declined to only 55% of the value under the control scenario.

**Fig 8:**
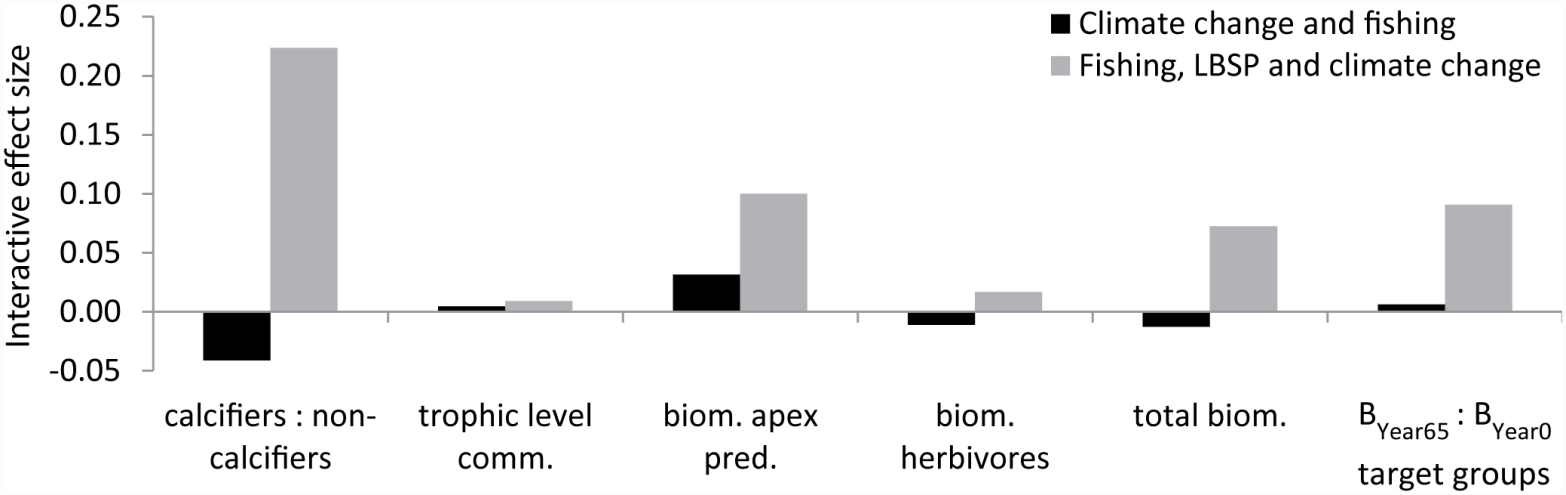
Interactive effect size on six ecosystem metrics (x-axis) by mid-century. Results based on simulations of climate change and fishery concurrently, and all three drivers at the end of a 65-year simulation. The difference between the expected effect size, if these were simply additive, and the actual effect size of simulations with the two or three drivers is indicated by the black and grey bars respectively.

For the 65-year simulation, climate change had the largest individual effect followed by fishing and LBSP (Fig 6), so we simulated those two drivers concurrently first, and then simulated all three drivers concurrently. Just as in the 30-year simulation, the interactive effect size was mostly positive, especially when all three drivers acted concurrently, i.e., a scenario with concurrent drivers led to slightly higher values of ecosystem metrics than could have been predicted from simply adding the individual driver effects (Fig 8). As with the 30-year simulation, despite the slight positive interactions of the concurrent drivers, actual effects were negative, just not as extreme as might be expected by summing the individual driver effects. For instance, despite the 0.21 positive interaction of the ratio of calcifiers to non-calcifiers (Fig 8), the combination of three drivers drove this ratio to only 19% of the control scenario result; the ratio of calcifiers to non-calcifiers was 0.24 if just climate change was simulated and 1.40 if just fishing was simulated, suggesting that the benefits of fishing for this metric were not fully realized when combined with climate change. Note also, that in the fishing simulations we did assume a constant fishing effort at the 2011 levels. If fishing pressure (e.g. form shore-based anglers) were to increase into the future then these results would need to be interpreted as minimal and the actual effects might be larger.

**Fig 9:**
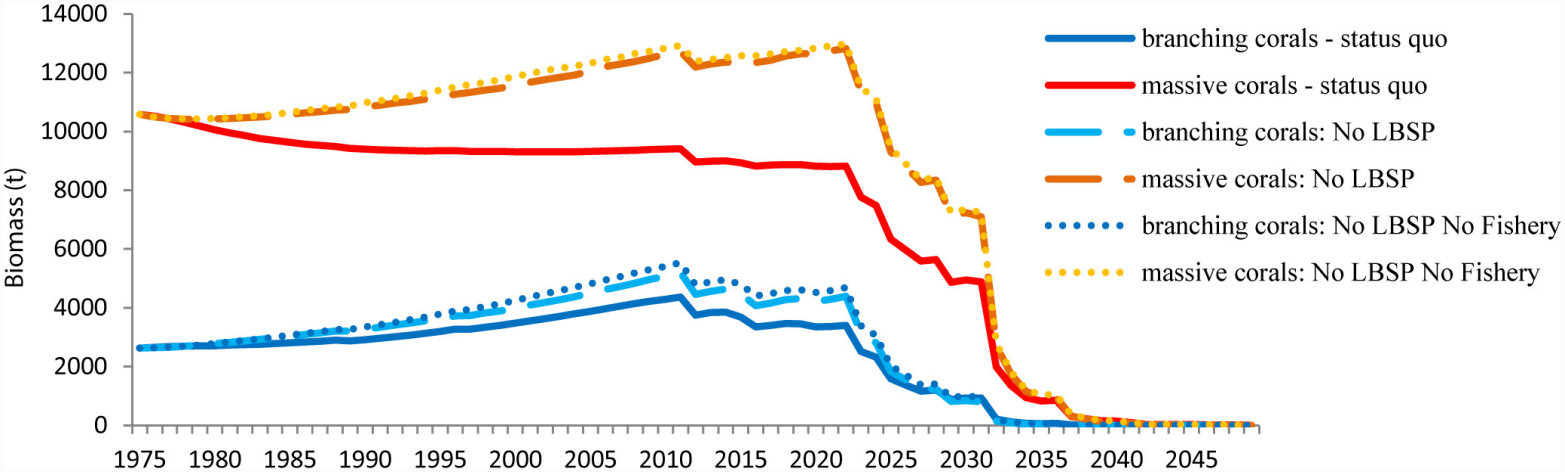
Projected effects of predicted climate changes concurrent with local threats (LBSP and fishing) on massive and branching coral biomass. The result of a no fishing scenario corresponded with the status quo scenario for both coral groups and was left out for clarity. Climate change predictions came from IPCC AR5 RCP8.5 scenario for pCO_2_ emissions.

In terms of management applications of these model results, reducing LBSP appears to have a noticeable effect on coral biomass, giving corals some additional capacity to deal with the early effects of climate change under the RCP8.5 scenario for CO_2_ emissions. However, when ocean temperature exceeds the bleaching threshold every year, the prognosis for corals is bleak (Fig 9). Our study indicates that when just fishing is restricted, coral biomass follows the same trend as under a status quo scenario (Fig 9).

**Fig 10:**
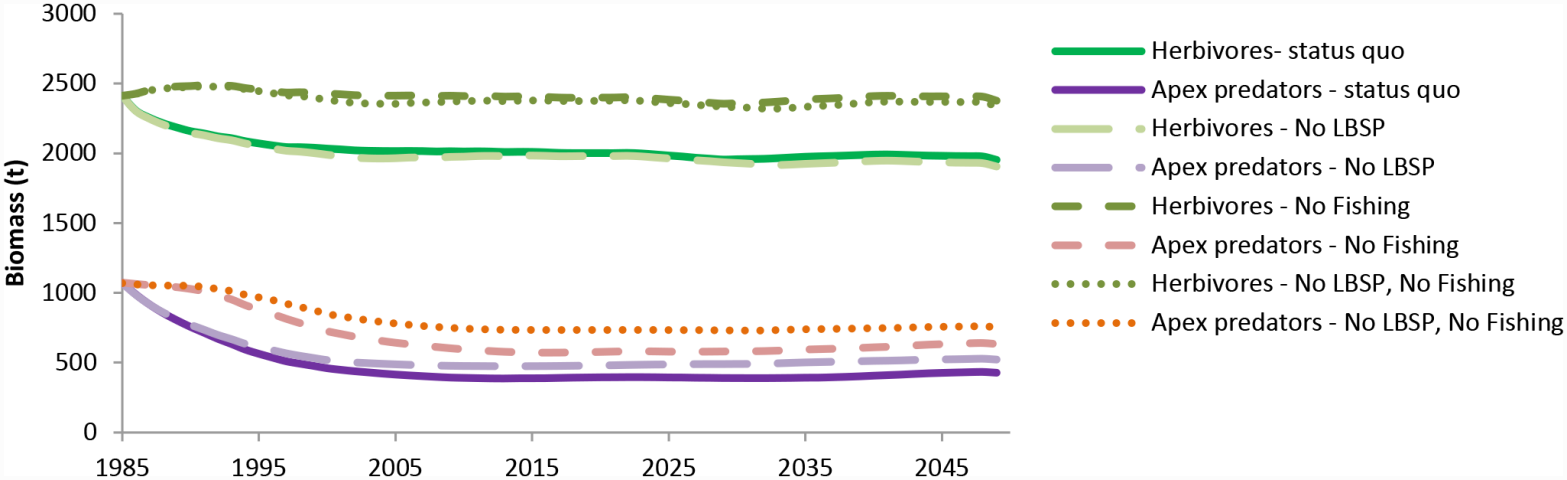
Projected effects of predicted climate changes concurrent with local threats (LBSP and fishing) on biomass of herbivorous fish and apex predators. Climate change predictions came from IPCC AR5 RCP8.5 scenario for pCO_2_ emissions.

For the same time frame, the trend for the biomass of apex predators and of herbivores (Fig 10), showed, not surprisingly, that the no-fishing scenarios (short dashed and dotted lines in Fig 10) resulted in the highest fish biomass. Similar results were obtained for the relative change in biomass of target species compared to the initial biomass: a declining and stabilizing trend at around 53% of initial biomass for status quo and at 55% for no LBSP scenarios and stabilizing at 80% for no fishing and at 85% for no fishing and no LBSP scenarios. What was surprising though, is that the herbivorous fish biomass stayed stable despite the reduction in coral biomass (and hence structural complexity and hiding places) in the last 15 years possibly because there was more food available (Fig 10).

However, a 110-year simulation (only simulated for the status-quo scenario) does indicate that the biomass of the herbivorous prey fish slowly declines after coral cover has declined with a lag-time of about 15 years (. These 15 years correspond well to their mean generational age-span of 12.3 (SD 6.4) years. This result could indicate that habitat is a more important factor in the life history of herbivorous fishes than food availability. Main ‘winners’ projected by the model at the end of this century under a status-quo scenario with climate change were turtles (100% increase), benthic filter feeders (600% increase) and detritivorous invertebrates (300% increase).

## Discussion

Ecosystem-based management considers the indirect and cumulative effects of multiple threats to a system. Ecosystems can frequently recover from short-term low-intensity disturbances, but when disturbances occur too frequently or when multiple disturbances impact the system in a short time span (i.e., before the system is recovered), recovery is limited or may not happen at all [11, 68, 69]. Quantifying the interactive effect size of disturbances is one way to gain insight into how these disturbances cumulatively affect the ecosystem. Looking first at the disturbances individually, climate change, including ocean warming and acidification, had the largest effect on the ecosystem compared to LBSP and fishing, with fishing being a close second when comparing ecosystem metrics at the end of 65-year simulations. Whereas climate change primarily affected the benthic reef community, fishing impacted all ecosystem metrics, and four out of six metrics negatively. Fishing had the strongest negative effect on the biomass of target species and apex predators. This result is expected from any selective fishery [70, 71]. However, contrary to what has been observed on Caribbean reefs [72, 73] fishing actually had a beneficial effect on the ratio of calcifiers to non-calcifiers. This increased ratio was due to an increase in coral biomass and a decrease in algal biomass. This positive effect of fishing on the ratio of calcifiers to non-calcifiers can be partly explained by the projected increase in turtle biomass, which were less preyed upon as the shark (main predator) populations had declined, and by less bio-erosion, especially from large parrotfishes as their biomass declined as well (Fig 5C). Although in terms of relative weight, in our results changes in coral biomass could be driven more directly by bottom-up forces (nutrients, sediments) and less so by top-down (grazing) forces.

Uncertainty stemming from sensitivity to primary productivity was most notable in the effects of LBSP on ecosystem metrics, especially on the ratio of calcifiers to non-calcifiers and total biomass. Recently the role of phytoplankton in structuring fish communities has received more attention and helped explain the large regional differences in fish biomass between reefs close to populated and unpopulated areas [74], corroborating the importance of obtaining good estimates of these phytoplankton groups.

Results also suggest that presently (end of 30-year simulation for the 1985 to 2014 period) fishing affects almost all ecosystem metrics negatively and that LBSP exacerbates this effect for all metrics, but not quite as badly as would be expected under additive assumptions. In the case of biomass of herbivores, fishing had a negative effect, but LBSP had a slight positive effect. The scenario with those concurrent drivers led to a slightly lower value of that ecosystem metric than could have been predicted from simply adding the individual driver effects. This result is likely because the input of nutrients and sediments, which led to an increase in food abundance, offset the reduction in biomass of herbivores through their extraction due to fishing. This pattern also held up after concurrently simulating the third driver, climate change. Despite the low interactive value, the combination of two and all three drivers drove the herbivore biomass to 84% of the value under the control scenario.

Cumulative effects of combining all three drivers in the 65-year simulation were negative. This corresponds with temperate fisheries systems where the ecosystem was worse off once all three drivers came into play [75, 76]. Estimated interactions were slightly positive, meaning that the combined effects were only slightly less than a null assumption of summing the individual effects of each of the three drivers.

In correspondence with the suggestions from some studies that by mitigating local stressors to reefs the coral’s resilience to climate change increases [3, 11, 29, 30], we found that when LBSP were stopped, coral biomass increased and stayed higher longer compared to the status quo scenario. Based on the uncertainty around the LBSP simulations, a lower phytoplankton growth rate could lead to an even higher biomass of corals, corroborating the importance of reducing LBSP. However, in contrast to the general idea that a fishing moratorium could mitigate the decline in coral biomass, our simulations suggests that this increasing trend in coral biomass was only slightly improved when fishing was also stopped (Fig 9). This result was also seen in a study in the Indian Ocean where fishery closures did not change hard coral cover [16]. Though studies in the Caribbean [11, 77] showed that high herbivore populations are important for the recovery of coral populations in some situations, our results do not suggest that this provides a substantial buffer against climate change for coral cover. Indeed, a study in the Great Barrier Reef [78] and a global meta-analysis [79] showed a similar demise of coral cover when bleaching events were taken into account despite the establishment of MPAs. In the Caribbean study, depending on the location, a high presence of herbivores did postpone the most detrimental effects of ocean warming on coral cover by 18–50 years [11]. This discrepancy in the influence of herbivores could be caused by the way herbivore populations were modelled. In the Edwards et al. [11] model, grazing intensity was fixed at 40% in the high grazing scenario and contrasted with a 10% grazing term in the low grazing scenario. In our model, although the total biomass of herbivores was lower in the status quo scenario, the grazing intensity appeared to still be high enough to keep the biomass of macroalgae down allowing corals to cope with their competitive pressure and remain at higher biomasses. Model skill results (S1 Text) showed that the model over-estimates some herbivorous fish groups, most notably the small-bodied parrotfish. This over-estimation of small-bodied parrotfish may contribute to our projection of the relatively weak fishing effects on coral biomass.

Our results indicate that water quality is a key local threat in the decline of coral biomass. Improving the water quality might delay the coral’s ultimate climate-driven decline by 5 to 8 years or more if phytoplankton growth rate is low. This improvement could buy corals time to acclimate to higher temperatures [80]. A study on the coral genus *Pocillopora* from Guam suggested that corals can, at least in part, acclimate to temperatures of 32°C which would be sufficient for persistence under the RCP8.5 scenario [81]. If corals cannot acclimate over such short time spans (5–8 years) and we experience the RCP8.5 pathway, our model suggests that reef-building corals will be severely impacted by 2035–2040 (Fig 9). This result is similar to a more general modeling result from Pacific reefs where coral cover dropped to 5% by the year 2050 and close to zero in the year 2055 [82]. Ortiz et al. [82] suggested that corals in the Pacific could recover if we can reduce to the RCP 2.6 low CO_2_ emission scenario.

With the reduction of the structural framework of a degraded coral reef, after a time lag of about 15 years, the biomass of herbivorous fish declined. This time lag is in between those observed in the western Indian Ocean (5–10 years [83]) and the Caribbean (25–30 years [84]). In general, roving herbivores, such as surgeonfishes and parrotfishes, can increase in abundance after mass bleaching events [27], but their recruits are dependent on coral habitat and are responsible for the lag-time effect in the ultimate decline in population size [83]. Indirect effects of coral loss also include an abundance of sponges [85] and a decrease in reef fish, mostly obligate corallivorous species, pomacentridae and gobies [86]. Our study shows that benthic filter feeders (including sponges) had doubled in biomass whereas sea stars (including the corallivorous crown-of-thorns seastars) declined. Corallivorous fish did not decline but, in fact, increased. This increase is likely because the modeled corallivorous fish functional group included species that also feed on soft coral and sponges. Similarly, the functional group planktivores (including pomacentrids) were not restricted to the small planktivores that are always associated with (mostly branching) corals, but also included large-bodied species, for example, unicorn fishes, resulting in less of a decline that would be expected from truly reef dependent species [27, 87].

### Concluding remarks

We successfully developed an integrated coral reef ecosystem model that takes into account the key reef dynamics and their relationship to disturbances. The adapted Guam Atlantis model met the three main criteria for model development and is stable with plausible biomass trajectories. Furthermore, model simulations of these dynamics corresponded well with empirical data from around Guam and regional studies. The model would, however, still benefit from further refinement. In particular, fishery data, and the nutrient, phytoplankton, and zooplankton dynamics could be improved, as should the handling of vertical mixing. Besides those improvements, alternative predictions of pH and aragonite saturation could more realistically project changes to calcifying organisms, including corals. Nevertheless, we are confident that the current model still provides a good indication of the relative short- and long-term importance of the different drivers examined.

Quantifying an ecosystem models’ uncertainty and performing skill assessments are still an under-studied part of ecosystem modeling, partly because of the complexity (and hence long run times and large number of parameters) of ecosystem models that computationally prohibits the use of well-established statistical analyses. Naturally, however, those results would greatly enhance the models’ robustness and management applicability. By providing insights within a consistent setting, this version of the Guam Atlantis model can be used as a decision-support tool to quantify the relative trade-offs of alternative ecosystem-based management scenarios. Guam Atlantis is capable of simulating the consequences of different management strategies (e.g., reduction in land-based sources of pollution or fishing), while simultaneously allowing for the expected effects of ocean warming and acidification, and therefore has utility for a range of regional (e.g., regulating pollution, land use and fisheries) and global (e.g., world-wide mitigations of CO_2_ and other greenhouse gasses) management applications.

Simulating the main stressors on coral reef ecosystems suggests that the reefs around Guam are presently predominantly affected by fishing and secondarily by the input of nutrients and sediments although there is considerable uncertainty regarding the results of LBSP scenarios depending on the growth rate used in the model for phytoplankton. In the near future (20–30 years from now), the predicted climate change will have the most profound effect on coral reefs. Reducing additional nutrients and sediments could mitigate the loss of coral biomass for at least 5 to 8 years, but once the temperature exceeds the bleaching threshold annually, corals are unlikely to survive. A consequence of the loss of corals is the slow decline in fish abundance, particularly of those that use the corals as habitat during a part of their life cycle, and this decline could impact the reef-fish fishery negatively.

## Supporting Information

S1 Table Functional groups of the Guam Atlantis Model. (DOCX)

S2 Table Coral related parameters used in Guam Atlantis. (DOCX)

S3 Table Guam Atlantis model output data. (XLSX)

S1 Text Detailed information on the development and validation of the Guam Atlantis Ecosystem Model. (DOCX)

S2 Text Equations for vertebrate movement. (DOCX)
