# Supplementary material for: An Integrated Coral Reef Ecosystem Model to Support Resource Management under a Changing Climate
Source: PLoS One. 2015 Dec 16;10(12):e0144165. doi: 10.1371/journal.pone.0144165 (PMC4682628; doi:10.1371/journal.pone.0144165)
Supplement: S1 Table — (DOCX) [file pone.0144165.s001.docx]

# **S1 Table. Functional groups of the Guam Atlantis Model.**

The table shows marine vertebrate species per functional group, their abundance from 2011 surveys conducted by NOAA-PIFSC Coral Reef Ecosystem Division, and their life history characteristics. k = growth constant in the Von Bertalanffy growth curve, Linf = infinite length, t_max_ = maximum age in years, L-W is the length-weight relationship with the constants a and b, and recruit age means recruitment from pelagic stage to the reef in days (equivalent to Pelagic Larval Duration). The values are the weighted means of all the species making up the functional group. For values per species (see Weijerman et al 2014). Additionally, invertebrate groups with descriptions are given.

| **Group** | **Code** | **Numbers per m2** | | **Mortality per y** | **k** | **Linf cm** | | **t_max_ y** | **L-W a** | | **L-W b** | | **age at maturity Y** | **Recruit age d** |
| --- | --- | --- | --- | --- | --- | --- | --- | --- | --- | --- | --- | --- | --- | --- |
| 1. Planktivores | FPL | 0.04 | | 1.24 | 0.58 | 25.32 | | 6.70 | 0.02 | | 3.05 | | 1.39 | 23 |
| 2. Coralivores | FCO | 0.00 | | 1.71 | 0.98 | 18.56 | | 4.37 | 0.05 | | 2.95 | | 1.17 | 37 |
| 3. Invertivores | FIV | 0.01 | | 1.01 | 0.57 | 25.37 | | 7.51 | 0.03 | | 3.07 | | 1.38 | 49 |
| 4. Target Invertivores | TIV | 1.59 | | 0.77 | 0.38 | 39.53 | | 9.38 | 0.02 | | 2.99 | | 2.21 | 27 |
| 5. Humphead wrasse | HHW | 0.00 | | 0.20 | 0.10 | 232.44 | | 30.00 | 0.01 | | 3.14 | | 5.90 | 34 |
| 6. Detritivores | FDE | 0.02 | | 1.41 | 0.84 | 29.05 | | 17.41 | 0.02 | | 3.05 | | 0.92 | 55 |
| 7. Browsers | FHB | 0.00 | | 2.14 | 1.43 | 29.82 | | 4.35 | 0.01 | | 3.16 | |  | 15 |
| 8. Target Browsers | THB | 0.00 | | 1.25 | 0.68 | 36.27 | | 14.18 | 0.03 | | 3.09 | | 2.58 | 70 |
| 9. Grazers | FHG | 0.04 | | 1.21 | 0.55 | 21.95 | | 6.56 | 0.02 | | 3.18 | | 1.63 | 60 |
| 10. Target Grazers | THG | 0.02 | | 1.42 | 0.80 | 25.36 | | 24.00 | 0.03 | | 2.99 | | 1.37 | 32 |
| 11. Scrapers | FHS | 0.01 | | 0.73 | 0.92 | 22.78 | | 8.71 | 0.02 | | 3.05 | | 1.4 | 35 |
| 12. Excavators | FHE | 0.00 | | 1.08 | 0.65 | 44.69 | | 11.14 | 0.02 | | 3.08 | | 2.19 | 35 |
| 13. Bumphead parrotfish | BHP | 0.00 | | 0.14 | 0.14 | 133.1 | | 33.00 | 0.02 | | 3.04 | | 8.00 | 35 |
| 14. Benthic piscivores | FPB | 0.02 | | 0.53 | 0.21 | 72.11 | | 8.00 | 0.01 | | 3.03 | | 2.00 | 60 |
| 15. Target Benthis piscivores | TPB | 0.00 | | 0.50 | 0.24 | 53.01 | | 15.9 | 0.02 | | 2.99 | | 3.79 | 60 |
| 16. Mid-water piscivores | FPM | 0.01 | | 0.58 | 0.32 | 72.86 | | 8.96 | 0.01 | | 3.00 | | 1.99 | 60 |
| 17. Roving piscivores | FPR | 0.03 | | 0.22 | 0.10 | 170.51 | | 28.27 | 0.01 | | 3.00 | | 5.59 | 120 |
| 18. Reef-associated sharks | SHR | 0.00 | | 0.25 | 0.16 | 276.73 | | 22.26 | 0.00 | | 3.47 | | 4.25 | 360 |
| 19. Rays | RAY | 0.00 | | 0.17 | 0.09 | 271.37 | | 23.18 | 0.01 | | 3.20 | | 5.42 | 60 |
| 20. Sea Turtles | REP | | 0.00 | 0.14 | 0.09 | 108.90 | 62.00 | | | 0.05 | 3.30 | 37.50 | | 2190 |

**INVERTEBRATES**

| **#** | | **Group** | **Code** | | **Description** |
| --- | --- | --- | --- | --- | --- |
| 21 | Benthic Carnivores | | | BC | carnivorous epifauna invertebrates |
| 22 | Benthic Detritivores | | | BD | sea cucumbers, lobster, polycheates, detritivorous gastropods and crusteaceans (e.g.,crabs) |
| 23 | Benthic Meiofauna | | | BO | infauna: herbivorous polychaetes, gastropods and crustaceans |
| 24 | Benthic Suspension Feeders | | | BFF | octocoral, sponges, tunicates, zooanthids, giant clams, bivalves, polychaetes, foraminifera, bryzoans, brittle stars |
| 25 | Sheltering corals | | | CRS | corals species with a branching/tabular/columnar morphology |
| 26 | Non-sheltering corals | | | CRN | coral species with a massive/encrusting morphology |
| 27 | Cephalopods | | | CEP | octopus, squids |
| 28 | Benthic Grazers | | | BG | urchins (helmet, collectors, pencil, boring urchin, diadema) |
| 29 | Sea Stars | | | BSS | including crown-of-thorns seastar |
|  | **ALGAE** | | |  |  |
| 30 | Turf algae | | | TRF | < 1cm |
| 31 | Macroalgae | | | MA | > 1cm |
| 32 | Crustose-coralline algae | | | CCA |  |
|  | **PLANKTON** | | | |  |
| 33 | Small phytoplankton | | | PS | picoeukaryotes, cyanobacteria, < 1µm |
| 34 | Large phytoplankton | | | PL | > 1µm including diatoms |
| 35 | Zooplankton -carnivores | | | ZC | chaetognath, amphipods, crab larvae, isopods, mysid shrimps, polychaetes |
| 36 | Demersal zooplankton | | | ZD | pelagic fish & invert larvae, copepods, polychaetes, foraminiferas |
| 37 | Zooplankton - herbivores | | | ZH | Copepods |
|  | **BACTERIA** | | |  |  |
| 38 | Pelagic Bacteria | | | PB | hetrotrophic bacteria (0.2-1 µm) |
| 39 | Benthic bacteria | | | BB | hetrotrophic bacteria (0.2-1 µm) |
|  | **DETRITUS** | | |  |  |
| 40 | carrion | | | DC | newly dead – in fishing discards |
| 41 | refractory detritus | | | DR | turnover time in order of years |
| 42 | Labile detritus | | | DL | easily degraded, turnover time in order of months |
