# Supplementary material for: An Integrated Coral Reef Ecosystem Model to Support Resource Management under a Changing Climate
Source: PLoS One. 2015 Dec 16;10(12):e0144165. doi: 10.1371/journal.pone.0144165 (PMC4682628; doi:10.1371/journal.pone.0144165)
Supplement: S2 Table — (DOCX) [file pone.0144165.s002.docx]

# S2 Table. Coral related parameters used in Guam Atlantis.

| **Parameter** | **value** | **mini-mum** | **maxi-mum** | **unit** | **source** |
| --- | --- | --- | --- | --- | --- |
|  |  |  |  |  |  |
| ***Growth related:*** |  |  |  |  |  |
| *Autotrophic feeding* |  |  |  |  |  |
| light threshold for feeding | 700 |  |  | W/m^2^ | assuming 15% of noon sunlight |
| corals feeding during day | 20 |  |  | % |  |
| translocation of nutrients to host | 90 |  |  | % | [[1](#_ENREF_1)] |
| *Sediment smothering effects* |  |  |  |  |  |
| Ksmother coefftient; | 0.054 |  |  | nd | [[2](#_ENREF_2)] |
| Ksmother_constant; | 0.4622 |  |  | nd | [[2](#_ENREF_2)] |
| *Algal-coral competition* |  |  |  |  |  |
| max growth rate massive corals | 0.0003 | 0.00011 | 0.019 | d^-1^ | 0.003 [[3](#_ENREF_3)]; 0.003 [[4](#_ENREF_4)]; 0.0004-0.0194 [[5](#_ENREF_5)]; 0.00011-0.0055 [[6](#_ENREF_6)] |
| max growth rate branching corals | 0.0003 | 0.00011 | 0.059 | d^-1^ |  |
| max growth rate turf | 0.075 | 0.027 | 0.4 | d^-1^ | 0.029 [[7](#_ENREF_7)]; 0.027 [[8](#_ENREF_8)]; 0.04 [[5](#_ENREF_5)]; 0.05-0.4 [[6](#_ENREF_6)] |
| max growth rate macroalgae | 0.018 | 0.014 | 0.4 | d^-1^ | 0.018 [[7](#_ENREF_7)]; 0.06 [[9](#_ENREF_9)] (*Kappaphycus*); 0.014 [[5](#_ENREF_5)]; 0.05-0.4 [[6](#_ENREF_6)] |
| max growth rate CCA | 0.01 | 0.01 | 0.026 | d^-1^ | 0.010 [[7](#_ENREF_7), [10](#_ENREF_10)]; 0.026 [[5](#_ENREF_5)] |
| max growth rate small phytoplankton | 0.41 | 0.41 | 3.2 | d^-1^ | 0.41 [[4](#_ENREF_4)]; 3.2 [[11](#_ENREF_11)] |
| max growth rate large phytoplankton | 4.1 | 0.41 | 6.4 | d^-1^ | 0.41 [[4](#_ENREF_4)]; 6.4 [[11](#_ENREF_11)] |
| turf-coral overgrow (rate of growth suppression) | 0 |  |  |  | [[12](#_ENREF_12)] |
| macroalgae-coral overgrow (rate of growth suppression) | 0.0010 | 0.00014 | 0.0011 | d^-1^ | [[6](#_ENREF_6), [12-14](#_ENREF_12)] |
| CCA-coral overgrow (rate of growth suppression) | 0 |  |  |  | [[15](#_ENREF_15)] |
| turf-coral facilitation (% facilitation of coral recruitment) | 5 | 5 | 15 | % | [[6](#_ENREF_6), [12](#_ENREF_12)] |
| macroalgae-coral competition (% growth inhibition) | 80 | 40 | 90 | % | [[6](#_ENREF_6), [12](#_ENREF_12)] |
| CCA-coral facilitation (% facilitation of coral recruitment) | 5 | 5 | 15 | % | [[6](#_ENREF_6), [12](#_ENREF_12)] |
| half saturation constant for growth on DIN for massive corals | 50 | 5.6 | 28 | mgN/m^3^ | 0.23-0.8 µM NO3 [[16](#_ENREF_16)]; 5-22µM NO3 [[17](#_ENREF_17)] |
| half saturation constant for growth on DIN for branching corals | 50 | 5.6 | 28 | mgN/m^3^ | 5.6-28 (0.4-2.0 µM); NO3 0.23-0.8 µM [[16](#_ENREF_16)]; 5-22uM [[17](#_ENREF_17)] |
| half saturation constant for growth on DIN for turf algae | 6 |  |  | mgN/m^3^ |  |
| half saturation constant for growth on DIN for macroalgae | 6 | 1.4 | 11.2 | mgN/m^3^ | 0.1-0.8 mmol/m^3^ [[18](#_ENREF_18)] |
| half saturation constant for growth on DIN for CCA | 6 |  |  | mgN/m^3^ |  |
| half saturation constant for growth on DIN for small phytoplankton | 0.35 | 0.0084 | 1.4 | mgN/m^3^ | 0.025 mmol/m^3^ [[19](#_ENREF_19)]; 0.6 nmol/m3 [[11](#_ENREF_11)]; 0.1 mmol/m^3^ [[18](#_ENREF_18)] |
| half saturation constant for growth on DIN for large phytoplankton | 0.35 | 0.0028 | 1.4 | mgN/m^3^ | 0.025 mmol/m^3^ [[19](#_ENREF_19)]; 0.2 nmol/m^3^ at [[11](#_ENREF_11)]; 0.1 mmol/m^3^ [[18](#_ENREF_18)] |
| light saturation branching corals | 35 | 11 | 67 | W/m^2^ | 50-300 µE/m^2^/s [[20](#_ENREF_20)] |
| light saturation massive corals | 35 | 11 | 67 | W/m^2^ | 50-300 µE/m^2^/s [[20](#_ENREF_20)] |
| light saturation turf | 5 |  |  | W/m^2^ |  |
| light saturation macroalgae | 5 |  |  | W/m^2^ | Skagerrak&Baltic Sea ~ 100 µmol photons/m^2^/s= 0.135 W/m^2^ |
| light saturation CCA | 5 |  |  | W/m^2^ |  |
| light saturation small phytoplankton | 20 |  |  | W/m^2^ | [[19](#_ENREF_19)] |
| light saturation large phytoplankton | 20 |  |  | W/m^2^ | [[19](#_ENREF_19)] |
|  |  |  |  |  |  |
| ***Rugosity related:*** |  |  |  |  |  |
| rugosity based habitat dependency coefficient | 1.4613 |  |  | nd | Shape of relationship inferred from [[21-23](#_ENREF_21)] |
| rugosity based habitat dependency constant | 0.0475 |  |  | nd |  |
| rugosity based habitat dependency cap | 4 |  |  | nd |  |
| rugosity based habitat dependency scalar | 8 |  |  | nd |  |
|  |  |  |  |  |  |
| rugosity constant | 1.3 |  |  | nd | adapted from [[24](#_ENREF_24)] |
| massive corals colony height parameter | 0.715 |  |  | cm |  |
| branching corals colony height parameter | 0.81 |  |  | cm | average of slopes from *Porites* sp. [[24](#_ENREF_24)] |
| branching corals max. colony diameter | 60 | 0-5 | >300 | cm | mean 26 cm L. Raymundo, unpublished data, 17 cm CRED data |
| massive corals max. colony diameter | 30 | 0-5 | >300 | cm | mean 14 cm L. Raymundo, unpublished data, 15 cm CRED data |
| **Ocean change related** |  |  |  |  |  |
| *Aragonite saturation - CO_3_ calculation* | |  |  |  |  |
| Karag_A | 0.9485 |  |  | nd | [[25](#_ENREF_25)]; Values for coefficients from proxy fitting exercise - from data given in Bjerrum plot |
| Karag_B | 8.20416 |  |  | nd | [[25](#_ENREF_25)]; Values for coefficients from proxy fitting exercise - from data given in Bjerrum plot |
| Karag_C | 2.3641 |  |  | nd | [[25](#_ENREF_25)]; Values for coefficients from proxy fitting exercise - from data given in Bjerrum plot |
| Karag_D | 8 |  |  | nd | [[25](#_ENREF_25)]; Values for coefficients from proxy fitting exercise - from data given in Bjerrum plot |
| Karag_pH; | time series pCO_2_ |  |  | nd | IPCC AR5 RC8.5; for control pCO_2_ 3580 constant |
| Kca_const; | 0.0103 |  |  |  | [[25-28](#_ENREF_25)] |
| K_Ks; | 6 10^-9^ |  |  | nd | [[26](#_ENREF_26)] |
| *Calcification related parameters* |  |  |  |  |  |
| Reference baseline calcification rate | 15.03 | 12.03 | 17.03 | ppm | 380 ppm [[29](#_ENREF_29), [30](#_ENREF_30)] (needed to lower to get realistic rates of calcification) |
| calcification T constant | 9.7 | 9.03 | 10.37 | nd | [[30](#_ENREF_30), [31](#_ENREF_31)] |
| calcification T coefficient | 18.83 | 12.98 | 24.68 | nd | [[30](#_ENREF_30), [31](#_ENREF_31)] |
| calcification optimum temperature | 29 |  |  | °C | close to natural summer ambient temperature [[32](#_ENREF_32)]; summer solstice temp [[30](#_ENREF_30)] |
| calcification Lambda | 0.42 | 0.33 | 0.51 | nd | [[30](#_ENREF_30), [31](#_ENREF_31)] |
| light threshold for autotrophic feeding | 700 |  |  | W/m^2^ | assumption 85% of noon irradiance |
| proportion feeding during light | 20 |  |  | % | assuming mostly feeding at night |
| host remineralization | 90 |  |  | % | [[1](#_ENREF_1)] |
| *Growth/fecundity relationship with pH* | |  |  |  |  |
| CCA coeff. & const | -3.0, 0.5 |  |  | nd | [[33](#_ENREF_33), [34](#_ENREF_34)] |
| large phytoplankton parameters | 1.4, 1.1705, 4, 150 |  |  | nd | [[35](#_ENREF_35), [36](#_ENREF_36)] |
| small phytoplankton parameters | 3.7, 1.0, 5, 200 |  |  | nd | [[35](#_ENREF_35), [36](#_ENREF_36)] |
| macroalgae parameters | 1.6, 1.1,  6, 150 |  |  | nd | [[35](#_ENREF_35), [36](#_ENREF_36)] |
| zooplankton parameters | 5.0, 1.7 |  |  | nd | [[35](#_ENREF_35), [37](#_ENREF_37), [38](#_ENREF_38)] |
| benthic grazers (urchins) coeff. & const | -3.0, 0.5 |  |  | nd | [[35](#_ENREF_35), [37](#_ENREF_37), [38](#_ENREF_38)] |
| benthic filter feeders (bivlaves) coeff. & const | -3.0, 0.5 |  |  | nd | [[38](#_ENREF_38)] |
| *Bleaching parameters* |  |  |  |  |  |
| bleaching mortality massive corals | 22.5 | 3.14 | 41.87 | % | [[39-42](#_ENREF_39)] |
| bleaching mortality branching corals | 42 | 19.7 | 100 | % | [[39-42](#_ENREF_39)] |
| bleaching rate massive corals) | 25 | 9.8 | 40.2 | % | [[39-42](#_ENREF_39)] |
| bleaching rate branching corals | 24.2 | 6.9 | 41.5 | % | [[39-42](#_ENREF_39)] |
| bleaching recovery rate massive corals | 0.0027 |  |  | d^-1^ | [[43](#_ENREF_43)] |
| bleaching recovery rate branching corals | 0.0822 |  |  | d^-1^ | [[44](#_ENREF_44)] (0.2 degree /decade) |
| bleaching temperature massive corals/branching corals | 30.4 |  |  | °C | 1 degrees above summer max ambient, NOAA Coral Reef Watch |
| prop zooxanthellae massive corals/branching corals | 30 | 5 | 30 | % | 15% [[45](#_ENREF_45)]; <5% [[46](#_ENREF_46)]; 30% [[1](#_ENREF_1)] |
| degree heating weeks (DHW) threshold massive corals | 4 |  |  | DHW | [[41](#_ENREF_41), [42](#_ENREF_42)] |
| degree heating weeks (DHW) threshold branching corals | 3 |  |  | DHW | [[41](#_ENREF_41), [42](#_ENREF_42)] |

**References**

1. Gustafsson M. Modelling cnidarian-algae symbiosis, implications of nutrient supply, temperature, and light intensity. Sydney: university of Technology; 2013.

2. Wolanski E, Richmond RH, Davis G, Bonito V. Water and fine sediment dynamics in transient river plumes in a small, reef-fringed bay, Guam. Estuarine, coastal and shelf science. 2003;56(5–6):1029-40. doi: 10.1016/s0272-7714(02)00321-9.

3. Mumby P. The impact of exploiting grazers (Scaridae) on the dynamics of Caribbean coral reefs. Ecological Applications. 2006;16(2):747-69.

4. Weijerman M, Fulton EA, Parrish FA. Comparison of coral reef ecosystems along a fishing pressure gradient. PLoS ONE. 2013;8(5):e63797. doi: 10.1371/journal.pone.0063797.

5. Ruiz Sebastián C, McClanahan TR. Description and validation of production processes in the coral reef ecosystem model CAFFEE (Coral–Algae–Fish-Fisheries Ecosystem Energetics) with a fisheries closure and climatic disturbance. Ecological Modelling. 2013;263. doi: 10.1016/j.ecolmodel.2013.05.012.

6. Melbourne-Thomas J, Johnson C, Fulton E. Regional-scale scenario analysis for the Meso-American Reef system: Modelling coral reef futures under multiple stressors. Ecological Modelling. 2011;222(10):1756-70.

7. Klumpp D, McKinnon A. Community structure, biomass and productivity of epilithic algal communities on the Great Barrier Reef: Dynamics at different spatial scales. Marine ecology progress series Oldendorf. 1992;86(1):77-89.

8. Miller RJ, Reed DC, Brzezinski MA. Community structure and productivity of subtidal turf and foliose algal assemblages. Marine Ecology Progress Series. 2009;388:1-11.

9. Dailer ML, Smith JE, Smith CM. Responses of bloom forming and non-bloom forming macroalgae to nutrient enrichment in Hawai ‘i, USA. Harmful Algae. 2012;17:111-25.

10. Weijerman M, Brown V. A summary of the Guam Coral Reef Ecosystem Model workshop and discussions in Guam, November 14–20, 2012. Admin. Rep. H-13-3: Pacific Islands Fisheries Science Center, NMFS, NOAA, 2013.

11. Wang X, Behrenfeld M, Le Borgne R, Murtugudde R, Boss E. Regulation of phytoplankton carbon to chlorophyll ratio by light, nutrients and temperature in the Equatorial Pacific Ocean: a basin-scale model. Biogeosciences. 2008;5:3869-903.

12. McCook, Jompa, Diaz P. Competition between corals and algae on coral reefs: a review of evidence and mechanisms. Coral Reefs. 2001;19(4):400-17. doi: 10.1007/s003380000129.

13. Mumby PJ, Dytham C. Metapopulation dynamics of hard corals. In: Kritzer JP, Sale PF, editors. Marine metapopulations. Amsterdam: Elsevier Academic Press; 2005. p. 157-203.

14. Lirman D. A simulation model of the population dynamics of the branching coral Acropora palmata Effects of storm intensity and frequency. Ecological Modelling. 2003;161(3):169-82. doi: <http://dx.doi.org/10.1016/S0304-3800(02)00346-0>.

15. McCook L. Competition between corals and algal turfs along a gradient of terrestrial influence in the nearshore central Great Barrier Reef. Coral Reefs. 2001;19(4):419-25.

16. Domotor SL, D'Elia CF. Nutrient uptake kinetics and growth of zooxanthellae maintained in laboratory culture. Marine Biology. 1984;80(1):93-101. doi: 10.1007/bf00393132.

17. D'Elia CF, Domotor SL, Webb KL. Nutrient uptake kinetics of freshly isolated zooxanthellae. Marine Biology. 1983;75(2-3):157-67. doi: 10.1007/bf00405998.

18. Lapointe BE. Nutrient thresholds for bottom-up control of macroalgal blooms and coral reefs. Limnol Oceanogr. 1997;44:1586-92.

19. Huisman J, Thi NNP, Karl DM, Sommeijer B. Reduced mixing generates oscillations and chaos in the oceanic deep chlorophyll maximum. Nature. 2006;439(7074):322-5.

20. Kleypas JA. Modeled estimates of global reef habitat and carbonate production since the last glacial maximum. Paleoceanography. 1997;12(4):533-45. PubMed PMID: ISI:A1997XP46200003.

21. DeMartini E, Jokiel P, Beets J, Stender Y, Storlazzi C, Minton D, et al. Terrigenous sediment impact on coral recruitment and growth affects the use of coral habitat by recruit parrotfishes (F. Scaridae). Journal of Coastal Conservation. 2013;17:1-13.

22. Graham NAJ, Wilson SK, Jennings S, Polunin NVC, Bijoux JP, Robinson J. Dynamic fragility of oceanic coral reef ecosystems. Proceedings of the National Academy of Sciences of the United States of America. 2006;103(22):8425-9. doi: 10.1073/pnas.0600693103. PubMed PMID: ISI:000238206800024.

23. Friedlander AM, Parrish JD. Habitat characteristics affecting fish assemblages on a Hawaiian coral reef. Journal of Experimental Marine Biology and Ecology. 1998;224(1):1-30. doi: <http://dx.doi.org/10.1016/S0022-0981(97)00164-0>.

24. Bozec YM, Alvarez‐Filip L, Mumby PJ. The dynamics of architectural complexity on coral reefs under climate change. Global Change Biology. 2014.

25. Zeebe RE, Wolf-Gladrow DA. Carbon Dioxide, Dissolved (Ocean). Encyclopedia of Paleoclimatology and Ancient Environments: Springer; 2009. p. 123-7.

26. Kleypas JA, Feely RA, Fabry VJ, Langdon C, Sabine CL, Robbins LL. Impacts of ocean acidification on coral reefs and other marine calcifiers: A guide for future research. Report of a workshop held 18-20 April 2005, St. Petersburg, FL, sponsored by NSF, NOAA, and the US Geological Survey: 2006.

27. Edmond JM, Gieskes J. On the calculation of the degree of saturation of sea water with respect to calcium carbonate under< i> in situ</i> conditions. Geochimica Et Cosmochimica Acta. 1970;34(12):1261-91.

28. Berner RA. Activity coefficients of bicarbonate, carbonate and calcium ions in sea water. Geochimica Et Cosmochimica Acta. 1965;29(8):947-65.

29. Anthony KRN, Maynard JA, Diaz-Pulido G, Mumby PJ, Marshall PA, Cao L, et al. Ocean acidification and warming will lower coral reef resilience. Global Change Biology. 2011;17(5):1798-808. doi: 10.1111/j.1365-2486.2010.02364.x.

30. Silverman J, Lazar B, Cao L, Caldeira K, Erez J. Coral reefs may start dissolving when atmospheric CO2 doubles. Geophysical Research Letters. 2009;36(5):L05606. doi: 10.1029/2008GL036282.

31. Anthony K, A Kleypas J, Gattuso JP. Coral reefs modify their seawater carbon chemistry–implications for impacts of ocean acidification. Global Change Biology. 2011;17(12):3655-66.

32. Jokiel P, Coles S. Effects of temperature on the mortality and growth of Hawaiian reef corals. Marine Biology. 1977;43(3):201-8.

33. Langdon C, editor Review of experimental evidence for effects of CO_2_ on calcification of reef builders. Proceedings of the 9th International Coral Reef Symposium; 2002; Bali, Indonesia.

34. Kroeker KJ, Kordas RL, Crim RN, Singh GG. Meta-analysis reveals negative yet variable effects of ocean acidification on marine organisms. Ecology Letters. 2010;13(11):1419-34.

35. Hendriks IE, Duarte CM, Álvarez M. Vulnerability of marine biodiversity to ocean acidification: a meta-analysis. Estuarine, coastal and shelf science. 2010;86(2):157-64.

36. Harvey BP, Gwynn-Jones D, Moore PJ. Meta-analysis reveals complex marine biological responses to the interactive effects of ocean acidification and warming. Ecology and evolution. 2013;3(4):1016-30. doi: 10.1002/ece3.516.

37. Kurihara H, Ishimatsu A. Effects of high CO< sub> 2</sub> seawater on the copepod (< i> Acartia tsuensis</i>) through all life stages and subsequent generations. Marine Pollution Bulletin. 2008;56(6):1086-90.

38. Wittmann AC, Pörtner H-O. Sensitivities of extant animal taxa to ocean acidification. Nature Climate Change. 2013;3(11):995-1001.

39. McClanahan TR. The relationship between bleaching and mortality of common corals. Marine Biology. 2004;144(6):1239-45.

40. Jokiel PL, Coles SL. Response of Hawaiian and other Indo-Pacific reef corals to elevated temperature. Coral Reefs. 1990;8(4):155-62.

41. Donner SD, Skirving WJ, Little CM, Oppenheimer M, Hoegh-Guldberg O. Global assessment of coral bleaching and required rates of adaptation under climate change. Global Change Biology. 2005;11:2251-65. doi: 10.1111/j.1365-2486.2005.01073.x.

42. McClanahan TR, Ateweberhan M, Graham NAJ, Wilson SK, Sebastian CR, Guillaume MMM, et al. Western Indian Ocean coral communities: bleaching responses and susceptibility to extinction. Marine Ecology Progress Series. 2007;337:1-13. PubMed PMID: ISI:000247105100001.

43. Lombardi MR, Lesser MP, Gorbunov MY. Fast repetition rate (FRR) fluorometry: variability of chlorophyll a fluorescence yields in colonies of the corals, Montastraea faveolata (w.) and Diploria labyrinthiformes (h.) recovering from bleaching. Journal of Experimental Marine Biology and Ecology. 2000;252(1):75-84. doi: <http://dx.doi.org/10.1016/S0022-0981(00)00238-0>.

44. Kvitt H, Rosenfeld H, Zandbank K, Tchernov D. Regulation of apoptotic pathways by *Stylophora pistillata* (Anthozoa, Pocilloporidae) to survive thermal stress and bleaching. PLoS ONE. 2011;6(12):e28665. doi: 10.1371/journal.pone.0028665.

45. Odum HT, Odum EP. Trophic structure and productivity of a windward coral reef community on Eniwetok Atoll. Ecological Monographs. 1955;25:291-320.

46. Thornhill DJ, Rotjan RD, Todd BD, Chilcoat GC, Iglesias-Prieto R, Kemp DW, et al. A Connection between Colony Biomass and Death in Caribbean Reef-Building Corals. PLoS ONE. 2011;6(12):e29535. doi: 10.1371/journal.pone.0029535.
