# Supplementary material for: An Integrated Coral Reef Ecosystem Model to Support Resource Management under a Changing Climate
Source: PLoS One. 2015 Dec 16;10(12):e0144165. doi: 10.1371/journal.pone.0144165 (PMC4682628; doi:10.1371/journal.pone.0144165)
Supplement: S1 Text — (DOCX) [file pone.0144165.s004.docx]

# **S1 Text. Detailed information on the development and validation of the Guam Atlantis Ecosystem Model**

**Table of Contents**

[Introduction](#h.5zolxnj1j383)

[Added coral code: Coral biomass](#h.qcu022f95fpj)

[Recruitment](#h.spxtx5j9rmpu)

[Mortality](#h.bb66dkti6kji)

[Model validation and verification](#h.cvv8q28sgvcd)

[Added coral code: Coral related dynamics](#h.5z9jjfpjnwde)

[Driver 1: Climate change](#h.uhnoxl1kvdqa)

[Climate change effects on coral reef ecosystems](#h.mmknoo5tocg4)

[Driver 2: Land-based sources of pollution](#h.lckic377mlph)

[Land-based sources of pollution: Sedimentation and nutrient elevation effects on benthic composition and target fish biomass](#h.bsfwh97a34uy)

[Driver 3: Fishing activity](#h.vyxmwp7xnrjz)

[Effects of fishing of herbivores on corals-algae-grazers dynamics](#h.yk8lpavi93i)

[Sensitivity and skill assessments](#h.cgt21hibcrx4)

[Conclusion](#h.nqfw5b1uo5be)

# Introduction

In previous applications of Atlantis models, corals, if included at all, were modeled as benthic filter feeders [[1-5](#_ENREF_1)]. We used that framework and parameterized it for the coral reef ecosystems around Guam. Details on the design, data sources and parameterization can be found in Weijerman et al. (2014). Here we describe the methods, assumptions, validation and verification of code added to Atlantis that represent the relationships of key coral and coral reef dynamics (Fig A1). We organized the text per research topic rather than the traditional methods - results - discussion format. First we discuss the added code that addresses the growth of corals (change in coral biomass) and assess the adapted model’s validation and verification process. We then verified these added dynamics by examining the model behavior over 30–75 years without any disturbances, i.e., a ‘control’ system, following the guidelines for Atlantis model development. Then we detail added code for key drivers, namely (1) climate change (ocean warming and acidification), (2) changes in land use (eutrophication and sedimentation) and (3) fishing activities. By including extensive empirical data collected from field studies in Guam, local dynamics are projected over time-scales of decades and trends that manifest themselves are identified. Model outcomes of scenarios with each of the disturbances were compared with empirical studies from Guam or with regional studies when local information was not available. Lastly we discuss how we conducted sensitivity analyses for the levels of primary productivity, rugosity (structural complexity) and the benthic algae-coral competition and present the results.

## Added coral code: Coral biomass

Recruitment is added as biomass to the youngest size class (Fig A1). Changes in coral biomass are modeled as the sum of growth (which includes recruitment) recruitment, minus mortality (including predation; Eq. 1). Predation relates to the abundance of other system components including corals themselves and species that eat coral (Fig A1). Each of these processes is modeled as a series of functions based on empirical relationships taken from published studies.

$\frac{d(Cx)}{dt}= \mu_{Cx} -M_{lin Cx}- M_{quad Cx}- \sum_{i=predator groups} P_{Cx,i}-F_{Cx}$ Eq. 1

where *Cx* is the invertebrate consumer (coral polyp), with *µ_Cx_* the coral specific growth, $M_{lin Cx}$ and *M_quad Cx_*  the linear and quadratic mortality of corals, *P_i_* is predation by group *i* and *F* is fishing or take of this group.


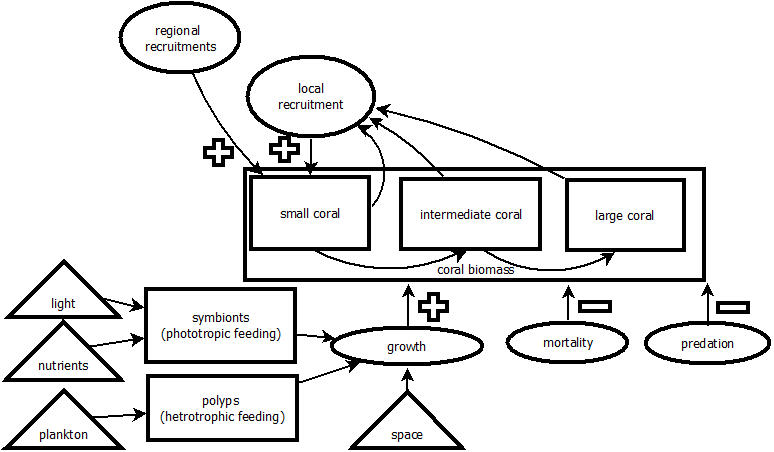


Fig A1. **Schematic representation of modeled changes in coral biomass due to growth (based on feeding), recruitment minus mortality and coral predation (including bio-erosion).**

Rectangles represent biomass, ovals processes and triangles environmental properties. Other environmental conditions that influence growth and mortality (such as elevated temperature, sediments, overgrowth by macroalgae), are left out for clarity.

Growth is influenced by competition for food, light, space and oxygen. Corals are heterotrophic at night and photosynthesize during daytime and excess fixed carbon is converted to lipids for reserves [[6](#_ENREF_6), [7](#_ENREF_7)]. Coral polyps can take up dissolved organic material for growth [[8](#_ENREF_8)]. At night, coral polyps extend their tentacles to capture zooplankton, particulate organic matter and bacteria [[9](#_ENREF_9), [10](#_ENREF_10)] that provides them with nitrogen and other nutrients. The coral code uses the standard Atlantis consumption routine with invertebrates modeled as biomass pools (Eqs. 1 and 2) and for vertebrates Atlantis tracks abundance, biomass, weight-at-age and condition (reserve weight versus structural weight) of each age class of each group through time. For coral polyps, change in biomass is modeled with:

$\mu C=\left[ \varepsilon_{Cx} \bullet\sum_{i=living prey} P_{i, Cx}+ \sum_{j=detritus} P_{j,Cx} \bullet\varepsilon_{Cx,j} \right] \bullet hSp \bullet hO2$ Eq. 2

where $\varepsilon$*_Cx_* is the growth efficiency of *Cx* when feeding on live prey, $\varepsilon$*_Cx,j_* the efficiency when feeding on detritus, *hSp* is space limitation and *hO_2_* is oxygen limitation. Space limitation is determined by *min(1.0,max(0.0,(1.0 - B / (SPmax* $\bullet$ *area_hab))))* with *B* the combined coral polyp and symbiont biomass, *SPmax* the maximum biomass per area and *area_hab* the species specific amount of habitat area available for settlement or growth.

The routine for primary producers was adjusted to allow for the symbionts-host representation (Eq. 3):

$\frac{\mu C}{dt} = Bsymbiont \bullet\mu C \bullet hN \bullet hI \bullet hSp$ Eq. 3

with *µ_C_* the coral specific growth , *Bsymbiont* is the symbiotic zooxanthellae biomass, *hN* is nutrient limitation determined by the coral specific half saturation constant for growth on DIN and available nutrients and *hI* is light limitation determined by the coral specific light saturation constant and the available light.

Coral growth is the sum of the heterotrophic and autotrophic growth. We further assumed that the photosynthesis threshold is 85% of noon daylight; proportion of corals feeding during daylight is 20%; translocation of energy from symbionts to host coral is 90% [[7](#_ENREF_7)]. Growth is also influenced by environmental factors (e.g., aragonite saturation state) and water quality (e.g., sediment load) as explained below in the section “Added coral code: Coral related dynamics”.

### Recruitment

Recruitment is estimated as a function of supply and settlement survivorship, with supply being driven by the net import/export from Guam, which we assume is relatively small, and local population fecundity which is affected by colony sizes and abundance with larger corals having a higher fecundity [[11](#_ENREF_11)] (Eq. 4). To account for the difference in fecundity between size classes of corals they were modeled as three size/age classes each with three sub-bins. Corals can skip across sub-bins within size class but they cannot skip the next size class. Demographic coral data is sparse so we assumed an initial uniform distribution of size classes. These transitions are modeled according to:

$T(i) = \frac{max\_accel\_transition}{1 +e^{(-3 \bullet(i -1.5))}}$ Eq. 4

where *T(i)=*Transition to next size class for size class *i*, growth restriction is capped by the max_accel_transition parameter (0.1) and follows an exponential curve to mimic their 3D surface area [[12](#_ENREF_12)].

Local recruitment is the sum of the recruitment of each size class according to:

$local R = 1- e^{-0.001*B}$ Eq. 5

with *R* = recruitment and *B* = coral biomass for each size class.

Most Pacific corals are spawners (Baird et al. 2009) and with an increasing distance between coral colonies, chances of fertilization reduce due to Alleé effect [[11](#_ENREF_11)]. Survival of larvae until settlement is low [[13](#_ENREF_13)] decreasing connectivity at regional scales [[11](#_ENREF_11)]. Settlement survivorship is modeled to be positively related to hard substrate, turf and crustose-coralline algae (CCA) and negatively to upright algae [[14](#_ENREF_14)]. As corals can only recruit to hard habitat, habitat is set as a potential facilitator for coral recruitment (Eq. 6). This relationship is a gross approximation and should ideally be replaced with a connectivity matrix if the appropriate data becomes available.

$total R = localR+regionalR*\left( CCA cover+hard substrate+\partial*Turf cover \right)$ Eq. 6

with regional recruitment modeled as a constant and ∂ a correction factor (see assumptions), CCA is crustose-coralline algae.

We assumed that all modeled corals are spawners; coral recruits are the expected number of surviving larvae given current environmental conditions, i.e., we do not model the pelagic larval stage; contribution to recruitment follows an exponential relationship with coral biomass (Eq. 5) [[11](#_ENREF_11)]; recruitment is zero in macroalgal habitats, but is facilitated by crustose coralline algae and hard substrate, and by turf algae with a correction factor of 0.05-0.15 [[15](#_ENREF_15)].

### Mortality

Mortality is influenced by environmental factors (such as elevated temperatures) and by predation and competition (density-dependency, macroalgal overgrowth, fish bites, crown-of-thorns seastar predation, micro-eroders). These processes are discussed below.

## Model validation and verification

Criteria used for testing the validity of the model and to verify model outcomes were based on guidelines for Atlantis model development [[3](#_ENREF_3), [4](#_ENREF_4), [16](#_ENREF_16)]:

1. Predicted biomass matches observations or are plausible based on information from domain experts. In this case for many benthic groups this defaults to staying within a factor of two of initial conditions. For fish groups we expected predicted biomass (with no fishing or other disturbances) to approximate those in marine reserves in Guam or from the unpopulated Northern Mariana Islands (NOAA Pacific Islands Fisheries Science Centre, Coral Reef Ecosystem Division (CRED) data);
2. Weight-at-age stays stable and abundance of size classes decreases with increasing size classes (few large organisms and many small ones); and
3. Reproduced catch data has a plausible trajectory and magnitude of historical change without pushing any modeled group to extinction.

In control simulations (scenario with no disturbances) fish biomass reached a level between that seen in the marine reserves in Guam and in unfished areas around the Northern Mariana Islands and invertebrates reached a stable biomass (Fig A2) thereby complying with the first criterion for model development.


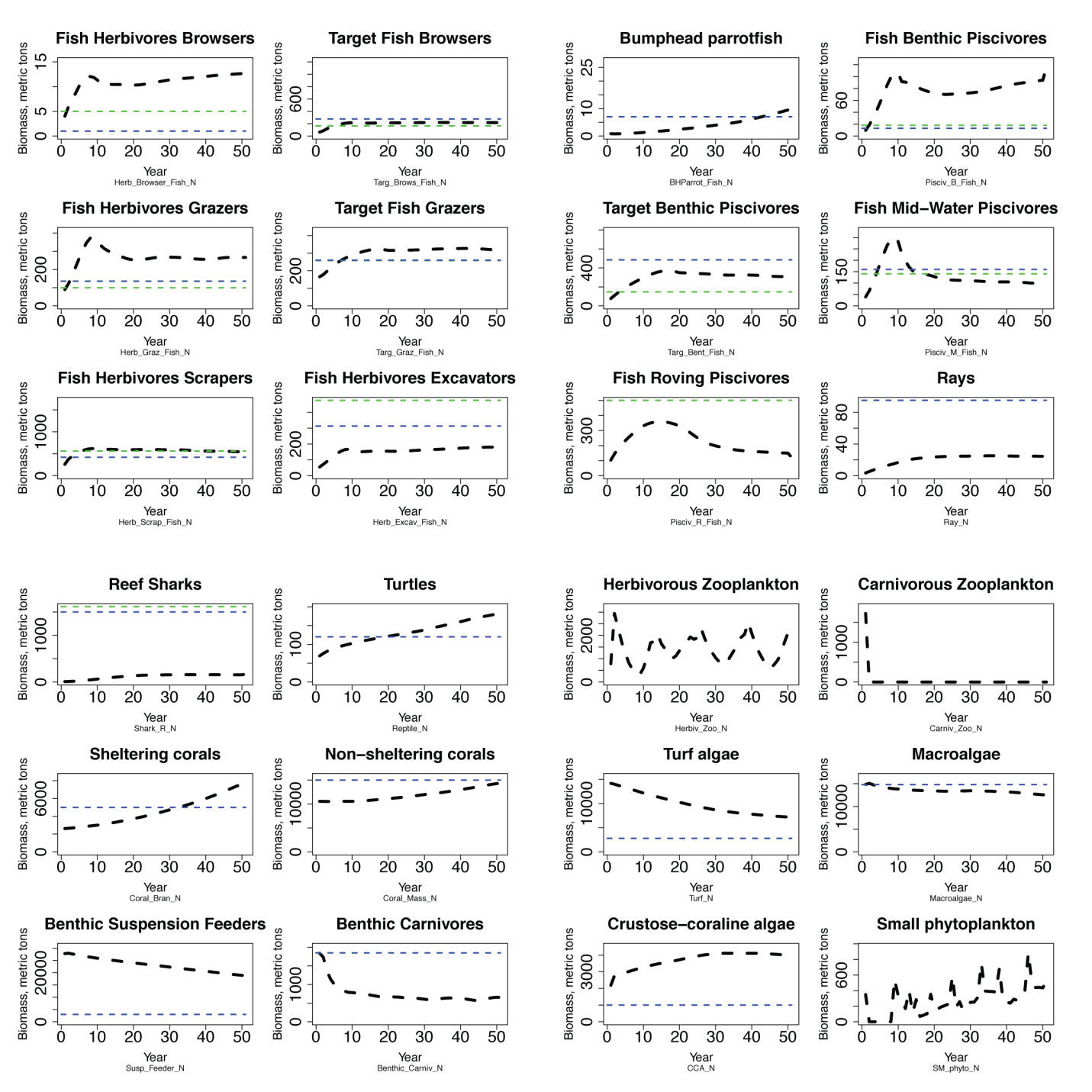


**Fig A2. Biomass trajectories of a simulation with no external disturbances showing some functional groups as examples.**

The green dashed line reflects the biomass in marine preserves in Guam (CRED surveys conducted in 2011). The blue dashed line represents the biomass calculated from surveys around the unpopulated Northern Mariana Islands.

For the vertebrates, the ratio of weight-at-age to initial weight-at-age stayed mostly between the desired values of 0.8 and 1.2, and in all cases between the acceptable levels of 0.5 and 1.5 (Fig A3).


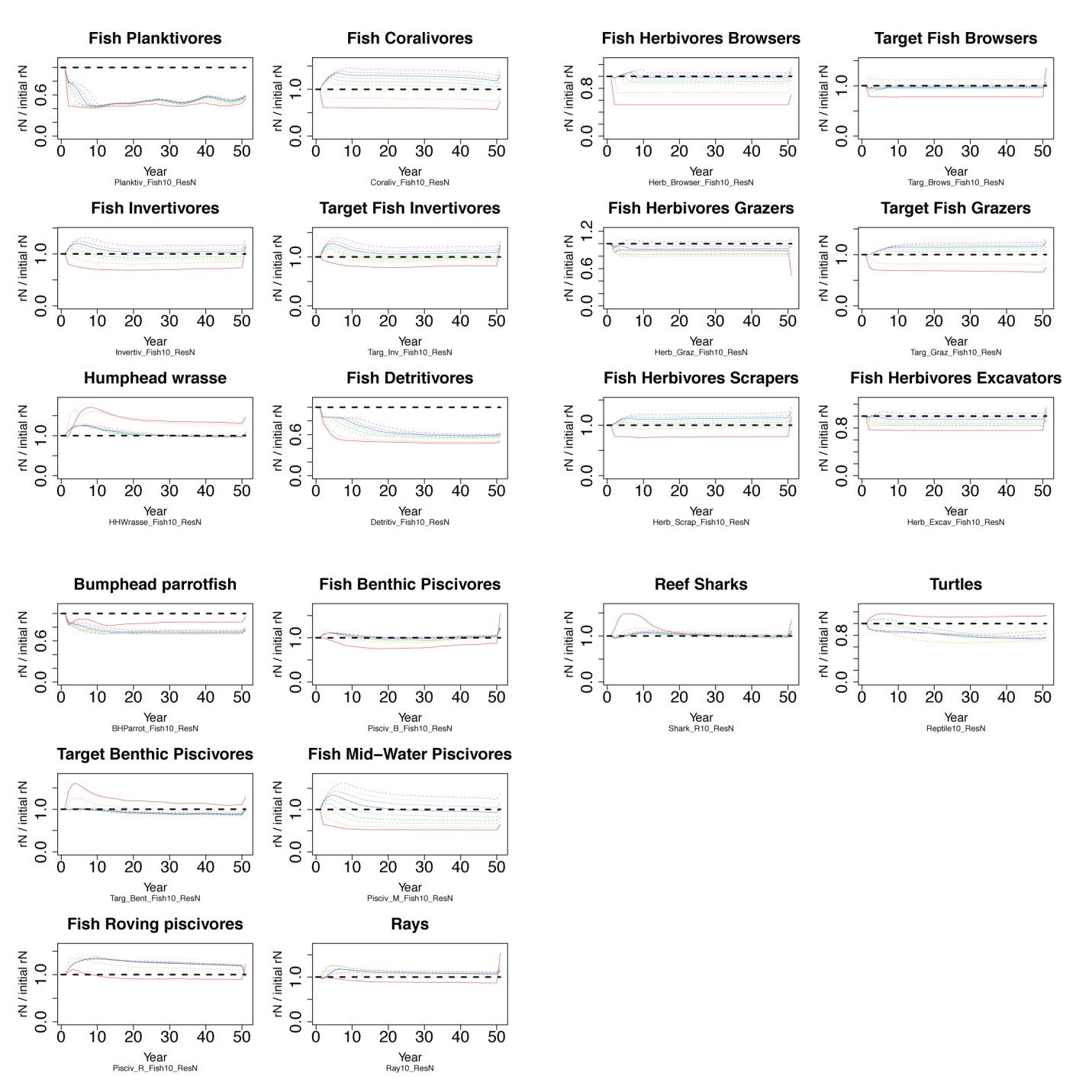


**Fig A3. Trajectory of the ratio of end to initial weight-at-age of vertebrate functional groups of a simulation with no external disturbances.**

The rainbow colors represent the ten age classes with red being the youngest and violet being the oldest age class.

Additionally, the abundance of older age classes was lower than for younger age classes in all fish groups (Fig A4) resulting in an expected size-class distribution based on life-history information. These results satisfied the second criterion for model development.


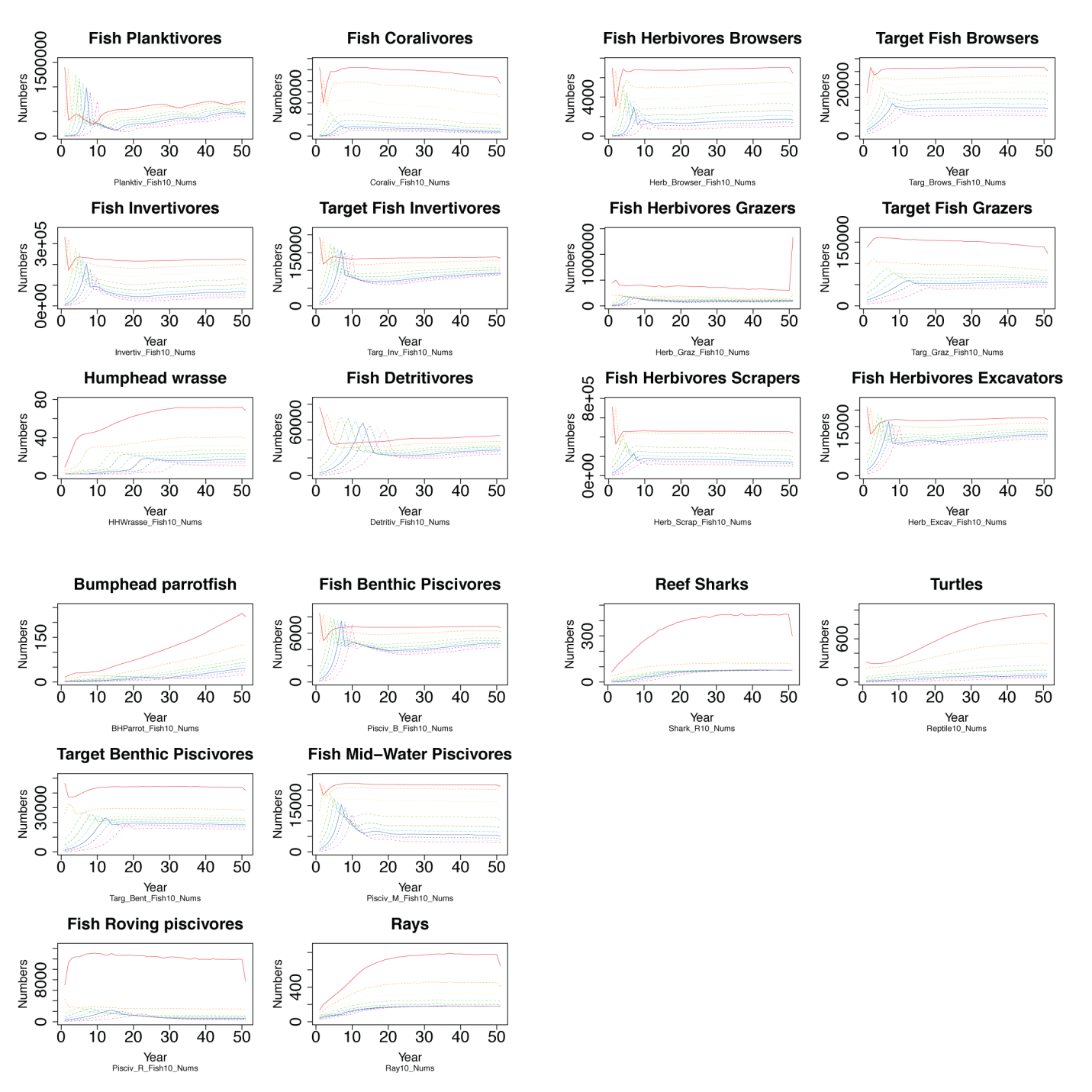


**Fig A4. Trajectory of abundance-at-age of vertebrate functional groups of a simulation with no external disturbances.**

The rainbow colors represent the ten age classes with red being the youngest and violet being the oldest.

Simulating fixed fishing mortality showed that all groups were impacted by fishing pressure. Total fish biomass declined to half initial biomass when fishing mortality was between 0.4 and 1 times natural mortality for each functional group (Fig A5 for targeted functional groups).


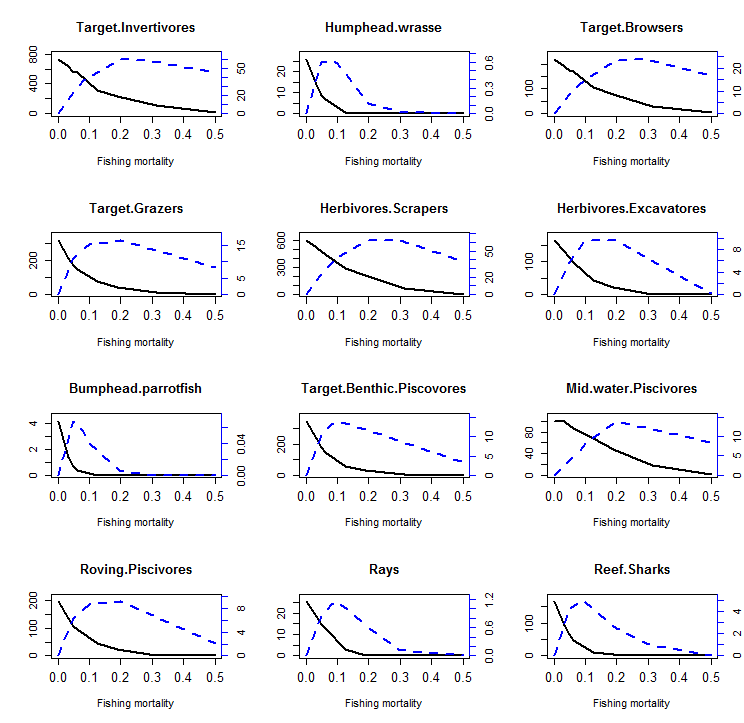


**Fig A5. Catch and biomass equilibrium plots for functional groups.**

Catch (blue dashed line) and biomass (black line) are plotted against fishing mortality rates (F=0, 0.05, 0.08, 0.10, 0.20, 0.30, 0.50, 0.80). X-axis is fishing mortality rate per year, left y-axis is biomass in tons and right y-axis is the catch in tons. Both biomass and catches are shown at the end of a 30-year simulation.

Testing to see if the model could reproduce the historic catches, we calculated the catches from the creel survey data (Guam Division of Aquatic and Wildlife Resources, DAWR) per Atlantis polygon from 1985 to 2013 and forced the model with those catches as time series. Modeled catches were for most of the groups within 20% of the DAWR catch records (Fig A6; shown for some functional groups) but the magnitude of modeled biomass trends for some functional groups did not agree with the estimated relative biomass trends based on the catch-per-unit-effort data in the recreational fishery as measured by the shore-based creel surveys [[17](#_ENREF_17)], i.e., modeled declines were less than estimated biomass trends (Fig A7).


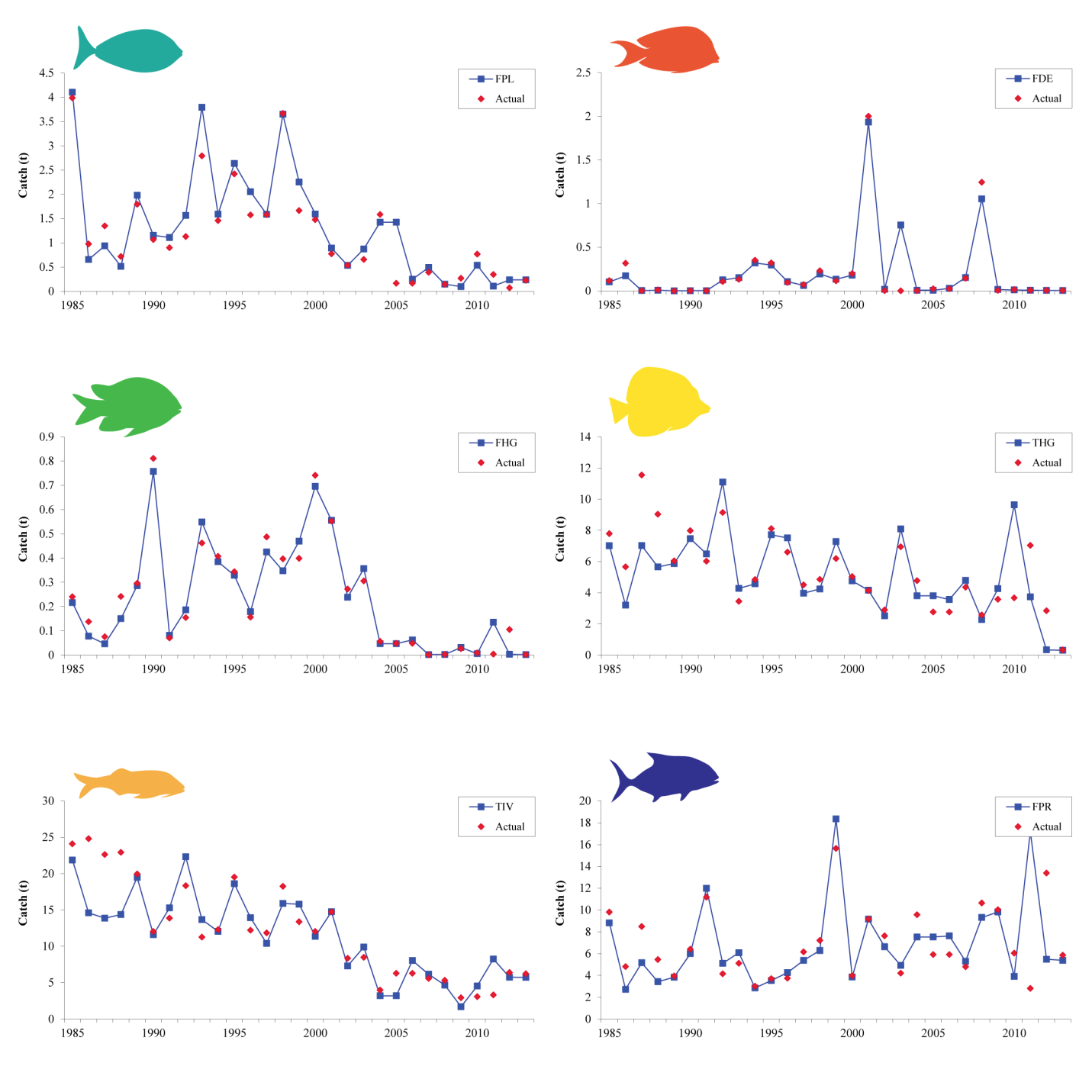


**Fig A6. Annual fish catches from some functional fish groups**.

Blue squares are modeled catches (forced with historical catches as far as possible given modelled ecosystem structure) and red diamonds are reported catches from creel survey data (Guam Division of Aquatic and Wildlife Resources). FPL=planktivores; FDE=detritivores; FHG=grazers; THG=target grazers (e.g., surgeon fishes); TIV=target invertivores (e.g., goat fishes); and FPR=roving piscivores (e.g., jacks).


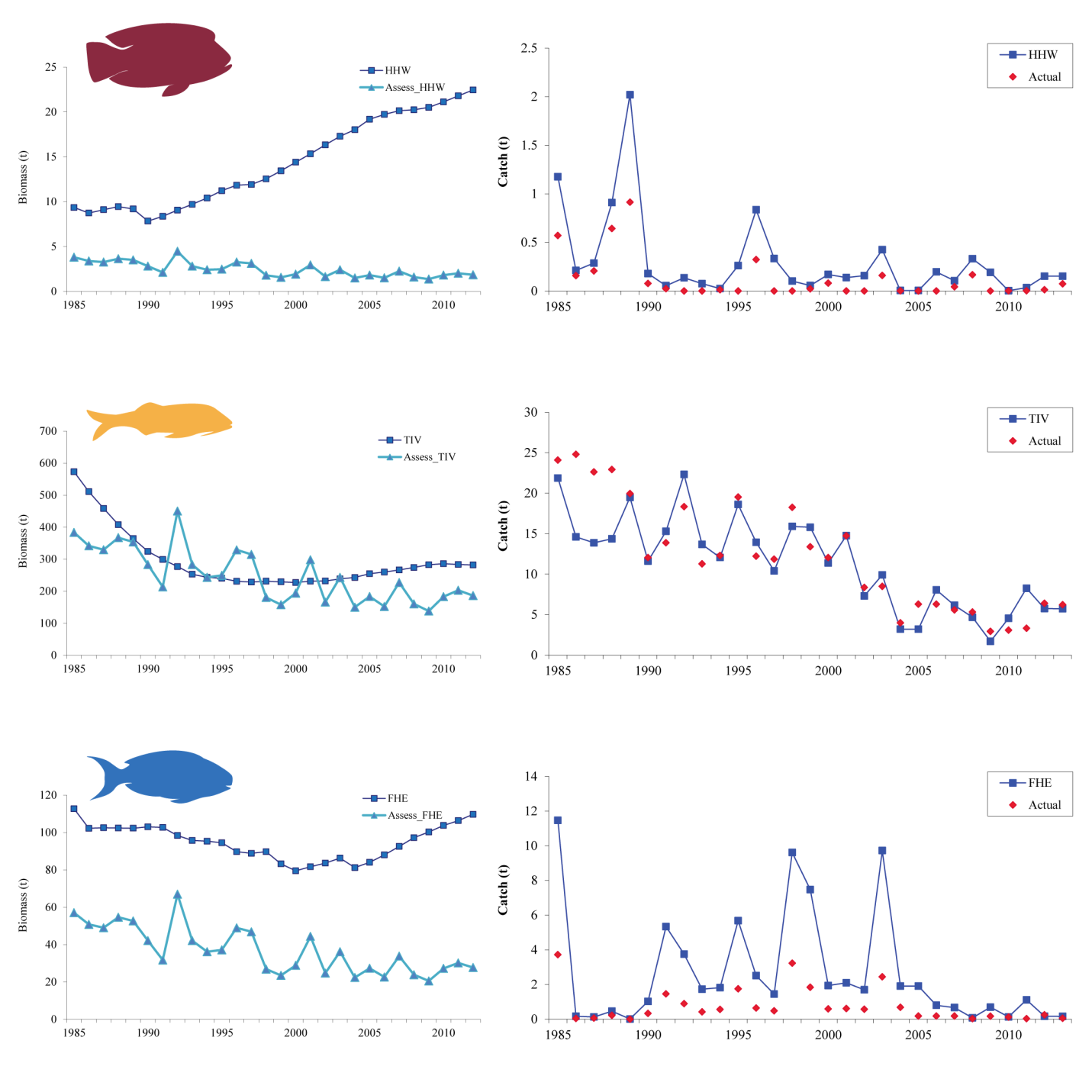


**Fig A7. Standing stock biomass and catch trajectories of a simulation of historical catches**.

*Left panels* show biomass trajectories of modeled (dark blue diamonds) and assumed actual biomass (light blue triangles) and *right panels* show catches (in blue) and estimated actual catches (in red; data from DAWR) of (*top*) humphead wrasse (HHW), (*middle*) targeted invertivores (e.g., snappers, goatfish, emperor; TIV) and (*bottom*) large-bodied parrotfish (FHE)

This discrepancy is similar to a retrospective model of the artisanal reef fisheries of Eritrea in which model trends only matched observed biomass trends when catches were increased to five times reported catches [[18](#_ENREF_18)]. The disparity could be due to underreporting of catches or because we only used the shore-based fishery. It seems justified to increase the fishing effort (and associated fishing mortality) as boat-based reef-fisheries landings show that in total these landings were twice as high as the landings from shore-based fishery (54.0 t vs 27.5 t; average of 2010–2012 DAWR data). However, it is unclear from the boat-based data from which depths or from which spatial zone these fish were taken but as the landings are that much higher it seems safe to assume that at least a part of these landings were taken from the 6–30m depth zone, i.e., the deeper Atlantis polygons in this study. Simulations of increased levels of fixed fishing pressures corresponded well to the expected sensitivity of different groups to fishing pressure, even for the groups that did not show a similar magnitude in biomass after historic catches. This result suggests that the productivity and Beverton and Holt stock assessment parameters are parameterized adequately.

Adjusting the catches mainly from spear fishermen [[19](#_ENREF_19)], resulted in better correspondence with the reconstructed time series of the biomass trajectories (Fig A8) and, therefore, also satisfied criterion 3 of model development. Obtaining improved fishery data for the boat-based fisheries, including allocating these catches spatially, could improve the model. Based on the compliance with all three criteria we concluded that the Atlantis model is stable with plausible biomass trajectories.


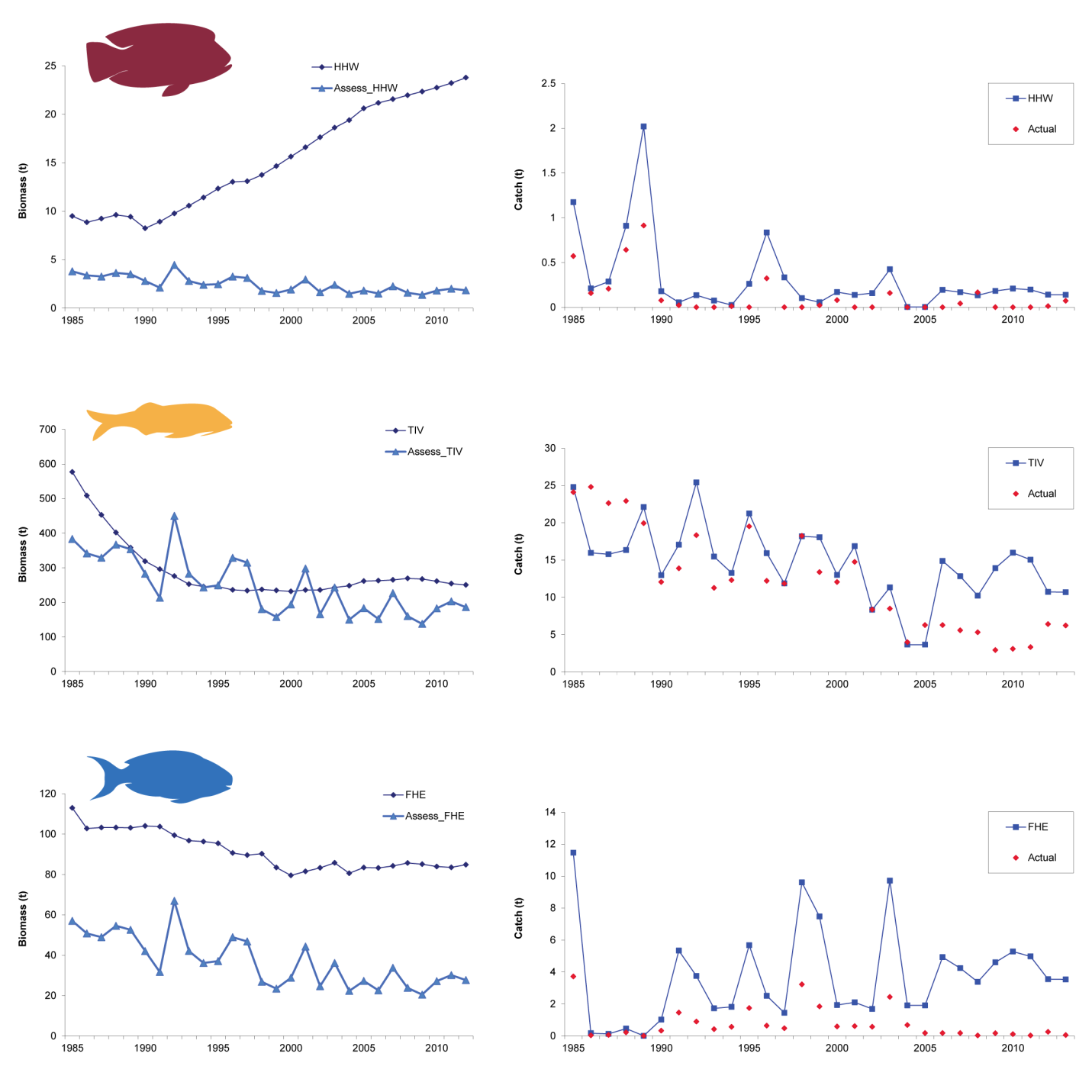


**Fig A8. Results from simulating historical catches with adjusted spearfish catches of the last decade to the same levels as the average of 1999-2004.**

(*Left panels*) Biomass trajectories (in diamonds) compared to reconstructed time series (in triangles) and (*right panels*) the catches (in blue) and estimated actual catches (in red) of (*top*) targeted invertivores (TIV) and (*bottom*) large-bodied parrotfish (FHE).

## Added coral code: Coral related dynamics

After a literature review, we identified key coral reef dynamics and the form of the relationships for those dynamics and added corresponding code (Table A1). These responses came from empirical data or from other modeling studies (Table A1). Coral specific parameters are included in the on-line Supporting Information S2 Table. We detailed the dynamics of coral growth, growth-related complexity, and competition with benthic algae (Figs A9 and A10) that are influenced by three main drivers: (1) climate change (a global stressor indicated in blue in Fig A9); (2) land-based sources of pollution (a local stressor indicated in black in Fig A9); and fishing activities (also a local stressor indicated in black in Fig A9). We acknowledge that we have captured only the main processes and omitted or simplified other processes which influence reefs (e.g., symbionts’ dynamics [[20](#_ENREF_20)], microbe-induced coral mortality [[21](#_ENREF_21)], coral and algal diseases [[22](#_ENREF_22), [23](#_ENREF_23)], linear relationship between herbivore size and bio-erosion [[24](#_ENREF_24)] and others).

**Table A1. Summary of key coral reef ecosystem processes.** These processes are influenced by global and local drivers incorporated in the Guam Atlantis Model. Equations of modeled relationships are in the text.

| **Drivers** | **Rationale** | **Modeled As** | **Source** |
| --- | --- | --- | --- |
| GLOBAL- Ocean warming | Corals are both heterotrophic and, through their symbionts, autotrophic. Depending on the water temperature, duration of exposure to elevated temperatures and coral species sensitivity, symbionts may be expelled (bleaching). Bleached corals have higher mortality rates than healthy corals mostly through increased susceptibility to disease. Mortality and recovery are species specific. | Temperature induced expulsion of symbionts depending on the degree heating weeks, species-specific bleaching-related mortality and recovery depend on duration of elevated temperature. | [[25-33](#_ENREF_25)] |
| GLOBAL - Ocean Acidification | Reduced aragonite saturation results in reduced coral growth and increased bio-erosion. It also has effects on other processes in their life history and on other calcifying organisms or organisms with a calcium skeleton. | Acidification was modeled to affect growth and/or fecundity of corals, plankton groups, crustose-coralline algae, macroalgae, bivalves and urchins. | [[34-47](#_ENREF_34)] |
| LOCAL –  change in framework complexity | Structural complexity is balanced by reef accretion through calcification and erosion through bio-erosion, physical disturbances and predation. For example, coral predation can reduce coral cover by 30% per year.  Coral mortality can lead to a reduction in complexity as the balance between accretion and erosion is tipped towards erosion and hence leads to loss of shelter for fishes, which in turn leads to a loss of fish recruits, abundance and diversity. | Topographical complexity was based on a simplified conical shape of corals increasing in 3 dimensions and hence increasing the complexity. Bio-erosion by cryptic invertebrates, loss of coral growth due to coral predation and destructive fishing practices are modeled as a reduction in complexity. We modelled the relationship between complexity and suitability for refuge for fish, leading to a change in predator availability according to a saturation function with the slope being dependent on a species-specific scalar. Both live and dead corals contribute to complexity. | [[36](#_ENREF_36), [48-58](#_ENREF_48)] |
| LOCAL - Land-based sources of pollution – nutrient increase | Nutrient enrichment favors macroalgal growth over the growth of other benthic groups. Increased macroalgal cover preempts space and increases sediment retention, reducing coral growth, preventing settlement of coral larvae and causes mortality. | Change in coral biomass is related to species-specific coral growth, an inhibition factor from algal groups, an overgrowth factor of macroalgae, mortality of corals and a facilitating factor of coral recruitment onto turf and crustose-coralline algae. | [[15](#_ENREF_15), [21](#_ENREF_21), [59-63](#_ENREF_59)] |
| LOCAL -Land-based sources of pollution – sediment increase | Sediment input hampers corals from growing as they divert energy into mucus production to allow them to slough off sediment particles. Sediment particles in the water column reduce light penetration thus decreasing growth of primary producers (including coral symbionts) | A “smothering effect” that reduces growth according to a linear relationship with the logarithm of sediment level. | [[64](#_ENREF_64)] |
| LOCAL - Fishing - reduced herbivore population | Depletion of herbivores through fishing leads to reduced capacity to maintain turf algae in cropped states and thus leads to elevated standing stocks of macroalgae. | Modeled through trophodynamic relationships (availability matrix) and explicit fishing mortality or catch time series. | [[65-69](#_ENREF_65)] |


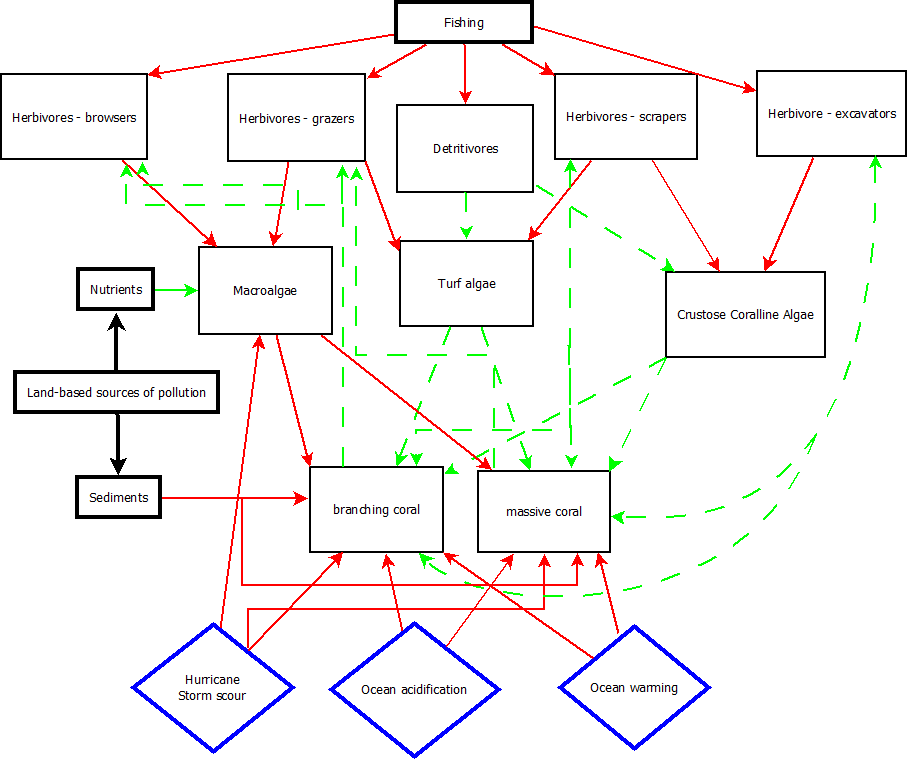


**Fig A9. Conceptual diagram simulating dynamics between benthic groups incorporated in the Guam Atlantis model**.

Depicted are dynamics of macroalgae-coral overgrowth, turf and crustose-coralline algae facilitating coral recruit settlement and the simplified feedback relation between algae and herbivores (herbivores crop algae facilitating coral recruitment and reducing macroalgal overgrowth and corals provide shelter for fish see Fig A10). Red arrows indicate negative effects and green dashed arrows positive effects. Black rectangles are local stressors and blue diamonds are global stressors.


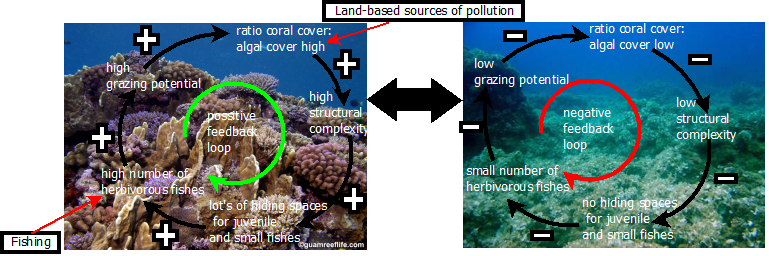


**Fig A10. Conceptual diagram simulating the feedback loop between benthic groups influenced by local stressors (fishing and land-based sources of pollution).**

# Driver 1: Climate change

Climate is predicted to change in many aspects but for this model application, we only look at the effects of ocean temperature and ocean acidification. Naturally, temperature induced stratification (leading to less nutrient in the upper water column) is also of importance but we assume that since we focus on the 0–30 m depth range that these impacts are less important. Hurricanes (typhoons), which are predicted to increase in intensity and frequency, are a natural phenomenon in Guam and can cause extensive damage to the reefs, however, in this version of Guam Atlantis we have not captured those effects.

**Ocean warming**

Corals have physiological processes that are optimized to the local long-term seasonal and inter-annual variations in temperature and an increase of only 1°C or 2°C above the normal local seasonal maximum can induce the expulsion of the symbiotic zooxanthellae [[25](#_ENREF_25)] leaving the coral looking ‘bleached’. Bleached corals are more prone to disease than healthy corals resulting in bleaching-related mortality. Bleaching can be predicted using an index of accumulated thermal stress above a locally established threshold, the Degree Heating Week (DHW) [[31](#_ENREF_31)]. Corals can recover from bleaching episodes [[70](#_ENREF_70)]; however, at some point thermal stress events occur too frequently or for too prolonged a period for corals to be able to recover (we deemed corals to have recovered from a disturbance if their total biomass has reached the same level as before the onset of a disturbance). Projected increases in sea surface temperature for the Central Pacific for the period 2030–2039 vary between 0.6°C and 1.1°C depending on the IPCC scenario [[71](#_ENREF_71)]. Two recent models of the long-term vulnerability of reefs to mortality from bleaching predicted a significant decline in coral reefs [[72](#_ENREF_72), [73](#_ENREF_73)]. However, other studies have shown that corals may have greater ability to adapt to higher temperature than previously believed [[74](#_ENREF_74), [75](#_ENREF_75)] and also that reefs with high structural complexity and at greater depths (> 8 m) are less vulnerable to bleaching impacts [[76](#_ENREF_76)].

In the model, when DHW > threshold, corals will bleach according to Equations 7 and 8:

*Ph_t+1_ = Ph_t_ – Pb* Eq. 7

$Pb = b \bullet(1 + DHW - threshold)$ Eq. 8

with *Ph* = ‘healthy’ or unbleached proportion and *Pb* the bleached proportion; *b* = bleaching rate and *DHW* the degree heating weeks. The threshold is set at 4 for massive corals and 3 for branching corals (Donner et al. 2005).

Of the bleached corals ($Pb\bullet B)$ some will die and some will survive. The overall coral biomass loss due to natural mortality and bleaching induced mortality at time step *i* is calculated as:

$Mi = M\bullet Bcoral +((Bzooxanth \bullet Pb) + (Bpolyp \bullet Pb\bullet Mb))$ Eq. 9

with *M* = mortality rate due natural mortality, *Bcoral* is the sum of the symbiotic zooxanthellae biomass and the polyp biomass, *Bzooxanth* is the symbiotic zooxanthellae biomass and *Bpolyp* the coral polyp biomass, *Pb* the proportion of corals bleaching and *Mb* the bleaching induced coral mortality.

Simulating bleaching events, we assumed that the biomass of symbionts in unbleached corals is 30% of total coral biomass [[7](#_ENREF_7)]; the bleaching threshold temperature is 1°C above the summer maximum (threshold = 30.1°C) [[25](#_ENREF_25)]; the proportion of corals bleaching is 20% for massive corals and 20%–40% for branching corals [[77](#_ENREF_77)]; coral mortality after a bleaching event is 42% for branching and 22.5% for massive corals [[78](#_ENREF_78)]; recovery is believed to occur after 1 year for massive corals and after 4 months for branching corals ([[32](#_ENREF_32), [79](#_ENREF_79), [80](#_ENREF_80)].

Predicted sea surface temperature data came from the Representative Concentration Pathway (RCP) 8.5 projection using the HadGEM-AO model output (data downloaded from the Coupled Model Intercomparison Project Phase5 [CMIP5]: http://apdrc.soest.hawaii.edu/las8/UI.vm), as the 1985–1990 modeled data corresponded well with satellite data from Guam in the same time period. We overlaid this trend on the time series of temperature [[81](#_ENREF_81)] for each Atlantis grid (or polygon) to maintain spatial differences around Guam and thus created a projected temperature time series for each grid cell out to 2050.

**Ocean acidification**

When calcium carbonate saturation state values are < 1, calcium carbonate tends to dissolve and at values > 20 calcium carbonate will spontaneously precipitate [[reviewed in 82](#_ENREF_82)]. However, at values between 1 and 20, calcifying organisms (such as corals) can create calcium carbonate shells or skeletons using a physiological calcifying mechanism. Increased atmospheric CO_2_ leads to a lower pH, which in turn decreases calcification rates of adult and recruits at rates that vary among coral species (reviewed in Brainard et al. 2011). Evidence from numerous studies of calcifying organisms has suggested that corals affected by reduced saturation state may primarily experience reduced growth [[37](#_ENREF_37), [39](#_ENREF_39), [44](#_ENREF_44), [83](#_ENREF_83)], although many uncertainties remain especially since synergistic effects should be taken into account [[46](#_ENREF_46), [84](#_ENREF_84)], effects differ geographically [[85](#_ENREF_85)]and some corals still calcify when provided with sufficient food supplies [[86](#_ENREF_86)].

Apart from the added code for the relationship between coral growth and pCO_2_ (see below) we also parameterized the pCO_2_ relationships (based on changes in pH) with various functional groups (S1 Table). Marine species most affected by ocean acidification are calcifying organisms (corals, echinoderms, mollusks), which exhibit negative relationships between ocean acidification and growth and calcification rates [[43](#_ENREF_43), [44](#_ENREF_44), [47](#_ENREF_47)]. Declines in growth rates of crustose coralline algae by 86% and rhodoliths by 100% have been reported [[37](#_ENREF_37), [44](#_ENREF_44)]). In contrast, phytoplankton and fleshy macroalgae are likely to grow more rapidly in more acidic water [[43](#_ENREF_43), [46](#_ENREF_46)]. Rates of herbivory necessary to maintain successful coral recruit settlement would have to increase as atmospheric CO_2_ increases. Crustaceans did not show a significant negative effect of ocean acidification on growth or calcification [[44](#_ENREF_44), [46](#_ENREF_46)] or were reported to have a reduced sensitivity compared to corals, mollusks and echinoderms [[47](#_ENREF_47)]. Reproduction rates declined for urchins and copepods with increasing pCO_2_ [[43](#_ENREF_43), [87](#_ENREF_87)]. We included nonlinear response relationships between growth and increased pCO_2_ for phytoplankton and macroalgae, a linear decline of growth for benthic filter feeders (including bivalves) and crustose coralline algae, a linear decline in growth and fecundity for benthic grazers (urchins), and a Monod response effect on growth and fecundity for herbivorous zooplankton groups (copepods) [[47](#_ENREF_47)] (Fig A11).


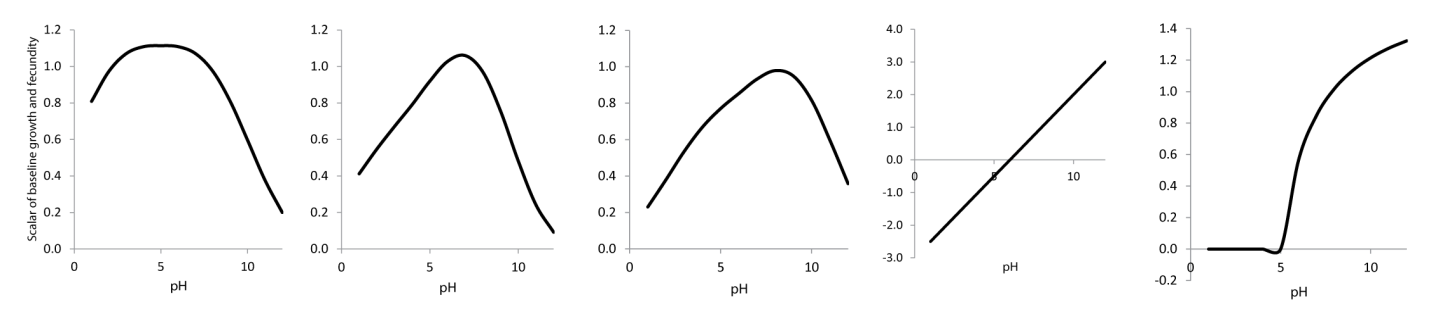


**Fig A11. Relative shape of the effect curve of pH on various groups**.

Groups are from left to right (*A*) small phytoplankton, (*B*) large phytoplankton, (*C*) macroalgae, (*D*) benthic filter feeders, crustose-coralline algae and benthic grazers, and (*E*) herbivorous zooplankton. The growth rate and/or fecundity is multiplied with the scalar corresponding to the current pH at each time step in the model to account for the pH effects. If this scalar is negative or equal to zero, the growth rate is multiplied with a very small number to approximate zero in effect but to avoid the numerical problems created by division by zero elsewhere in the code.

To approximate a range of qualitatively different functional responses of corals to the aragonite saturation state (*Ω*) and temperature (*T*) [[88](#_ENREF_88)] we modeled the net calcification rate (*Gnet*) with equations 10–13:

$Gnet =K_{T}\bullet(\Omega- 1) ƛt * \frac{T}{Topt}$ Eq. 10

where *T* is the current temperature and *T_opt_* the optimum temperature and *K_T_* and *ƛ* are temperature-dependent functions accounting for the strength and shape of the calcification response to variation in *Ω* and *T*. We assumed that *T_opt_* is near the summer maximum non-bleaching temperature of 29.1°C (<http://coralreefwatch.noaa.gov/satellite/vs/guamandcnmi.php#GuamEast>). The temperature response of *K_T_* was assumed to be symmetrical around the optimal temperature for calcification and is given by:

$K_{T} = \alpha- \beta\bullet\frac{{(T - Topt)}^{2}}{Topt}$ Eq. 11

where *α* and *β* are regression parameters.

The aragonite saturation state is calculated as the product of the calcium and carbonate ions divided by the solubility constant (*K_sp_*):

$\Omega=\frac{\left[ {Ca}^{2+} \right][{CO}_{3}^{2-}}{K_{sp}}$ Eq. 12

where the CO_3_^2-^ concentration and pH change according to the Bjerrum plot [[42](#_ENREF_42)] which we reproduced using 250 µmol/kg reported for modern day CO_2_ concentration at 23°C and a pH of 8.068 for the midpoint of the curve [[40](#_ENREF_40)].

The pCO_2_ concentration we used comes from the IPCC AR5 report using the highest emission scenario (RCP8.5) projection.

Finally, relative coral growth is calculated as:

$\mu CT = \mu C \bullet\frac{G_{net}}{G_{base}}$ Eq. 13

with *G_net_* calculated by Equation 10 and an assumed base calcification rate (*G_base_*). Values used for the parameters are given in S2 Table.

## Climate change effects on coral reef ecosystems

Both ocean acidification and elevated temperatures had a negative effect on coral biomass with coral bleaching leading to abrupt mortalities and slow recovery until bleaching events were too frequent for corals to recover (after 48 years, i.e., from 2023 onwards, Fig A12). Acidification led to a more continuous gradual decline in coral biomass caused by reduced calcification (Fig A12). The declines caused by acidification were weaker than those due to bleaching, though as discussed below we may be underestimating acidification (modeled reduction of pH to 7.95 in 2050). Macroalgal biomass increased when space was opened due to bleaching induced coral mortality (solid lines Fig A12) and also increased when coral biomass was reduced due to ocean acidification. Crustose-coralline algae, which are negatively affected by ocean acidification, did not show a decline in biomass but instead increased in biomass when space became available after corals bleached and died.

Modeled recovery after a bleaching event took 2–9 years, which falls within recovery times seen in Palau [[89](#_ENREF_89)] and what has been reported from Guam [[90](#_ENREF_90)]. Our results fall within the spread found for model simulations of IPCC scenario AR8.5 for various Pacific reefs with coral decline predicted to occur between 2030 and 2050 based on bleaching related mortality alone [[91](#_ENREF_91)]. The first reported bleaching events in Guam with no coral mortality were in 1994 and 1996 [[90](#_ENREF_90)] and more frequent bleaching events were observed in the shallow bays in September 2006, August and September 2007 and wide-spread bleaching occurred in the summer of 2013 and 2014 (V. Brown, NOAA Pacific Islands Regional Office pers. comm. Nov. 2014). Elevated temperatures which led to those bleaching events are shown in the satellite time series data as peaks above the red dashed threshold line in Fig A12’s top panel. The response difference between the branching and massive corals is due to the difference in susceptibility to elevated temperatures and mortality rate after bleaching with (branching corals being more susceptible to bleaching and have a higher mortality rate and hence, the drop in coral biomass in larger for the branching corals [Fig A12]).

Guam Atlantis projected a reduction in coral biomass of 2%–4% as a result of ocean acidification. This appears less than predicted in other published studies that show an expected reduction of 22%–39% in calcification and a 47% reduction in abundance of coral recruits [[84](#_ENREF_84)]. Since we report on total coral biomass in the model domain, direct comparison is difficult—a 25% reduction in calcification does not automatically translate to a decrease in coral biomass, the coral skeleton is likely less robust due to decreased calcification but the actual live coral tissue could be similar. However, the IPCC scenario and algorithms that we used, led to a decrease in aragonite saturation of only 0.3 units corresponding to a pH decrease from 8.19 in 1975 to 7.95 in 2050, whereas most laboratory studies assume a pH decrease of at least 0.5 units. Aragonite saturation states are predicted to decrease by approximately 1.0 unit by the end of the century [[100 years; 92](#_ENREF_92)], so we likely underestimated the reduction in aragonite saturation.

The combined effect of temperature and ocean acidification did not differ markedly from the (only) elevated temperature scenario (Fig A12, bottom panels). Laboratory studies have shown that elevated temperature increases calcium carbonate precipitation and could therefore offset the effects of reduced calcification [[84](#_ENREF_84)]. Certainly, temperature differences can confound the relationship between pCO_2_ and calcification [[93](#_ENREF_93)]. In our model the average summer temperature of 29.4°C in Guam was very close to the modeled optimum calcification rate at 29°C [[41](#_ENREF_41), [94](#_ENREF_94)] (S2 Table) which could be a reason why the coral biomass trajectory of only elevated temperature is similar to the trajectory of the combined effects of ocean acidification and elevated temperature. Also nutrients influence the calcification rate [[39](#_ENREF_39), [86](#_ENREF_86)] and Guam was modeled to have quite high concentrations of nutrients in the shallow polygons so their growth due to filter feeding could also have offset the negative impact of ocean acidification. In future versions of the Guam Atlantis model, alternative time series of predicted pH and/or aragonite saturation states could be used as input values to get a better understanding of the variance in the effects size of calcification and coral growth and the synergistic effects of ocean acidification and ocean warming. For the application of the current model, we are confident that the predicted effects of ocean warming are well captured and, since current knowledge suggests that they are acting as a primary course of coral decline in the near future [[95](#_ENREF_95)], we think that future trajectories are reasonably projected.


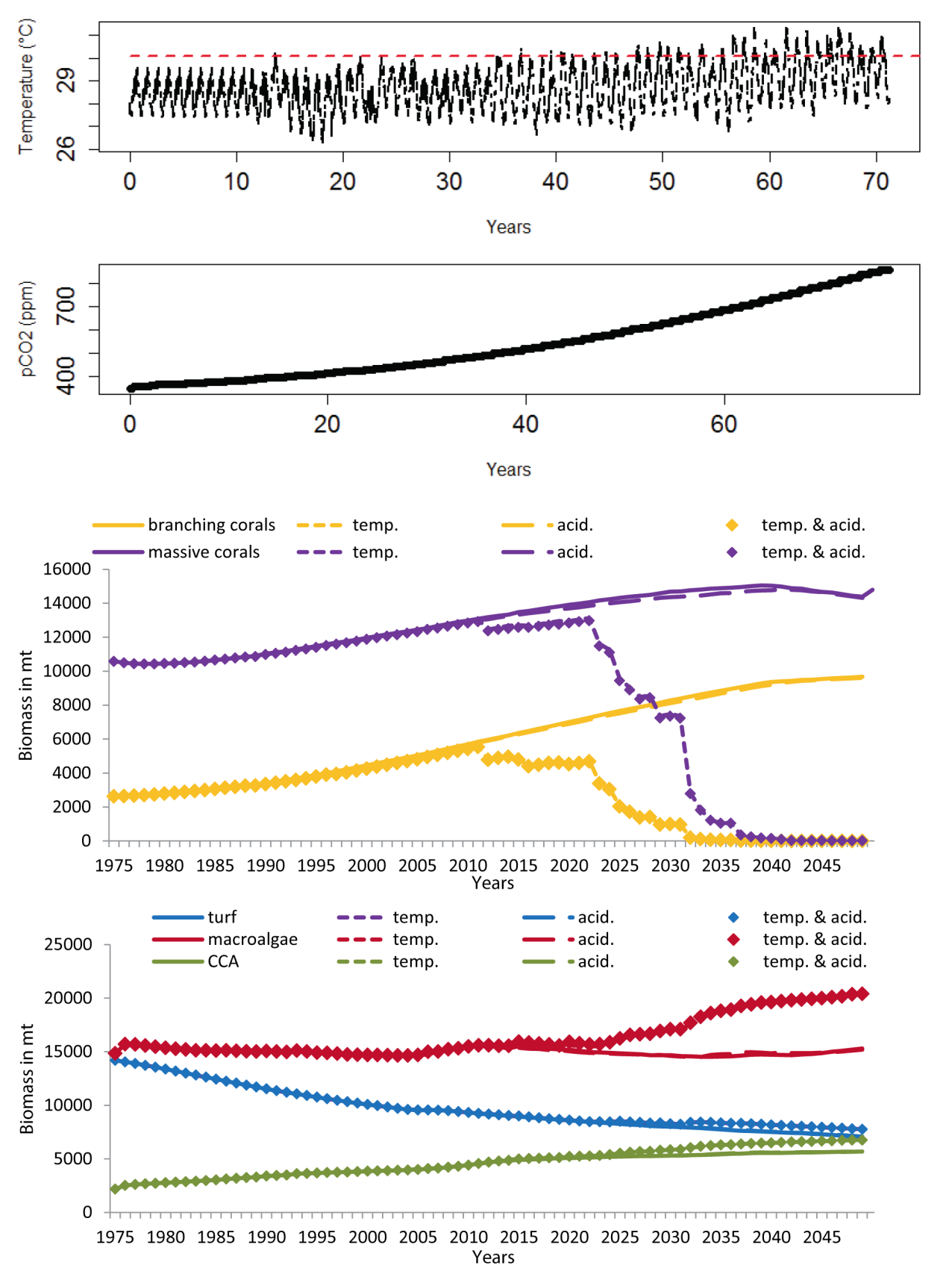


**Fig A12. Predicted climate change and modeled response of algae and corals.**

(*Top panel*) Time series of predicted sea-surface temperature (HadGEM-AO) with bleaching threshold at 30.1°C (red dashed line) and (*second panel*) pCO_2_ trajectories (IPCC AR8.5) were used to force the model with. (*Bottom two panels*) Effects of elevated temperature (dashed lines), ocean acidification (long dashed lines) and the combined effects of ocean warming and acidification (diamonds) on the benthic communities compared to control run (solid lines). Year 0 = 1975.

# Driver 2: Land-based sources of pollution

Changes in land use can alter runoff of nutrients and sediments into coastal waters. The combined effects of elevated nutrients (bottom-up processes) and grazing (top-down processes) influence the physical and ecological controls of macroalgal dynamics [[96](#_ENREF_96), [97](#_ENREF_97)]. Higher concentrations of sediments and nutrients favor macroalgal growth over coral growth [[68](#_ENREF_68)] and enhance coral disease [[22](#_ENREF_22)] resulting in decreased coral cover (Table A1). Sediments are also detrimental for the settlement of calcifiers (corals and CCA) [[98](#_ENREF_98)]. When substrate sediment load is low, coral recruits have a higher survival rate [[68](#_ENREF_68), [98](#_ENREF_98)]. Time series of sediment and nutrient input to the marine coastal areas around Guam were based on river flow rates and sewage outfall pipes [[99](#_ENREF_99)]. Based on an absence of clear trends in the time series of river outflow rates (Fig A13) we repeated the last year for future simulations.

**Fig A13. Simulated river outflow of the (*top panel*) Pago river watershed on the east coast and the (*bottom panel*) Anahatan river watershed on the west coast of Guam.**

For the benthic groups included in this model—corals (C), macroalgae (MA), crustose coralline algae (CCA) and turf algae (T)—biomass is associated with the physical space they occupy in the reef. The biomass dynamics of these groups are determined by trophic interactions and metabolic processes and are density dependent. We defined turf cover as all hard substrate that is not any other benthic category (Eq. 14):

T=1–MA–CCA–C Eq. 14

Coral-algal dynamics were simulated by changes in biomass with macroalgae overgrowing corals with a rate of α and hampering growth of corals with a rate of *β* and corals recruiting to turf and CCA with rate γ [[15](#_ENREF_15)] and no recruitment in macroalgal habitats [[100](#_ENREF_100)]:

$\frac{dC}{dt}=\mu\bullet C-Mlin-C-Mquar-C-Predation-\alpha\bullet MA-\beta\bullet MA+\gamma\bullet(CCA+T)$ Eq. 15

$\frac{MA}{dt}=\mu\bullet MA-Mlin-MA-Mquar-MA-Predation+\alpha\bullet MA+\beta\bullet MA$ Eq. 16

where *µ* is the growth rate, C is the biomass of corals, *MA* is the biomass of macroalgae, CCA the biomass of crustose coralline algae, T the biomass of turf algae and *M_lin_* and *M_quar_* are the linear and quadratic species-specific mortality rates. Furthermore, growth of primary producers is dependent on light, space and nutrient limitation (full description of these dynamics is detailed in [[101](#_ENREF_101)]). We allowed for vertical growth, for example, on the base of branching corals, under or at the sides of overhangs and boulders, by setting the total benthic space available to 135% of planar area.

Coral growth can be further hampered by sediment. High sediment concentration in the water column not only limits the light available for photosynthesis (captured in Atlantis under light limitation), when sediment settles on the reef, corals have to divert some energy to producing mucus to slough off those sediments. This energy is then not available for growth. Using the empirical relationship derived from a study of sediment concentration and change in coral abundance conducted in Guam (derived from Equation 1 in [[64](#_ENREF_64)]) we included a function to capture this ‘smothering effect’:

$S = Sa \bullet ln(sed\_level) + Sb$ Eq. 17

where *S* is the smothering effect, *Sa* the smothering coefficient and *Sb* the smothering constant. The *sed_level* is the concentration of sediments in the water column and changes over time mostly depending on riverine runoff.

We simulated two scenarios: (1) with estimated time series of river and sewage outflow that contained sediments and nutrients [[99](#_ENREF_99)], and (2) no inputs. Parameter estimates are given in S2 Table.

## Land-based sources of pollution: Sedimentation and nutrient elevation effects on benthic composition and target fish biomass

Point source and non-point source pollution and accompanying elevated nutrient and sediment input into nearshore waters led to a rapid increase in phytoplankton and suspended solids followed by an increase in macroalgae and CCA and a decrease massive corals (Fig A14).


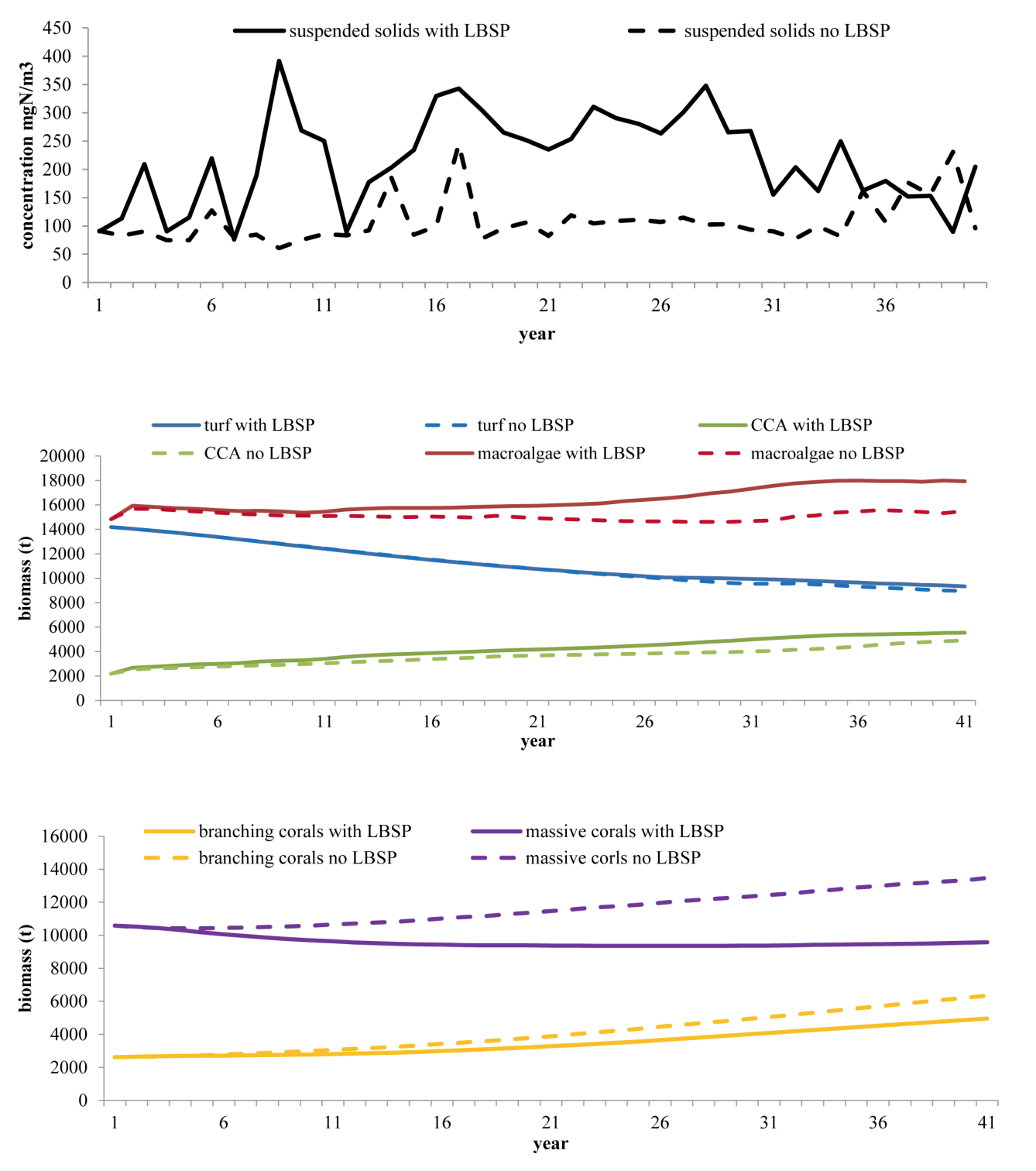


**Fig A14. Simulation of point source pollution into the shallow nearshore waters of Guam**.

Results contrast the concentration of (*top*) suspended solids, (*middle*) and biomass of algae and (*bottom*) corals between two simulation runs of 40 years: (dashed lines) no land-based sources of pollution (LBSP) and (solid lines) with LBSP. Year 0 = 1975.

Increased sediment led to reductions in coral growth rates compared to the control scenario (Fig A14). Consistent with nutrient enrichment experiments [[102](#_ENREF_102), [103](#_ENREF_103)] we had expected algal biomass to positively respond to nutrient input and that to lead to reductions in coral cover, the effects of which were shown by our model (Fig A14). The effect of sediments on the growth rate of corals and the competition between corals and algae are modeled using the same parameters, since massive corals have a lower growth rate, the effect is larger on massive corals.

Results from surveys of reef flats in Guam along a gradient away from sewage outfall pipes showed reduced coral cover close to the outfall pipes (0.13 km) between 2009 and 2010 and no significant difference in percent coral cover away from the outfall pipes (1.13–11.3 km)[[104](#_ENREF_104)]. In correspondence with those local results, the model appears to capture the effects of LBSP well with low coral cover in the southern polygons relative to the north and east of Guam.

# Driver 3: Fishing

Parrotfish, grouped in large excavators and scrapers, fulfil an important ecological role by opening up substrate for coral recruitment (Table A1). The feedback loop between corals, algae and grazers is possibly the most examined reef dynamic with minimal models, exploring the existence and thresholds of stable states [[24](#_ENREF_24), [96](#_ENREF_96)]. Cover of macroalgae on reefs is linearly negatively related to the biomass of herbivorous fishes [[24](#_ENREF_24), [65](#_ENREF_65)]. However, studies in the Caribbean have shown that herbivorous fishes can only maintain a maximum of 30%–40% of a reef structure in a cropped state [[105](#_ENREF_105)]. Large parrotfishes are more effective grazers than small ones [[52](#_ENREF_52)], for example, 75 small (< 15 cm) parrotfishes maintain the same area of substrate in grazed states as one single individual of 35 cm [[106](#_ENREF_106)]. In locations with high fishing pressure, large parrotfish are removed and, although small parrotfishes will tend to increase in abundance due to predator release [[65](#_ENREF_65)], the net effect of fishing pressure leads to reductions in grazing pressure potentially leading to phase shifts in algal-coral cover.

Corals have a positive effect on fish abundance by providing shelter [[57](#_ENREF_57), [107-110](#_ENREF_107)]. Several studies have also demonstrated a positive relationship between coral complexity and fish diversity [[50](#_ENREF_50), [111](#_ENREF_111), [112](#_ENREF_112)], especially for corallivores and planktivores [[113](#_ENREF_113)]. This positive feedback appears to be driven by the increased survival of fish recruits and other small-bodied fishes [[50](#_ENREF_50), [54](#_ENREF_54), [113-115](#_ENREF_113)]. Coral reef complexity is partly dependent on the topography of the reef substrate itself (e.g., boulders provide complexity) and partly by coral growth (e.g., large branching corals provide more shelter than small coral colonies). Benthic complexity values ranging between 1 (flat) and 5 (high structural complexity) were taken from CRED survey data and serve as a baseline. We then assume that changes in complexity reflect net coral growth (i.e., the balance between accretion and erosion). We used the topographical complexity model developed by Bozec et al. [[56](#_ENREF_56)] to calculate rugosity.

$R = rug\_constant \bullet SI$ Eq. 18

where *R* = rugosity, *rug_constant* is the ratio of the vertical contour of a colony to the surface-to-area ratio of a colony, *SI* is the deformation of the reef surface and is calculated as the surface area of the reef (depending on height and diameter of coral colonies) divided by the planimetric area of the reef which depends on the maximum diameter and coral cover [[56](#_ENREF_56)].

Rugosity in turn affects the availability of prey to predators, according to the inferred relation of the habitat scalar at time *i* based on [[54](#_ENREF_54), [113](#_ENREF_113)]:

$hab\_scalari = hab\_scalar \bullet(Rcoefft \bullet ln(R) + Rconst)$ Eq. 19

In other words, rugosity affects the availability of prey fish to predators according to the inverse of the habitat scalar relationship with a maximum capacity set at 4 and a scalar coefficient depending on the species (varying between 0.6 for small species to 8.0 for unaffected species). For parameter estimates, see S2 Table.

To determine the adapted model’s capability to simulate the effects of the coral-algae-grazer relationships with the added code, we simulated two scenarios: (1) intensive fishing on herbivores (fishing mortality (F) of 0.8 per year) and (2) no fishing of herbivores. In both scenarios we kept fishing on other groups low (F=0.05 per year). Similarly for the relationship between complexity and fish biomass, we ran two 30-year simulations, one with this relationship included and one without, assuming a low fixed fishing mortality of 0.05 for all groups.

## Effects of fishing of herbivores on corals-algae-grazers dynamics

The strong link between herbivorous fishes and the benthic community composition (high herbivore biomass resulted in low macroalgal cover and high coral and CCA cover) is clearly demonstrated in the scenarios with high (F=0.8) and no (F=0) fishing of herbivorous fishes while keeping fishing on all other targeted groups to F = 0.05. Compared to no fishing of herbivores (F=0), when herbivore fishing mortality was set to 0.8, biomass of turf and macroalgae is 6% higher and biomass of calcifiers is 7% lower at the end of a 30-year simulation.

Scenarios with and without refuge effects from structural complexity showed the importance of incorporating the effects of complexity into coral reef ecosystem models. Fish biomass of most prey groups was enhanced by the three-dimensional complexity (Fig A15). Planktivores in particular were dependent on refuge associated with structural complexity, as their biomass was 6.6 times more in models with the complexity-refuge relationship enabled. In contrast, predatory fish groups had a 0.1–0.5 times lower biomass when habitat refugia relationships were included in models (Fig A15). Modeled increase in biomass of prey fish with increased structural complexity was in correspondence with empirical relationships from Guam (Fig A16).


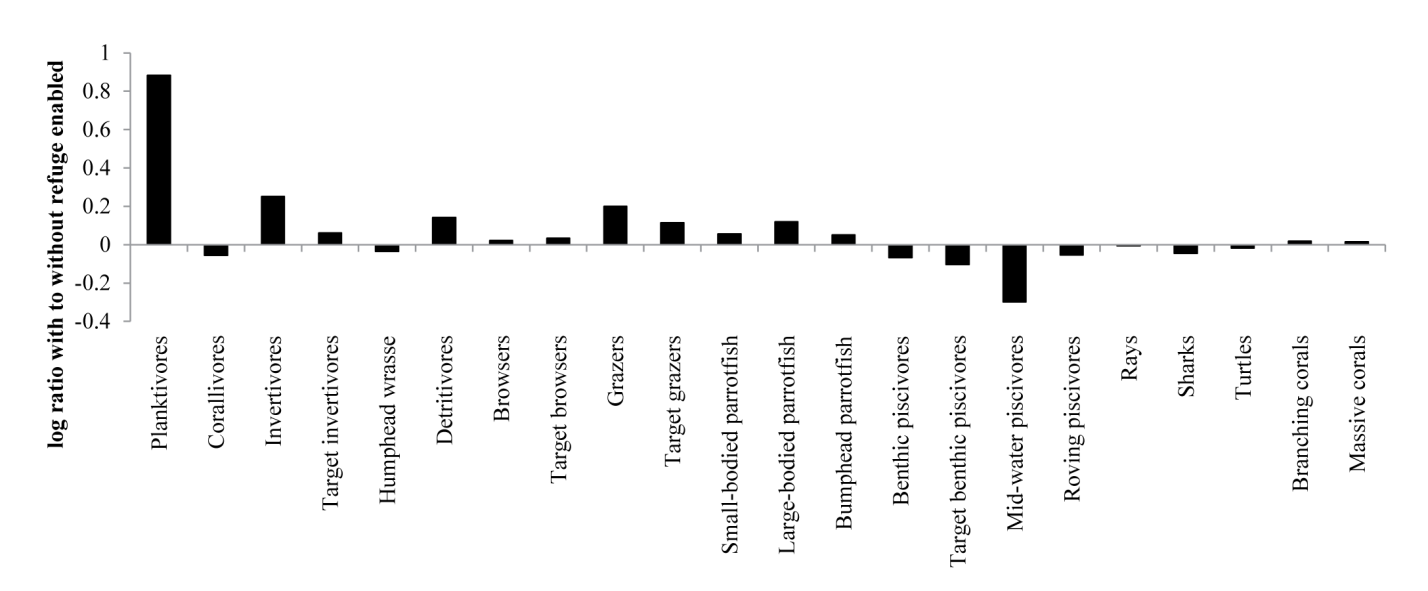


**Fig A15. Influence of refuge on fish biomass.**

The y-axis is the ratio of biomass with refuge enabled to no refuge enabled for each functional vertebrate group and corals (y-axis) at the end of 40 year simulation run. Ratios are represented as logarithmic values of the actual ratio.


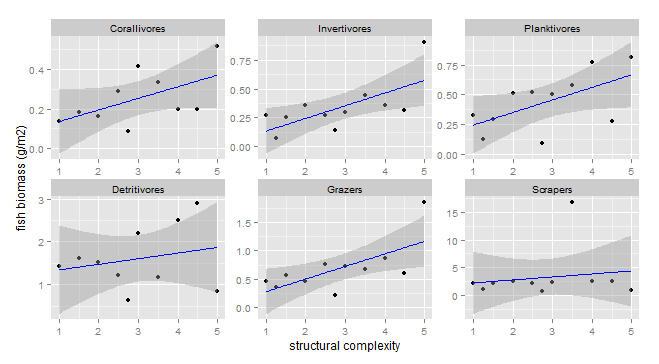


**Fig A16. Relationships between the structural complexity and the reef-fish biomass of six functional groups around Guam.**

Blue line is linear regression line with shaded area representing 95% confidence region. Data from visual surveys conducted by NOAA-PIFSC-CRED in 2011.

Simulations demonstrated strong links between herbivorous fish biomass and the benthic community, with herbivore biomass associated with low macroalgal cover and high cover of coral and CCA, as has been shown on reef in American Samoa [[69](#_ENREF_69)], the Caribbean [[68](#_ENREF_68)], the Great Barrier Reef [[116](#_ENREF_116)], Fiji [[117](#_ENREF_117)], but also in spatial surveys conducted around Guam [[118](#_ENREF_118)].

Additionally, model outcomes show the importance of reef structure as refuge for fish species, by making them less vulnerable to predators. We parameterized this part of the model based on the study of Bozec et al. [[56](#_ENREF_56)], but those relationships could potentially be improved with site-specific data. Also the parameters used for erosion caused by fish, urchins and boring sponges that affect the reef’s rugosity should be updated with data from Guam when such data becomes available to improve the model.

# Sensitivity and skill assessments

We performed sensitivity assessments on primary productivity (growth rate of primary producers), the structural complexity input parameters (diameter of corals) and the parameter for direct coral-algal competition (the *β* in equation 15). From the literature we derived plausible values for these parameters (S2 Table) and ran the model with the minimum and maximum estimates to get boundaries for plausible trajectories. We then visually inspected the derived cone of uncertainty (i.e., range of trajectories) to see which of the groups are sensitive to the parameters and if the trajectory of the control run fitted into this cone.

To assess the model skill, we compared (1) the projected biomass of each fish group against the observed biomass from CRED fish surveys conducted in 2011 and (2) projected massive coral biomass standardized to the mean against observed biomass also standardized to the mean from CRED benthic surveys using line-point-intercept method conducted in 2005, 2007, 2009, and 2011. For comparisons we used the root mean squared error (RMSE) according to:

$RMSE= \sqrt{\frac{\sum_{i=1}^{n} \left( P_{i}-O_{i} \right)^{2}}{n}}$ Eq. 19

where *n* = the number of observations (fish groups), *O_i_* = the *i*^th^ of *n* observations, *P_i_* = the *i*^th^ of *n* projections and *O* and *P* are the observation and projected annual averages, respectively [[119](#_ENREF_119)].

Out of the three sets of parameters analyzed with a sensitivity assessment, only the growth rates of plankton showed substantial variation in outcomes of selected fish groups and coral biomass (Fig A17). For the rugosity sensitivity (the parameter inputs being the mean diameter for branching and massive coral colonies) the outcomes varied a little (mostly for the rugosity; Fig A18) and target fish groups and corals were not sensitive to the parameter for coral-algal competition (Fig A19). When varying the growth rates of phytoplankton, most vertebrate groups showed a narrow cone of uncertainty, i.e., they are not sensitive to changes in these growth rates (Fig A17). Our control run estimates were in the middle of the cone for 10 out of the 13 target fish species, giving more confidence in the model results with regard to fish groups. However, coral cover and, hence, rugosity (or structural complexity) are much more sensitive to these changes making it important to get good estimates of the growth rates of these groups. Since we did not run the control scenario with the mean of the primary producers’ growth rates, but rather the maximum of large phytoplankton and lower than the average for small phytoplankton, the predicted biomass (red line in Fig A17) does not necessarily need to be in the middle of the cone. Future versions of Guam Atlantis should look more into the effect of the predicted variation in coral biomass as a response to the changes in growth rates of primary producers to help explain the high biomass trajectory in corals (top or above the cone) and the odd shape of the relationship between planktivores and plankton (where one could expect a linear relation).


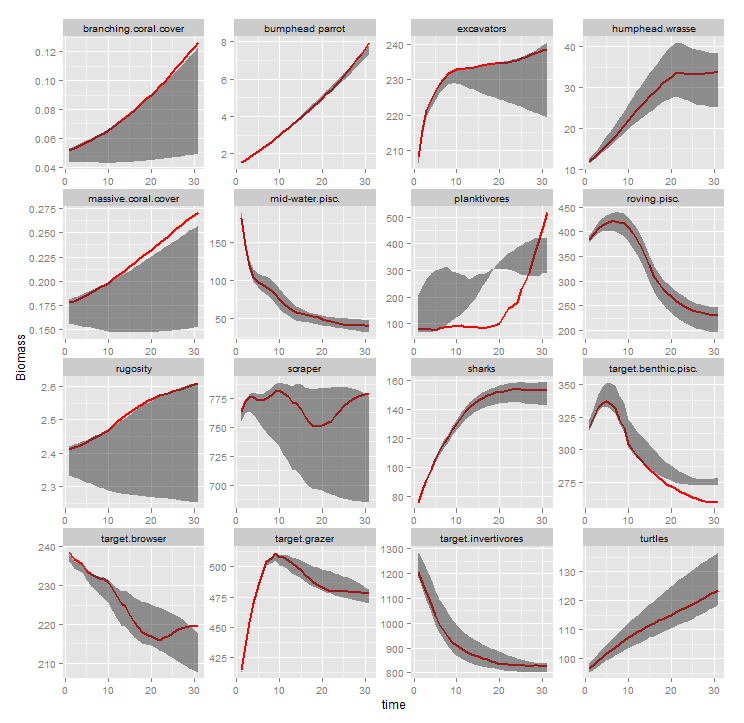


**Fig A17. Sensitivity of selected functional groups to growth rates of large and small phytoplankton**.

The shaded area represents the area between the minimum and maximum values used for these parameters (see S2 Table for parameter values). The red line is the trajectory of the control run.


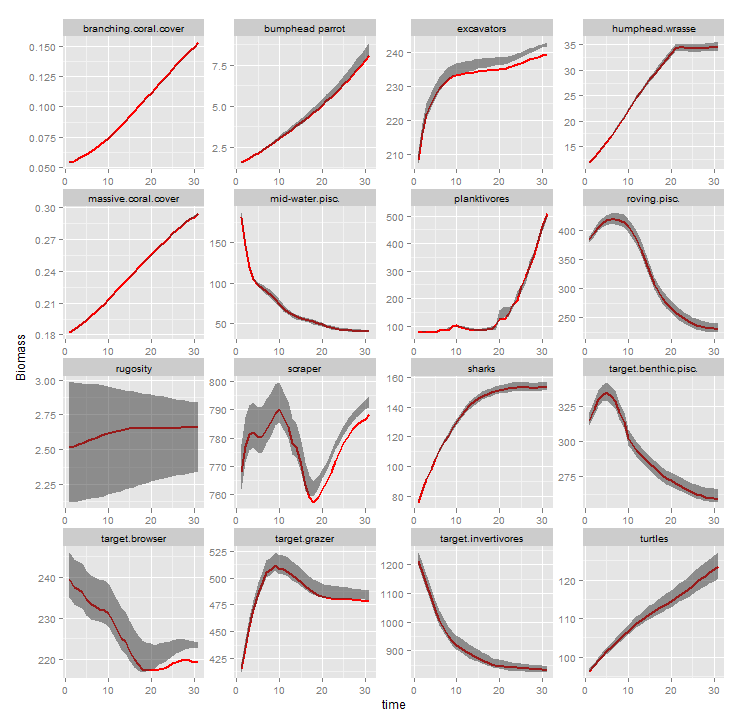


**Fig A18. Sensitivity of selected functional groups to rugosity estimations.**

The shaded area represents the area between the minimum and maximum values used for the mean coral diameter parameters (see S2 Table for parameter values). The red line is the trajectory of the control run.


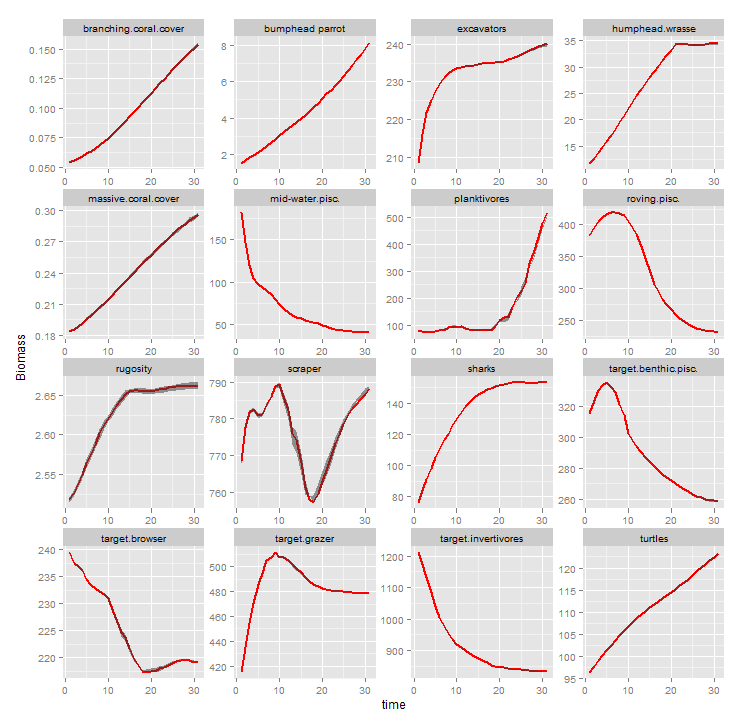


**Fig A19. Sensitivity of selected functional groups to relationship between coral and macroalgae.**

The shaded area represents the area between the minimum and maximum values used for this parameter (see S2 Table for parameter values). The red line is the trajectory of the control run.

Formal skill assessment of the Guam Atlantis model is difficult due to the lack of time-series of observed data for fish stocks and limited time series for coral biomass/cover. For the fish biomass we compared the observed biomass estimates from 2011 visual surveys conducted by CRED with the projected biomass in 2015 after simulating the status quo scenario for 30 years. Skill assessment results show that for fish groups with lower abundance (less than 50 t) model predictions at the end of a 30-year simulation corresponded reasonably well (RMSE = 14) with observations from visual surveys. For the more abundant species, model predictions were consistently above the observed estimates resulting in a very high value of RMSE (198) suggesting that the model has a bias (Fig A20). The largest discrepancy was for scrapers (parrotfish) and grazers (triggerfish, gregory, damselfish), and to a lesser extend for invertivores, planktivores and target grazers (surgeon fish). This result implies that either those fish groups are too productive in our model or that we underestimated the mortality. Since fish productivity responded reasonably to increased fishing mortality (Fig A5), it seems more likely that we underestimated fishing mortality, particularly because the fishery data did not capture all gear types equally well (K. Lowe, NOAA-PIFSC, pers. comm.). Fishing mortality is based on voluntary creel surveys and we did not take into account the boat-based fishery, for a future version of this Guam Atlantis model better fishery estimates are necessary to account for this difference.


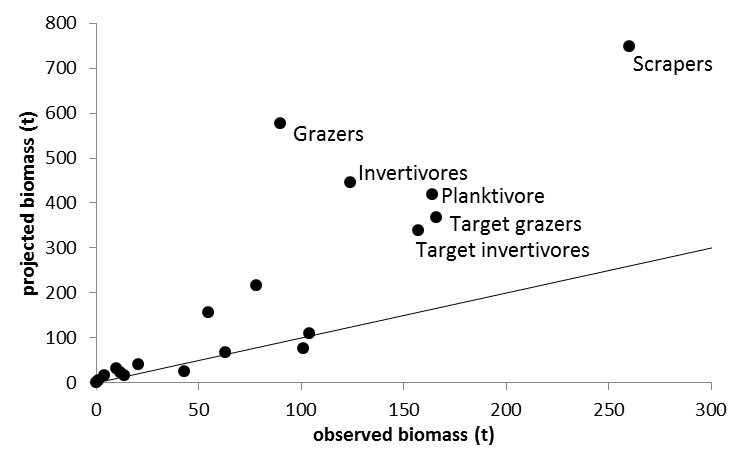


**Fig A20. Observed versus projected biomass of fish groups in 2011**.

Observations are from visual surveys conducted by NOAA Coral Reef Ecosystem Division, projected values are the mean of the last five years at the end of a 30-year run representing 2010–2015.

The model performed much better in projecting coral biomass with a RSME of 0.19. Observed and predicted biomass showed a strong overlap for massive corals (Fig A21).


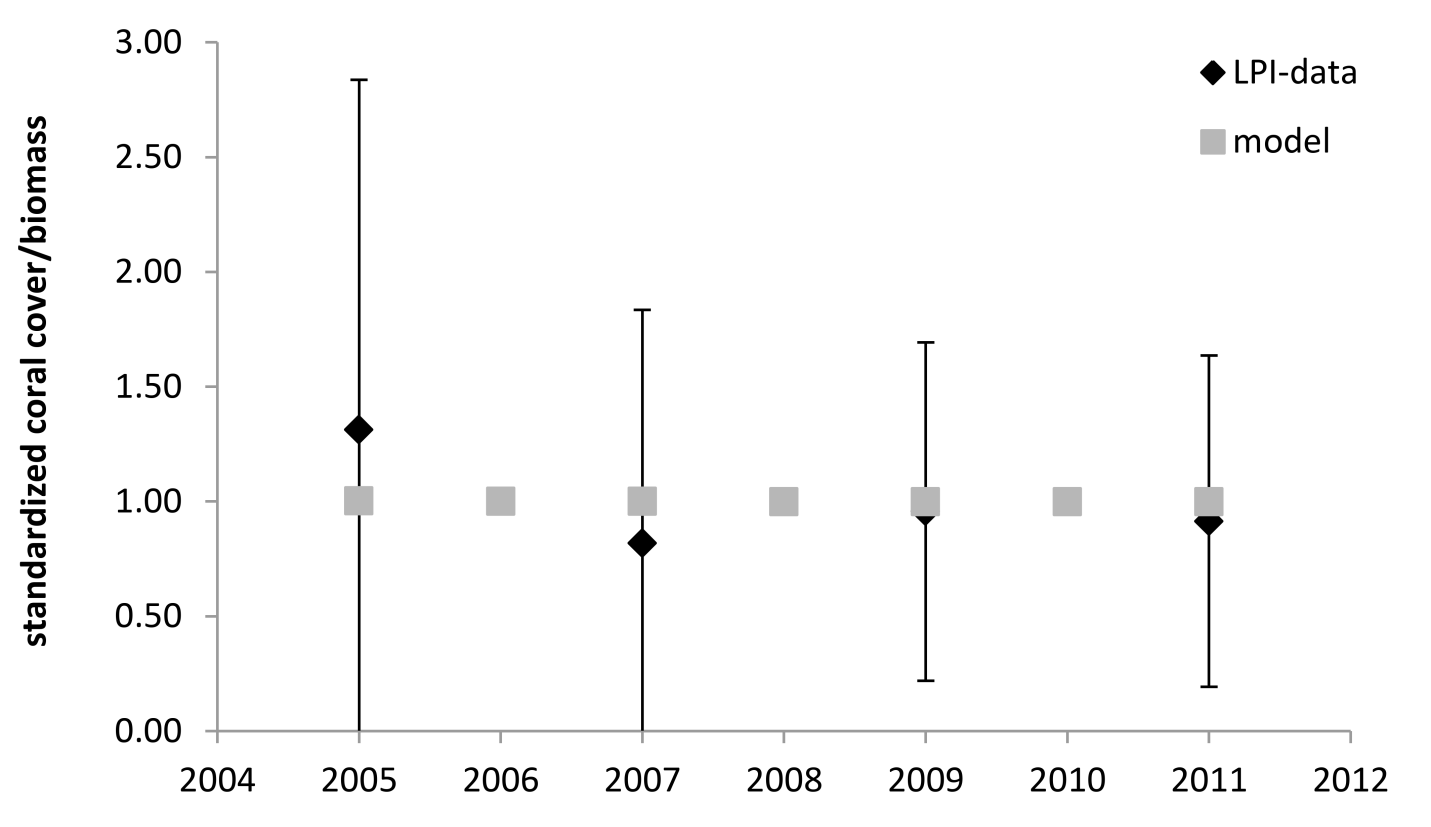


**Fig A21. Observed (blue diamonds) and projected (red squares) biomass of coral over time**. Observations are for all corals from visual line-point-intercept (LPI) surveys conducted by NOAA Coral Reef Ecosystem Division, projected modeled values are the total annual values of massive coral biomass. Both data series are standardized to the mean for comparison.

# Conclusion

The adapted Guam Atlantis model met the three main criteria for Atlantis model development and is stable with plausible biomass trajectories. The paucity of time series of observational data prevented a formal skill assessment, but the model was able to reproduce biomass trajectories after disturbances (e.g., effects of climate change and sediment and nutrient inputs) that corresponded with expectations based on published relationships giving us confidence in the model validity. Additionally, the coral-algae-grazer dynamics are well simulated by the model with model outcomes comparable to expectations in response to low herbivorous biomass as well as the relationship between reef’s structural complexity and its ability to provide refuge for prey fishes. Despite room for improvement (e.g., on historical fisheries catches and alternative predictions of pH and aragonite saturation) the model simulates the complex dynamics within a coral reef ecosystem well. However, in the future uncertainty in model outcome would be better captured by comparing model predictions across a range of parameterizations of the model.

# References

1. Brand E, Kaplan I, Harvey C, Levin P, Fulton E, Hermann A, et al. A spatially explicit ecosystem model of the California Current's food web and oceanography. US Dept. Commerce, NOAA Technical Memorandum NMFS-NWFSC 84 1 45; 2007.

2. Kaplan IC, Burden M, Levin PS, Fulton EA. Fishing catch shares in the face of global change: a framework for integrating cumulative impacts and single species management. Canadian Journal of Fisheries and Aquatic Sciences. 2010;67(12):1968-82.

3. Link JS, Fulton EJ, Gamble RJ. The northeast US application of ATLANTIS: A full system model exploring marine ecosystem dynamics in a living marine resource management context. Progress In Oceanography. 2010;87(1-4):214-34.

4. Ainsworth CH, Kaplan IC, Levin PS, Cudney-Bueno R, Fulton EA, Mangel M, et al. Atlantis model development for the Northern Gulf of California. U.S. Dept. Commer., 2011.

5. Kaplan IC, Horne PJ, Levin PS. Screening California Current fishery management scenarios using the Atlantis end-to-end ecosystem model. Progress In Oceanography. 2012;102(0):5-18. doi: <http://dx.doi.org/10.1016/j.pocean.2012.03.009>.

6. Rodrigues LJ, Grottoli AG, Pease TK. Lipid class composition of bleached and recovering *Porites compressa* Dana, 1846 and *Montipora capitata* Dana, 1846 corals from Hawai'i. Journal of Experimental Marine Biology and Ecology. 2008;358(2):136-43.

7. Gustafsson M. Modelling cnidarian-algae symbiosis, implications of nutrient supply, temperature, and light intensity. Sydney: university of Technology; 2013.

8. Bythell JC. Nutrient uptake in the reef-building coral *Acropora palmata* at natural environmental concentrations. Mar Ecol Prog Ser. 1990;68:1-2.

9. Palardy JE, Rodrigues LJ, Grottoli AG. The importance of zooplankton to the daily metabolic carbon requirements of healthy and bleached corals at two depths. Journal of Experimental Marine Biology and Ecology. 2008;367(2):180-8. doi: 10.1016/j.jembe.2008.09.015.

10. Palardy JE, Grottoli AG, Matthews KA. Effect of naturally changing zooplankton concentrations on feeding rates of two coral species in the Eastern Pacific. Journal of Experimental Marine Biology and Ecology. 2006;331(1):99-107. doi: 10.1016/j.jembe.2005.10.001.

11. Birkeland C, Miller MW, Piniak GA, Eakin CM, Weijerman M, McElhany P, et al. Safety in Numbers? Abundance May Not Safeguard Corals from Increasing Carbon Dioxide. Bioscience. 2013;63(12):967-74. doi: 10.1525/bio.2013.63.12.9.

12. Edmunds PJ, Burgess SC, Putnam HM, Baskett ML, Bramanti L, Fabina NS, et al. Evaluating the causal basis of ecological success within the scleractinia: an integral projection model approach. Marine Biology. 2014;161(12):2719-34.

13. Graham EM, Baird AH, Connolly SR. Survival dynamics of scleractinian coral larvae and implications for dispersal. Coral Reefs. 2008;27(3):529-39.

14. Nugues MM, Bak RPM. Differential competitive abilities between Caribbean coral species and a brown alga: a year of experiments and a long-term perspective Marine Ecology Progress Series. 2006;315:75-86.

15. Melbourne-Thomas J, Johnson C, Fulton E. Regional-scale scenario analysis for the Meso-American Reef system: Modelling coral reef futures under multiple stressors. Ecological Modelling. 2011;222(10):1756-70.

16. Horne P, Kaplan I, Marshall K. Design and parameterization of a spatially explicit ecosystem model of the central California Current. Technical Memo, NMFS-NWFSC-104, Dept. of Commerce, NOAA; 2010. p. 140.

17. Weijerman M, Williams ID, Gutierrez J, Grafeld S, Tibbats B, Davis G. Coral reef-fish biomass trends based on shore-based creel surveys in Guam. Fisheries bulletin. in press.

18. Tsehaye I, Nagelkerke LAJ. Exploring optimal fishing scenarios for the multispecies artisanal fisheries of Eritrea using a trophic model. Ecological Modelling. 2008;212(3-4):319-33. doi: DOI: 10.1016/j.ecolmodel.2007.10.044.

19. Hospital S. Western pacific creel survey program data summary and analysis: Guam, the Commonwealth of the Northern Mariana Islands, and American Samoa. Honolulu, HI: PIFSC Administrative Report, 2015.

20. Baskett ML, Gaines SD, Nisbet RM. Symbiont diversity may help coral reefs survive moderate climate change. Ecological Applications. 2009;19(1):3-17.

21. Smith JE, Shaw M, Edwards RA, Obura D, Pantos O, Sala E, et al. Indirect effects of algae on coral: algae-mediated, microbe-induced coral mortality. Ecology Letters. 2006;9(7):835-45. doi: 10.1111/j.1461-0248.2006.00937.x.

22. Aeby G, Williams G, Franklin E, Haapkyla J, Harvell C. Growth Anomalies on the Coral Genera Acropora and Porites Are Strongly Associated with Host Density and Human Population Size across the Indo-Pacific. PloS ONE. 2011;6(2):e16887.

23. Williams GJ, Price NN, Ushijima B, Aeby GS, Callahan S, Davy SK, et al. Ocean warming and acidification have complex interactive effects on the dynamics of a marine fungal disease. Proceedings of the Royal Society B: Biological Sciences. 2014;281(1778). doi: 10.1098/rspb.2013.3069.

24. Mumby P. The impact of exploiting grazers (Scaridae) on the dynamics of Caribbean coral reefs. Ecological Applications. 2006;16(2):747-69.

25. Jokiel PL, Coles SL. Response of Hawaiian and other Indo-Pacific reef corals to elevated temperature. Coral Reefs. 1990;8(4):155-62.

26. Brown BE. Coral bleaching: causes and consequences. Coral Reefs. 1997;16(5):129-38.

27. Hoegh-Guldberg O. Climate change, coral bleaching and the future of the world's coral reefs. Marine and Freshwater Research. 1999;50(8):839-66.

28. Marshall PA, Baird AH. Bleaching of corals on the Great Barrier Reef: differential susceptibilities among taxa. Coral Reefs. 2000;19(2):155-63.

29. McClanahan TR, Baird AH, Marshall PA, Toscano MA. Comparing bleaching and mortality responses of hard corals between southern Kenya and the Great Barrier Reef, Australia. Marine Pollution Bulletin. 2004;48:327-35.

30. Hoegh-Guldberg O, Mumby PJ, Hooten AJ, Steneck RS, Greenfield P, Gomez E, et al. Coral reefs under rapid climate change and ocean acidification. Science. 2007;318(5857):1737-42. PubMed PMID: ISI:000251616800029.

31. Eakin CM, Lough JM, Heron SF. Climate variability and change: Monitoring data and evidence for increased coral bleaching stress. In: van Oppen MJH, Lough JM, editors. Coral Bleaching. Ecological Studies. 205: Springer Berlin Heidelberg; 2009. p. 41-67.

32. Sotka EE, Thacker RW. Do some corals like it hot? Trends in Ecology & Evolution. 2005;20(2):59-62. doi: <http://dx.doi.org/10.1016/j.tree.2004.11.015>.

33. Levas SJ, Grottoli AG, Hughes A, Osburn CL, Matsui Y. Physiological and Biogeochemical Traits of Bleaching and Recovery in the Mounding Species of Coral <italic>Porites lobata</italic>: Implications for Resilience in Mounding Corals. PLoS ONE. 2013;8(5):e63267. doi: 10.1371/journal.pone.0063267.

34. Berner RA. Activity coefficients of bicarbonate, carbonate and calcium ions in sea water. Geochimica Et Cosmochimica Acta. 1965;29(8):947-65.

35. Edmond JM, Gieskes J. On the calculation of the degree of saturation of sea water with respect to calcium carbonate under< i> in situ</i> conditions. Geochimica Et Cosmochimica Acta. 1970;34(12):1261-91.

36. Eakin CM. A tale of two Enso Events: carbonate budgets and the influence of two warming disturbances and intervening variability, Uva Island, Panama. Bulletin of Marine Science. 2001;69(1):171-86.

37. Langdon C, editor Review of experimental evidence for effects of CO_2_ on calcification of reef builders. Proceedings of the 9th International Coral Reef Symposium; 2002; Bali, Indonesia.

38. Kleypas JA, Feely RA, Fabry VJ, Langdon C, Sabine CL, Robbins LL. Impacts of ocean acidification on coral reefs and other marine calcifiers: A guide for future research. Report of a workshop held 18-20 April 2005, St. Petersburg, FL, sponsored by NSF, NOAA, and the US Geological Survey: 2006.

39. Cohen AL, Holcomb M. Why corals care about ocean acidification: uncovering the mechanism. Oceanography. 2009;22:118-27.

40. Feely RA, Doney SC, Cooley SR. Ocean acidification: present conditions and future changes in a high-CO2 world. Oceanography. 2009;22(4):36-47.

41. Silverman J, Lazar B, Cao L, Caldeira K, Erez J. Coral reefs may start dissolving when atmospheric CO2 doubles. Geophysical Research Letters. 2009;36(5):L05606. doi: 10.1029/2008GL036282.

42. Zeebe RE, Wolf-Gladrow DA. Carbon Dioxide, Dissolved (Ocean). Encyclopedia of Paleoclimatology and Ancient Environments: Springer; 2009. p. 123-7.

43. Hendriks IE, Duarte CM, Álvarez M. Vulnerability of marine biodiversity to ocean acidification: a meta-analysis. Estuarine, coastal and shelf science. 2010;86(2):157-64.

44. Kroeker KJ, Kordas RL, Crim RN, Singh GG. Meta-analysis reveals negative yet variable effects of ocean acidification on marine organisms. Ecology Letters. 2010;13(11):1419-34.

45. Anthony KRN, Maynard JA, Diaz-Pulido G, Mumby PJ, Marshall PA, Cao L, et al. Ocean acidification and warming will lower coral reef resilience. Global Change Biology. 2011;17(5):1798-808. doi: 10.1111/j.1365-2486.2010.02364.x.

46. Harvey BP, Gwynn-Jones D, Moore PJ. Meta-analysis reveals complex marine biological responses to the interactive effects of ocean acidification and warming. Ecology and evolution. 2013;3(4):1016-30. doi: 10.1002/ece3.516.

47. Wittmann AC, Pörtner H-O. Sensitivities of extant animal taxa to ocean acidification. Nature Climate Change. 2013;3(11):995-1001.

48. Eakin CM. Where have all the carbonates gone? A model comparison of calcium carbonate budgets before and after the 1982–1983 El Nino at Uva Island in the eastern Pacific. Coral Reefs. 1996;15(2):109-19. doi: 10.1007/bf01771900.

49. Glynn PW. Bioerosion and coral-reef growth: a dynamic balance. In: Birkeland C, editor. Life and death of coral reefs. New York: Chapman and Hall; 1997. p. 68-95.

50. Jones GP, McCormick MI, Srinivasan M, Eagle JV. Coral decline threatens fish biodiversity in marine reserves. Proceedings of the National Academy of Sciences of the United States of America. 2004;101(21):8251-3. PubMed PMID: ISI:000221652000073.

51. Alvarez-Filip L, Dulvy NK, Gill JA, Côté IM, Watkinson AR. Flattening of Caribbean coral reefs: region-wide declines in architectural complexity. Proceedings of the Royal Society B: Biological Sciences. 2009;276(1669):3019-25. doi: 10.1098/rspb.2009.0339.

52. Ong L, Holland KN. Bioerosion of coral reefs by two Hawaiian parrotfishes: species, size differences and fishery implications. Marine Biology. 2010;157(6):1313-23.

53. Perry C, Edinger E, Kench P, Murphy G, Smithers S, Steneck R, et al. Estimating rates of biologically driven coral reef framework production and erosion: a new census-based carbonate budget methodology and applications to the reefs of Bonaire. Coral Reefs. 2012;31(3):853-68.

54. DeMartini E, Jokiel P, Beets J, Stender Y, Storlazzi C, Minton D, et al. Terrigenous sediment impact on coral recruitment and growth affects the use of coral habitat by recruit parrotfishes (F. Scaridae). Journal of Coastal Conservation. 2013;17:1-13.

55. Graham N, Nash K. The importance of structural complexity in coral reef ecosystems. Coral Reefs. 2013:1-12.

56. Bozec YM, Alvarez‐Filip L, Mumby PJ. The dynamics of architectural complexity on coral reefs under climate change. Global Change Biology. 2014.

57. Blackwood JC, Hastings A, Mumby PJ. A model-based approach to determine the long-term effects of multiple interacting stressors on coral reefs. Ecological Applications. 2011;21(7):2722-33.

58. Hixon MA, Anderson TW, Buch KL, Johnson DW, McLeod JB, Stallings CD. Density dependence and population regulation in marine fish: a large-scale, long-term field manipulation. Ecological Monographs. 2012;82(4):467-89.

59. McCook, Jompa, Diaz P. Competition between corals and algae on coral reefs: a review of evidence and mechanisms. Coral Reefs. 2001;19(4):400-17. doi: 10.1007/s003380000129.

60. Mumby PJ, Harborne AR, Williams J, Kappel CV, Brumbaugh DR, Micheli F, et al. Trophic cascade facilitates coral recruitment in a marine reserve. Proceedings of the National Academy of Sciences. 2007;104(20):8362-7.

61. Baskett ML, Nisbet RM, Kappell CV, Mumby PJ, Gaines SD. Conservation management approaches to protecting the capacity for corals to respond to climate change: a theoretical comparison. Global Change Biology. 2009;16(4):1229-46.

62. Gilmour JP, Smith LD, Heyward AJ, Baird AH, Pratchett MS. Recovery of an isolated coral reef system following severe disturbance. Science. 2013;340(6128):69-71. doi: 10.1126/science.1232310.

63. Baskett ML, Salomon AK. Recruitment facilitation can drive alternative states on temperate reefs. Ecology. 2010;91(6):1763-73.

64. Wolanski E, Richmond RH, McCook L. A model of the effects of land-based, human activities on the health of coral reefs in the Great Barrier Reef and in Fouha Bay, Guam, Micronesia. Journal of Marine Systems. 2004;46(1-4):133-44. doi: DOI: 10.1016/j.jmarsys.2003.11.018.

65. Bellwood DR, Hoey AS, Hughes TP. Human activity selectively impacts the ecosystem roles of parrotfishes on coral reefs. Proceedings of the Royal Society B: Biological Sciences. 2011.

66. Bellwood DR, Hughes TP, Hoey AS. Sleeping functional group drives coral-reef recovery. Current Biology. 2006;16(24):2434-9. doi: 10.1016/j.cub.2006.10.030. PubMed PMID: ISI:000243039800026.

67. Mumby P, Bejarano S, Golbuu Y, Steneck R, Arnold S, van Woesik R, et al. Empirical relationships among resilience indicators on Micronesian reefs. Coral Reefs. 2013:1-14.

68. Mumby P, Hastings A, Edwards H. Thresholds and the resilience of Caribbean coral reefs. Nature. 2007;450(7166):98-101. doi: 10.1038/nature06252.

69. Heenan A, Williams ID. Monitoring herbivorous fishes as indicators of coral reef resilience in American Samoa. PLoS ONE. 2013;8(11):e79604. doi: 10.1371/journal.pone.0079604.

70. Wilkinson C, editor. Status of coral reefs of the world: 2004: Australian Inst. of Marine Science, Townsville; UNEP, Nairobi; Global Coral Reef Monitoring Network, International Coral Reef Initiative, Paris; 2004.

71. Donner SD. Coping with commitment: projected thermal stress on coral reefs under different future scenarios. PLoS One. 2009;4(6).

72. Thompson A, Dolman A. Coral bleaching: one disturbance too many for near-shore reefs of the Great Barrier Reef. Coral Reefs. 2010;29(3):637-48. doi: 10.1007/s00338-009-0562-0.

73. Edwards HJ, Elliott IA, Eakin CM, Irikawa A, Madin JS, Mcfield M, et al. How much time can herbivore protection buy for coral reefs under realistic regimes of hurricanes and coral bleaching? Global Change Biology. 2011;17:2033-48. doi: 10.1111/j.1365-2486.2010.02366.x.

74. Palumbi SR, Barshis DJ, Traylor-Knowles N, Bay RA. Mechanisms of reef coral resistance to future climate change. Science. 2014;344(6186):895-8. doi: 10.1126/science.1251336.

75. Eakin CM. Lamarck was partially right—and that is good for corals. Science. 2014;344(6186):798-9. doi: 10.1126/science.1254136.

76. Graham NA, Jennings S, MacNeil MA, Mouillot D, Wilson SK. Predicting climate-driven regime shifts versus rebound potential in coral reefs. Nature. 2015.

77. Donner SD, Skirving WJ, Little CM, Oppenheimer M, Hoegh-Guldberg O. Global assessment of coral bleaching and required rates of adaptation under climate change. Global Change Biology. 2005;11:2251-65. doi: 10.1111/j.1365-2486.2005.01073.x.

78. McClanahan TR. The relationship between bleaching and mortality of common corals. Marine Biology. 2004;144(6):1239-45.

79. Lombardi MR, Lesser MP, Gorbunov MY. Fast repetition rate (FRR) fluorometry: variability of chlorophyll a fluorescence yields in colonies of the corals, Montastraea faveolata (w.) and Diploria labyrinthiformes (h.) recovering from bleaching. Journal of Experimental Marine Biology and Ecology. 2000;252(1):75-84. doi: <http://dx.doi.org/10.1016/S0022-0981(00)00238-0>.

80. Kvitt H, Rosenfeld H, Zandbank K, Tchernov D. Regulation of apoptotic pathways by *Stylophora pistillata* (Anthozoa, Pocilloporidae) to survive thermal stress and bleaching. PLoS ONE. 2011;6(12):e28665. doi: 10.1371/journal.pone.0028665.

81. Castruccio FS, Curchitser EN, Kleypas JA. A model for quantifying oceanic transport and mesoscale variability in the Coral Triangle of the Indonesian/Philippines Archipelago. Journal of Geophysical Research: Oceans. 2013;118(11):6123-44.

82. Brainard RE, Birkeland C, Eakin CM, McElhany P, Miller MW, Patterson M, et al. Status review report of 82 candidate coral species petitioned under the U.S. Endangered Species Act. Honolulu, HI: 2011.

83. Shaw EC, McNeil BI, Tilbrook B. Impacts of ocean acidification in naturally variable coral reef flat ecosystems. Journal of Geophysical Research: Oceans. 2012;117(C3):C03038. doi: 10.1029/2011jc007655.

84. Kroeker KJ, Kordas RL, Crim R, Hendriks IE, Ramajo L, Singh GS, et al. Impacts of ocean acidification on marine organisms: quantifying sensitivities and interaction with warming. Global Change Biology. 2013;19(6):1884-96. doi: 10.1111/gcb.12179.

85. Comeau S, Carpenter RC, Nojiri Y, Putnam HM, Sakai K, Edmunds PJ. Pacific-wide contrast highlights resistance of reef calcifiers to ocean acidification. Proceedings of the Royal Society B: Biological Sciences. 2014;281(1790). doi: 10.1098/rspb.2014.1339.

86. Holcomb MC, McCorkle DC, Cohen AL. Long-term effects of nutrient and CO2 enrichment on the temperate coral Astrangia poculata (Ellis and Solander 1786). Journal of Experimental Marine Biology and Ecology. 2010;386:27–33.

87. Kurihara H, Ishimatsu A. Effects of high CO< sub> 2</sub> seawater on the copepod (< i> Acartia tsuensis</i>) through all life stages and subsequent generations. Marine Pollution Bulletin. 2008;56(6):1086-90.

88. Langdon C, Atkinson MJ. Effect of elevated pCO2 on photosynthesis and calcification of corals and interactions with seasonal change in temperature/irradiance and nutrient enrichment. Journal of Geophysical Research: Oceans. 2005;110(C9):2156-202. doi: 10.1029/2004jc002576.

89. Golbuu Y, Victor S, Penland L, Idip Jr D, Emaurois C, Okaji K, et al. Palau’s coral reefs show differential habitat recovery following the 1998-bleaching event. Coral Reefs. 2007;26(2):319-32.

90. Burdick D, Brown V, Asher J, Caballes M, Gawel M, Goldman L, et al. Status of coral reef ecosystems of Guam. Guam: Bureau of Statistics and Plans, Guam Coastal Management Program, 2008.

91. Ortiz JC, Bozec Y-M, Wolff NH, Doropoulos C, Mumby PJ. Global disparity in the ecological benefits of reducing carbon emissions for coral reefs. Nature Clim Change. 2014;4(12):1090-4. doi: 10.1038/nclimate2439

<http://www.nature.com/nclimate/journal/v4/n12/abs/nclimate2439.html#supplementary-information>.

92. Kleypas JA, Buddemeier RW, Archer D, Gattuso J-P, Langdon C, Opdyke BN. Geochemical consequences of increased atmospheric carbon dioxide on coral reefs. Science. 1999;284(5411):118-20.

93. Reynaud S, Leclercq N, Romaine-Lioud S, Ferrier-Pagés C, Jaubert J, Gattuso J-P. Interacting effects of CO2 partial pressure and temperature on photosynthesis and calcification in a scleractinian coral. Global Change Biology. 2003;9(11):1660-8. doi: 10.1046/j.1365-2486.2003.00678.x.

94. Jokiel P, Coles S. Effects of temperature on the mortality and growth of Hawaiian reef corals. Marine Biology. 1977;43(3):201-8.

95. Pandolfi JM, Connolly SR, Marshall DJ, Cohen AL. Projecting coral reef futures under global warming and ocean acidification. Science. 2011;333(6041):418-22.

96. Hughes TP. Catastrophes, phase shifts, and large-scale degradation of a Caribbean coral reef. Science-AAAS-Weekly Paper Edition. 1994;265(5178):1547-51.

97. Lapointe BE. Nutrient thresholds for bottom-up control of macroalgal blooms and coral reefs. Limnol Oceanogr. 1997;44:1586-92.

98. Marshell A, Mumby PJ. Revisiting the functional roles of the surgeonfish Acanthurus nigrofuscus and Ctenochaetus striatus. Coral Reefs. 2012;31(4):1093-101. doi: 10.1007/s00338-012-0931-y.

99. Weijerman M, Kaplan IC, Fulton EA, Gorton R, Grafeld S, Brainard R. Design and parameterization of a coral reef ecosystem model for Guam. U.S. Dep. Commer., NOAA Tech. Memo., NOAA-TM-NMFS-PIFSC-43, 2014.

100. Albright R, Mason B, Langdon C. Effect of aragonite saturation state on settlement and post-settlement growth of *Porites astreoides* larvae. Coral Reefs. 2008;27(3):485-90. doi: 10.1007/s00338-008-0392-5. PubMed PMID: ISI:000258085900004.

101. Fulton EA, Smith ADM, Johnson CR. Biogeochemical marine ecosystem models I: IGBEM--a model of marine bay ecosystems. Ecological Modelling. 2004;174(3):267-307. doi: DOI: 10.1016/j.ecolmodel.2003.09.027.

102. Smith JE, Smith CM, Hunter CL. An experimental analysis of the effects of herbivory and nutrient enrichment on benthic community dynamics on a Hawaiian reef. Coral Reefs. 2001;19(4):332-42. PubMed PMID: ISI:000169025400006.

103. Brian EL, Peter JB, Charles SY, Mark ML, Diane SL, Brian K. The relative importance of nutrient enrichment and herbivory on macroalgal communities near Norman's Pond Cay, Exumas Cays, Bahamas: a “natural” enrichment experiment. Journal of Experimental Marine Biology and Ecology. 2004;298. doi: 10.1016/s0022-0981(03)00363-0.

104. Raymundo LJ, Kim K, Redding J, Miller RJ, Pinkerton K, Baker D. Links between deteriorating coral health and sewage pollution of guam reef flats. University of Guam Marine Laboratory Technical Report No131. 2011:p. 24.

105. Williams ID, Polunin NVC, Hendrick VJ. Limits to grazing by herbivorous fishes and the impact of low coral cover on macroalgal abundance on a coral reef in Belize. Marine Ecology Progress Series. 2001;222:187-96.

106. Lokrantz J, Nyström M, Thyresson M, Johansson C. The non-linear relationship between body size and function in parrotfishes. Coral Reefs. 2008;27(4):967-74. doi: 10.1007/s00338-008-0394-3.

107. McClanahan TR. A coral reef ecosystem-fisheries model: impacts of fishing intensity and catch selection on reef structure and processes. Ecological Modelling. 1995;80(1):1-19. doi: Doi: 10.1016/0304-3800(94)00042-g.

108. Wilson SK, Burgess SC, Cheal AJ, Emslie M, Fisher R, Miller I, et al. Habitat utilization by coral reef fish: implications for specialists vs. generalists in a changing environment. Journal of Animal Ecology. 2008;77(2):220-8. doi: 10.1111/j.1365-2656.2007.01341.x.

109. Kerry J, Bellwood D. The effect of coral morphology on shelter selection by coral reef fishes. Coral Reefs. 2012;31:415-24.

110. Ainsworth CH, Mumby P. Coral–algal phase shifts alter fish communities and reduce fisheries production. Global Change Biology. 2014.

111. Friedlander AM, Parrish JD. Habitat characteristics affecting fish assemblages on a Hawaiian coral reef. Journal of Experimental Marine Biology and Ecology. 1998;224(1):1-30. doi: <http://dx.doi.org/10.1016/S0022-0981(97)00164-0>.

112. Paddack MJ, Reynolds JD, Aguilar C, Appeldoorn RS, Beets J, Burkett EW, et al. Recent Region-wide Declines in Caribbean Reef Fish Abundance. Current biology : CB. 2009;19(7):590-5.

113. Graham NAJ, Wilson SK, Jennings S, Polunin NVC, Bijoux JP, Robinson J. Dynamic fragility of oceanic coral reef ecosystems. Proceedings of the National Academy of Sciences of the United States of America. 2006;103(22):8425-9. doi: 10.1073/pnas.0600693103. PubMed PMID: ISI:000238206800024.

114. Coker DJ, Wilson SK, Pratchett MS. Importance of live coral habitat for reef fishes. Reviews in Fish Biology and Fisheries. 2013:1-38.

115. Rogers A, Blanchard JL, Mumby PJ. Vulnerability of Coral Reef Fisheries to a Loss of Structural Complexity. Current Biology. 2014;24(9):1000-5.

116. Wismer S, Hoey A, Bellwood D. Cross-shelf benthic community structure on the Great Barrier Reef: relationships between macroalgal cover and herbivore biomass. Marine Ecology Progress Series. 2009;376:45-54. doi: 10.3354/meps07790.

117. Rasher D, Engel S, Bonito V, Fraser G, Montoya J, Hay M. Effects of herbivory, nutrients, and reef protection on algal proliferation and coral growth on a tropical reef. Oecologia. 2012;169(1):187-98. doi: 10.1007/s00442-011-2174-y.

118. Williams I, Zamzow J, Lino K, Ferguson M, Donham E. Status of coral reef fish assemblages and benthic condition around Guam: A report based on underwater visual surveys in Guam and the Mariana Archipelago, April-June 2011. U.S. Dep Commer, NOAA Tech. Memo., NOAA-TM-NMFS-PIFSC-33, 2012.

119. Stow CA, Jolliff J, McGillicuddy Jr DJ, Doney SC, Allen J, Friedrichs MA, et al. Skill assessment for coupled biological/physical models of marine systems. Journal of Marine Systems. 2009;76(1):4-15.
