# Supplementary material for: An Integrated Coral Reef Ecosystem Model to Support Resource Management under a Changing Climate
Source: PLoS One. 2015 Dec 16;10(12):e0144165. doi: 10.1371/journal.pone.0144165 (PMC4682628; doi:10.1371/journal.pone.0144165)
Supplement: S2 Text — (DOCX) [file pone.0144165.s005.docx]

# S2 Text. Equations for vertebrate movement^[[1]](#footnote-1)^

Atlantis includes vertebrate movement (in terms of the density d of vertebrate group FX, age class i, in polygon j) which can be calculated and updated per quarter (qrt <4) or per year (qrt=4) according to:

$$FX_{i,d,j}=\left\{\begin{matrix} FX_{i,tot}\cdot\left(\vartheta\cdot\left(FX_{j,qrt+1,FX}^{D}-FX_{j,qrt,FX}^{D}\right)+FX_{j,qrt,FX}^{D}\right)\text{ , qrt}<4 \\ FX_{i,tot}\cdot\left(\vartheta\cdot\left(FX_{j,1,FX}^{D}-FX_{j,qrt,FX}^{D}\right)+FX_{j,qrt,FX}^{D}\right)\text{ , qrt}=4 \end{matrix}\right.$$

where *FX*_i,tot_ is the total number of *FX* in age class *i* in the entire ecosystem (that is the sum over all polygons); $\vartheta$ is the proportion of the current quarter of the year which has already passed; *FX^D^_j,qrt,FX_* is the proportion of the population of *FX* found in polygon *j* in the *qrt* quarter of the year.

Fish can move based on forage conditions (food availability) or due to density dependent conditions and the following formulations are used:

$$G_{\text{FX,i,j,potential}}=\left\{\begin{matrix} \text{g}_{\text{roc\_mult}}\cdot G_{\text{FX,i,j}}\text{ , }G_{\text{FX,i,j}}>\text{g}_{\text{thesh}} \\ \frac{G_{\text{FX,i,j}}}{\text{g}_{\text{roc\_mult}}}\text{ , otherwise} \end{matrix}\right.$$

$$G_{\text{FX,i,tot}}=\sum_{\text{all j}} G_{\text{FX,i,j}}$$

$$FX_{_{i,d,j,t}}^{D}=\frac{G_{\text{FX,i,j,potential}}\cdot d_{\mathrm{cov}er}\cdot\delta_{depth}\cdot FX_{i,d,j,t,other}^{D}}{G_{\text{FX,i,tot}}}$$

$$FX_{\text{i,d,j}}=FX_{\text{i,tot}}\cdot\left(FX_{i,vel}\cdot\left(FX_{i,d,j,t}^{D}-FX_{i,d,j,t-1}^{D}\right)+FX_{i,d,j,t-1}^{D}\right)$$

where *G_FX,i,j,potential_* is a measure of the potential attractiveness of the polygon *j* based on the available forage; *G_FX,i,j_* is calculated as *G*_CX_ in the general vertebrate growth equation:

*g_roc_mult_* is a constant reflecting how much more attractive a site is with sufficient food sources to support *FX_i_* over a site with poor food resources; g_thresh_ is the potential growth rate (as an index of the quality of the resources) where *FX_i_* switch from finding the site desirable to undesirable; *d_cover_* is the relative cover in the polygon for the group *FX_i_* (set to one for all groups that are not habitat-dependent); and *δ*_depth_ is a depth limitation factor for the group *FX_i_*. *ε*_CX_ is the growth efficiency of *CX* when feeding on live prey; *ε*_CX,j_ the efficiency when feeding on detritus (DL treated separately to DR), *δ*_depth_ and *δ*_depth_ are space and oxygen limitation factors.

To take into account other pressures on fish movement (such as seasonal or spawning migration) the calculation of the proportion *FX^D^_i,d,j,t_* is weighted by the ideal distribution for those other migration factors and the final distribution is then determined by interpolating between the current distribution and the ideal distribution (taking the maximum swim speed of the vertebrate into account so that individuals cannot move further than they could actually swim in reality). These *FX_i,d,j_* values are then normalised so that their sum is one. If a vertebrate group is site attached then it only moves vertically at most, and if the group employs maternal care then the movement scheme is calculated for the mothers and then applied to them and the juvenile age classes.

1. Text largely taken with permission from supplementary material by Griffith, G. P., E. A. Fulton, R. Gorton, and A. J. Richardson. 2012. Predicting Interactions among Fishing, Ocean Warming, and Ocean Acidification in a Marine System with Whole-Ecosystem Models. Conservation Biology **26**:1145-1152. [↑](#footnote-ref-1)
